# Supplementary material for: A progeroid syndrome caused by a deep intronic variant in TAPT1 is revealed by RNA/SI‐NET sequencing
Source: EMBO Mol Med. 2023 Jan 18;15(2):e16478. doi: 10.15252/emmm.202216478 (PMC9906387; doi:10.15252/emmm.202216478)
Supplement: Supplementary file 1 — Expanded View Figures PDF [file EMMM-15-e16478-s009.pdf]

Expanded View Figures

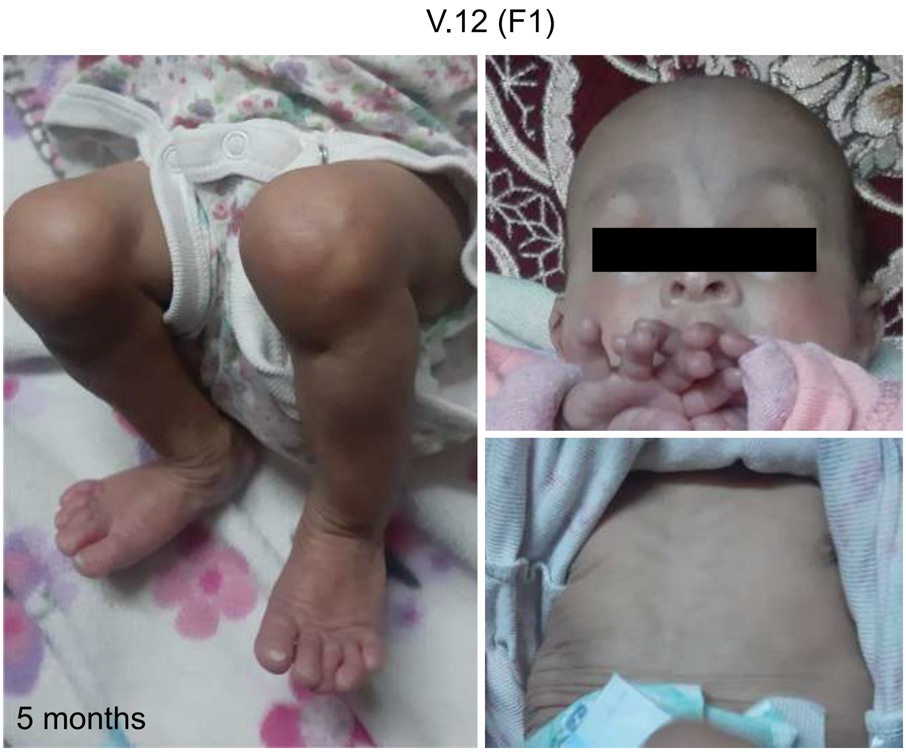

Figure EV1. Clinical pictures of the affected V.12 (F1) individual.

The patient presented with multiple abnormalities including bone and joint deformities, pectus excavatum, plagiocephaly microphthalmia and bilateral hypotropia. Moreover, she had apparent dysmorphic facial features such as a depressed nasal bridge and low set of ears.

Figure EV2. Overlap analysis from homozygosity mapping and RNA-seq data revealed TAPT1 as the only candidate gene.

A List of the 39 candidate genes located in the mapped Chr. 4 IBD locus.

B Lists of the top 10 significantly downregulated (left, blue) and upregulated (right, red) genes obtained from our RNA-seq differential expression analysis.

C Expression changes (x-axis, log2FC) for genes with at least one alternative splicing event (skipped exon (SE), retained exon (RE), mutually exclusive exon (MXE), alternative 3′ or 5′ splice site (A3SS and A5SS) and retained intron (RI)).

D (Top) Venn diagram displaying overlapping genes between the Chr. 4 IBD candidate locus, and the top 10 upregulated and downregulated genes from our RNA-seq data analysis. (Bottom) Venn diagram showing the overlapping genes between the differentially expressed set and the alternative spliced set from our RNA-seq data analysis. *TAPT1* appears as the only overlapping gene in both diagrams.

 © 2023 The Authors

A

List of 39 genes in mapped Chr. 4 IBD candidate locus			
MIR572	*MIR5091*	*FAM200B*	*SNORA75B*
HS3ST1	*LINC01182*	*BST1*	*QDPR*
LINC02360	*LINC01085*	*CD38*	*CLRN2*
MIR12113	*LINC00504*	*FGFBP1*	*LAP3*
LINC02270	*CPEB2-DT*	*FGFBP2*	*DCAF16*
RAB28	*CPEB2*	*PROM1*	*NCAPG*
LINC01097	*C1QTNF7-AS1*	*TAPT1-AS1*	*MED28*
NKX3-2	*C1QTNF7*	*TAPT1*	*FAM184B*
LINC01096	*CC2D2A*	*LDB2*	*LCORL*
BOD1L1	*FBXL5*	*LINC02493*	

B

List of top significant downregulated genes			List of top significant upregulated genes		
**Gene name**	**-log10 (padj)**	**Log2 fold change**	**Gene name**	**-log10 (padj)**	**Log2 fold change**
RARRES2	23.2	-3.57	*CTC-575D19.1*	55.31	5.65
DHRS3	16.25	-1.59	*ZIC1*	52.13	4.47
SULF1	14.65	-3.15	*DES*	25.15	3.83
HLA-H	12.86	-4.09	*RP11-565J7.3*	21.25	3.42
TAPT1	10.1	-2.47	*NTNG1*	12.88	2.36
IKZF2	8.54	-1.95	*POSTN*	9.84	2.51
BEX1	8.39	-2.86	*ZIC4*	8.2	3.77
BAMBI	8.28	-3.17	*BCHE*	7.07	4.34
SRGN	7.62	-3.88	*DNM1*	6.09	2.29
LMOD1	6.51	-1.45	*PLXNC1*	5.48	3.86

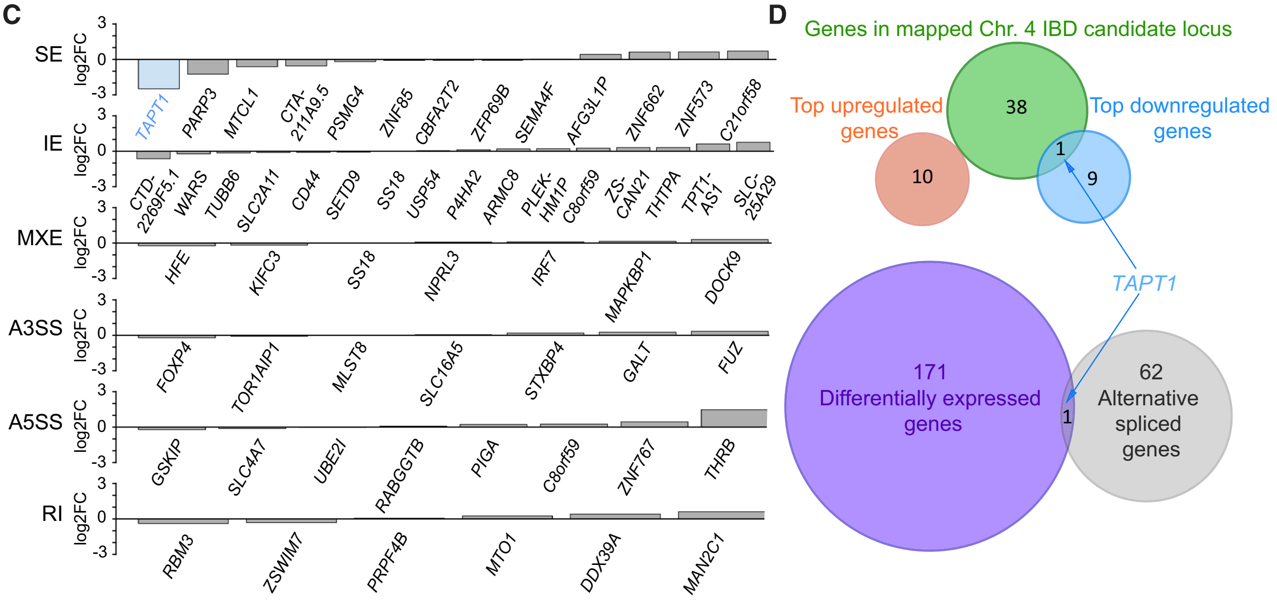

Figure EV2.

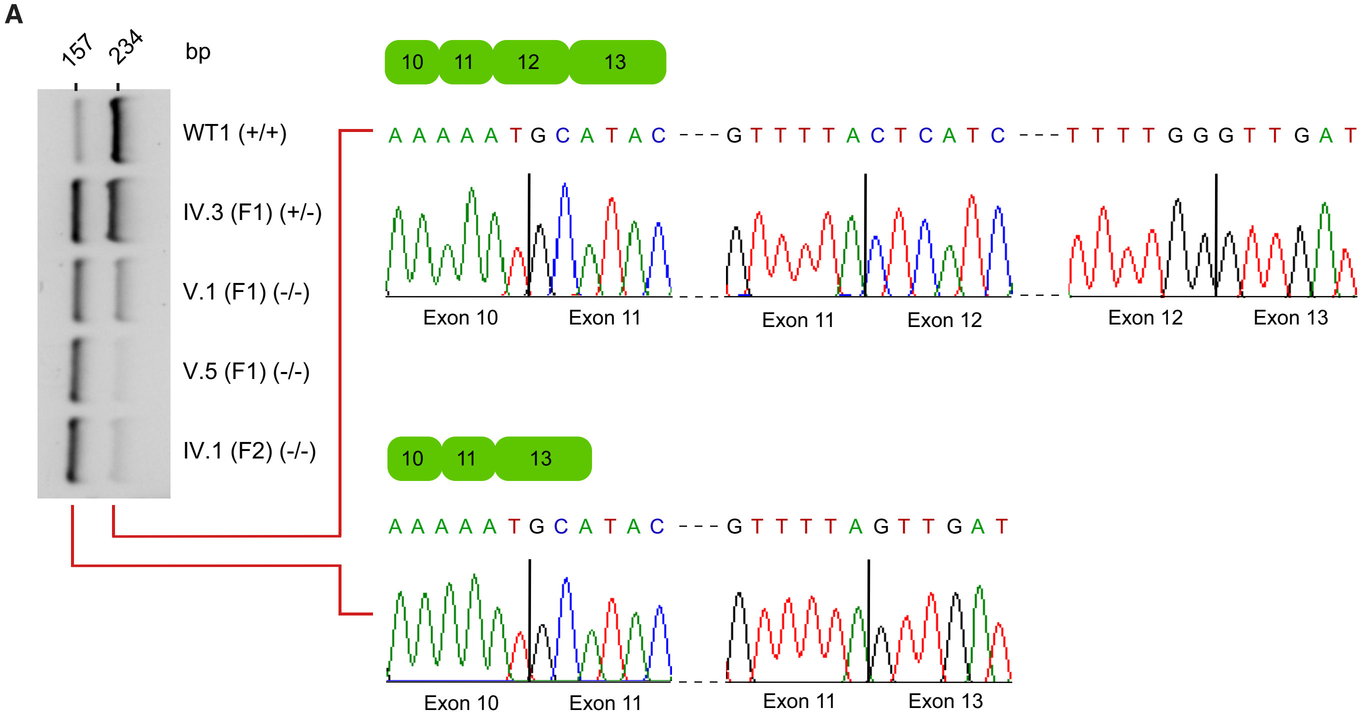

Figure EV3. Elevated numbers of exon-12 skipped transcripts in the patient cells.

A RT–PCR analysis of endogenous *TAPT1* splicing products. To check exon 12 skipping, RT–PCR was performed using primers targeting exon 10 and exon 13 in one WT (WT1), one heterozygous carrier (IV.3 (F1)) and 3 patients (V.1 (F1), V.5 (F1), IV.1 (F2)). The data showed the presence of normal (234 bps) and exon 12-skipped (157 bps) products in all tested samples. However, the truncated transcripts constitute the majority of products in the patient cells. Interestingly, the intensity of 2 bands is rather same in the heterozygous (IV.3 (F1)) sample. Sanger sequencing confirmed the accuracy of RT–PCR products.

Source data are available online for this figure.

Figure EV4. *TAPT1* c.1237-52 G>A mutation and *TAPT1-AS1* show no observable regulatory activity on *TAPT1* mRNA stability and gene expression, respectively.

A qPCR analysis of *c-MYC* and *TAPT1* expression in WT1 and V.1 (F1) primary fibroblasts treated with actinomycin D (ActD) in different time points. ActD was used to check mRNA stability by inhibiting transcription. *c-MYC* was considered as positive control with a short half-life. The results showed that *c-MYC* mRNA level dramatically decreased after 1.5 h treatment (~ x2), whereas the *TAPT1* transcript level is unchanged. qPCR assays involved three technical replicates per sample per time point. For each graph, fold change relative to non-treated condition is plotted as mean ± SD. Asterisks indicate conventional statistical significance (Student's *t*-test; n.s. *P*-value > 0.05, *****P*-value < 0.0001).

B Knockdown of *TAPT1-AS1* transcript using two different GapmeRs (1 and 2) in WT (WT1) and patient (IV.1 (F2)) primary dermal fibroblasts. A non-targeted (NT) GapmeR was used as control. qPCR analysis of *TAPT1-AS1* (top) and *TAPT1* (bottom) transcript levels in the GapmeR-transfected cells. Results show the successful knockdown of *TAPT1-AS1* by both GapmeRs 1 and 2 compared with the control NT GapmeR. However, *TAPT1* mRNA levels are unaltered in both WT and patient cells. Fold change relative to WT1-Control NT GapmeR is plotted as mean ± SD of three technical replicates. Asterisks indicate conventional statistical significance (Student's *t*-test; n.s. *P*-value > 0.05, ***P*-value < 0.01, ****P*-value < 0.001).

C Western blotting of protein extracts from the GapmeR-transfected cells, probing for TAPT1 (Sigma, HPA042567 antibody). Data shows that TAPT1 protein levels are unaffected by the knockdown of *TAPT1-AS1*. GAPDH was used as loading control.

D, E Immunofluorescence staining using two different TAPT1 commercial antibodies (A: Sigma, HPA042567; B: Sigma, HPA048658) in WT1 and IV.1 (F2) primary dermal fibroblasts. Similar fluorescent signal was detected in WT and TAPT1-null cells in both cases. TAPT1 commercial antibodies are unsuitable for immunofluorescence experiments. Scale bar represents 10 μm.

Source data are available online for this figure.

 © 2023 The Authors

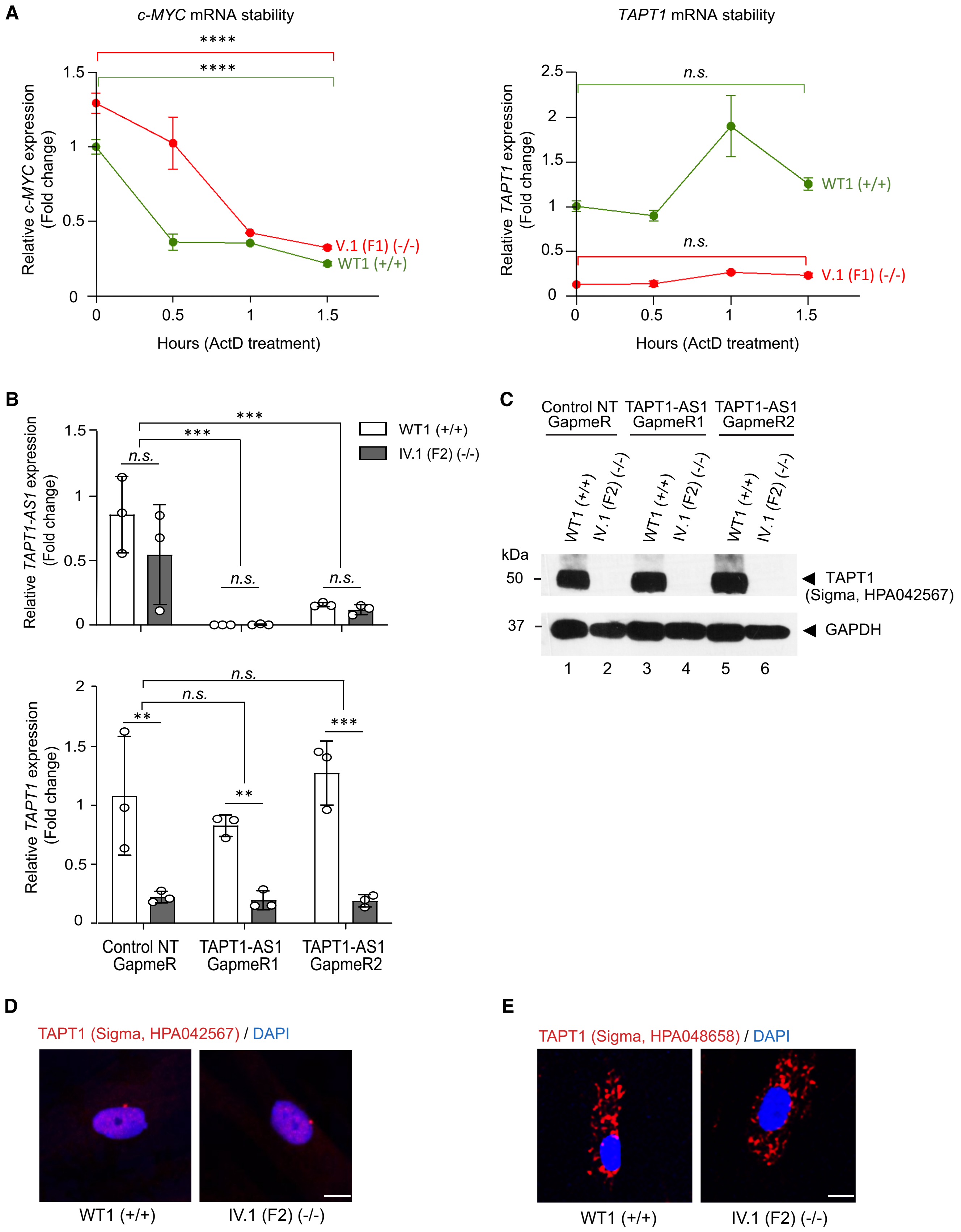
A
c-MYC mRNA stability
****
****
Relative c-MYC expression (Fold change)
1.5
1
0.5
0
0
0.5
1
1.5
Hours (ActD treatment)
V.1 (F1) (-/-)
WT1 (+/+)
TAPT1 mRNA stability
n.s.
n.s.
Relative TAPT1 expression (Fold change)
2.5
2
1.5
1
0.5
0
Hours (ActD treatment)
WT1 (+/+)
V.1 (F1) (-/-)
B
Relative TAPT1-AS1 expression (Fold change)
***
***
n.s.
n.s.
n.s.
WT1 (+/+)
IV.1 (F2) (-/-)
Relative TAPT1 expression (Fold change)
n.s.
n.s.
**
**
***
Control NT GapmeR
TAPT1-AS1 GapmeR1
TAPT1-AS1 GapmeR2
C
Control NT GapmeR
TAPT1-AS1 GapmeR1
TAPT1-AS1 GapmeR2
WT1 (+/+)
IV.1 (F2) (-/-)
kDa
50
37
TAPT1 (Sigma, HPA042567)
GAPDH
1
2
3
4
5
6
D
TAPT1 (Sigma, HPA042567) / DAPI
WT1 (+/+)
IV.1 (F2) (-/-)
E
TAPT1 (Sigma, HPA048658) / DAPI
WT1 (+/+)
IV.1 (F2) (-/-)

Figure EV4.

Figure EV5. SI-NET-seq analysis data.

A Lists of the top 10 genes with significantly decreased (left, blue) or increased (right, red) RNA Pol II occupancy from our SI-NET-seq analysis.

B High Pearson's correlation coefficients ($r \geq 0.96$) between replicates of Pol II gene occupancy indicate the reproducibility of SI-NET-seq measurements. Asterisks indicate conventional statistical significance (Student's *t*-test; ****P*-value < 0.001).

C Volcano plot showing genes with an altered occupancy of transcriptionally engaged Pol II in the heterozygous parent (IV.3 (F1)) compared with WT (WT1 and WT2) primary fibroblast cells. The y-axis shows the −log10 *P*-value, whereas the x-axis indicates the log2 fold change value for the Pol II occupancy. The Pol II density is increased in 149 genes (red dots) and decreased in 21 genes (blue dots). The yellow dot represents *TAPT1*.

D Bubble plot showing enrichment of collagen and extracellular matrix (ECM) pathways from the integrated Reactome pathway analysis from patients (red circles) and heterozygous parent (green circles) SI-NET-seq data. Enriched pathways are indicated on the y-axis, and the corresponding *P*-values are shown on the x-axis. The size of the circles represents the number of altered genes from each pathway.

E Pol II occupancy changes (log) of genes associated with enriched collagen and extracellular matrix (ECM) pathways measured from patients (−/−) and heterozygous parent (+/−) SI-NET-seq data. Significant changes are highlighted (orange). Bold and underlined genes are shared between patients and heterozygous parent.

Source data are available online for this figure.

© 2023 The Authors

A

List of top genes with decreased RNA Pol II occupancy		
Gene name	-log10 (padj)	Log2 fold change
ENOPH1	41.9	-4.96
COMP	27.55	-4.29
CRLF1	16.86	-5.11
PTPRE	13.98	-4
ANGPTL4	12.1	-3.15
COL15A1	12.04	-2.36
CLEC2A	11.94	-5.46
MMP1	11.55	-4.92
PTGS1	11.15	-3.38
SHISAL1	11.12	-2.72

List of top genes with increased RNA Pol II occupancy		
Gene name	-log10 (padj)	Log2 fold change
INHBA	12.69	2.74
CEMIP	12.4	2.64
NR2F1-AS1	12.04	2.91
ADGRD1	10.51	2.91
NTNG1	10.25	3.18
LNX1	9.86	2.1
PTGS2	9.3	2.69
SSC5D	9.04	2.53
KCND3	8.93	3.03
TRPC4	8.44	3.29

B

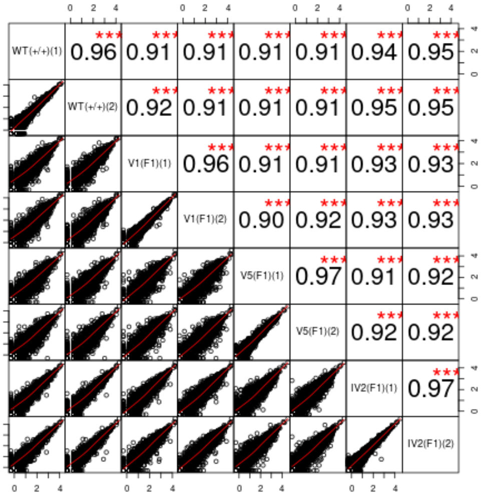

C

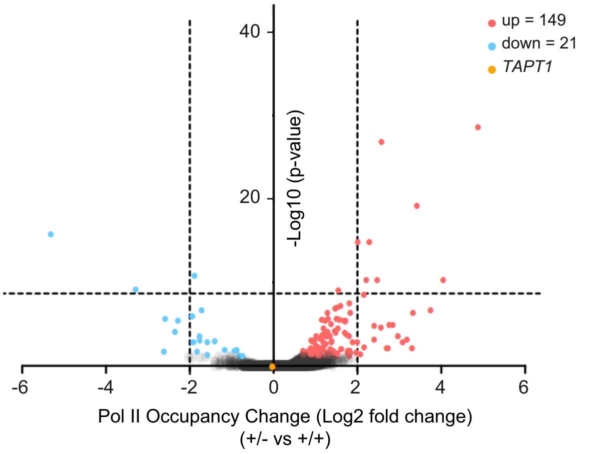

D

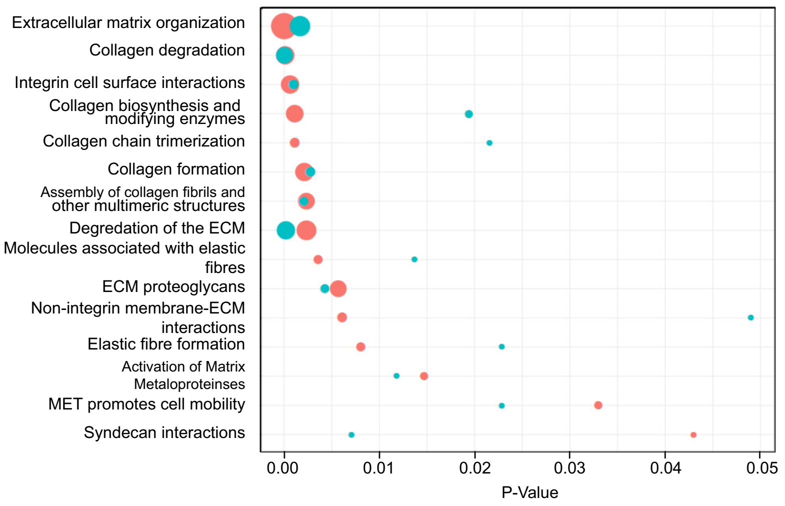

E

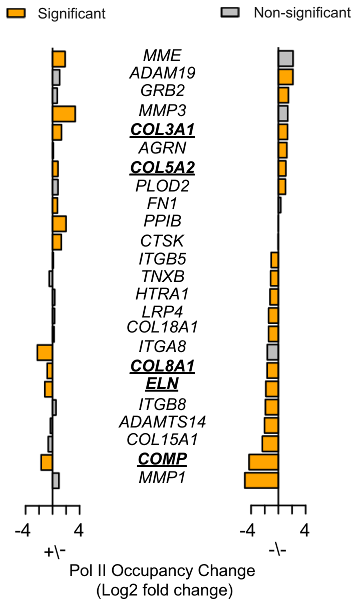

Figure EV5.
