# Supplementary material for: A progeroid syndrome caused by a deep intronic variant in TAPT1 is revealed by RNA/SI‐NET sequencing
Source: EMBO Mol Med. 2023 Jan 18;15(2):e16478. doi: 10.15252/emmm.202216478 (PMC9906387; doi:10.15252/emmm.202216478)
Supplement: Supplementary file 2 — Table EV1 [file EMMM-15-e16478-s002.docx]

Table EV1. List of primers and LNA GapmeR sequences

**Genotyping primer sequences**

| Gene | Primer | Sequence |
| --- | --- | --- |
| *TAPT1* | TAPT1-F1 | GGAAACCCCTGGCATAGACT |
|  | TAPT1-R1 | TGTGGACACGTGTAGCAAGA |
| *TAPT1* | TAPT1-F2 | CTGCAATGGCAGTCTTTTATTC |
|  | TAPT1-R2 | GGTTTCACAAGCAGGCAGAT |
|  |  |  |

**LNA GapmeR sequences**

| LNA GapmeR name | Sequence |
| --- | --- |
| *TAPT1-AS1* GapmeR 1 | GTTCCATCTCTTCTG |
| *TAPT1-AS1* GapmeR 2 | TCACTTACCTTCATGT |
|  |  |

**QPCR primer sequences**

| Gene | Primer | Sequence |
| --- | --- | --- |
| *TAPT1* | TAPT1-QPCR-F1 | AGACGCTGGGCTTCTACGA |
| *TAPT1* | TAPT1-QPCR-R1  TAPT1-QPCR-F2  TAPT1-QPCR-R2 | CCCTCTTGTTAGTTCAGCACTG  TGCTGAACTAACAAGAGGGTACT  TGAACACATACAAAAACGCATCC |
| *TAPT1-AS1* | TAPT1-AS1-QPCR-F1 | ATTTGGGCAAGAAGGAGCTT |
| *TAPT1-AS1* | TAPT1-AS1-QPCR-R1  TAPT1-AS1-QPCR-F2  TAPT1-AS1-QPCR-R2 | GGAGCTCCCTAAGGGCTAGA  CACCAGGCACTGCAATAAGA  CGACAGCATCGTCTCAAAGA |
| *RARRES2* | RARRES2-QPCR-F | AGAAACCCGAGTGCAAAGTCA |
|  | RARRES2-QPCR-R | AGAACTTGGGTCTCTATGGGG |
| *ZIC1* | ZIC1-QPCR-F | CACGCGGGACTTTCTGTTC |
|  | ZIC1-QPCR-R | TGCCCGTTGACCACGTTAG |
| *ZIC4* | ZIC4-QPCR-F | CCCTTCAGATGCGAGTTCGAG |
|  | ZIC4-QPCR-R | GTATGGCTTGTCGCTAGTGTG |
| *BIP* | BIP-QPCR-F | TGTTCAACCAATTATCAGCAAACTC |
|  | BIP-QPCR-R | TTCTGCTGTATCCTCTTCACCAGT |
| *ATF4* | ATF4-QPCR-F | GTTCTCCAGCGACAAGGCTA |
|  | ATF4-QPCR-R | ATCCTGCTTGCTGTTGTTGG |
| *CHOP* | CHOP-QPCR-F | AGAACCAGGAAACGGAAACAGA |
|  | CHOP-QPCR-R | TCTCCTTCATGCGCTGCTTT |
| *P4HB* | P4HB-QPCR-F | TCCTGGAGGGCAAAATCAAG |
|  | P4HB -QPCR-R | GGCATAGAACTCCACAAAGACG |
| *PDIA6* | PDIA6-QPCR-F | TTCTATGCTCCTTGGTGTGG |
|  | PDIA6-QPCR-R | GCCAGAACCTGATTGACTGTAG |
| *XBP1* (total) | XBP1 (total)-QPCR-F | TGGCCGGGTCTGCTGAGTCCG |
|  | XBP1 (total)-QPCR-R | ATCCATGGGGAGATGTTCTGG |
| *XBP1* (spliced) | XBP1 (spliced)-QPCR-F | CTGAGTCCGAATCAGGTGCAG |
|  | XBP1 (spliced)-QPCR-R | ATCCATGGGGAGATGTTCTGG |
| *GAPDH* | GAPDH-QPCR-F | CGACAGTCAGCCGCATCTT |
|  | GAPDH-QPCR-R | CCCCATGGTGTCTGAGCG |

**Minigene assay primer sequences**

| Gene | Primer | Sequence |
| --- | --- | --- |
| *TAPT1*  (Cloning) | EcoR1-TAPT1-F | ACGTACGTGAATTCTACCACAGAAGGTTTCACAAGC |
|  | BamH1-TAPT1-R | ACGTACGTGGATCCTTTACTGTATGTCTCCCCCAACCAAA |
| *TAPT1*  *(*Site directed mutagenesis) | TAPT1-SDM-F | CAAAAACACACAAACAGAACACACAACCACAGAGTTATTAGTTAATACTTAAAAA |
|  | TAPT1-SDM-R | TTTTTAAGTATTAACTAATAACTCTGTGGTTGTGTGTTCTGTTTGTGTGTTTTTG |
| pSPL3 vector  (RT-PCR) | SD6 | TCTGAGTCACCTGGACAACC |
|  | SA2 | ATCTCAGTGGTATTTGTGAGC |
