# Supplementary figures and images for: A progeroid syndrome caused by a deep intronic variant in TAPT1 is revealed by RNA/SI‐NET sequencing
Source: EMBO Mol Med. 2023 Jan 18;15(2):e16478. doi: 10.15252/emmm.202216478 (PMC9906387; doi:10.15252/emmm.202216478)

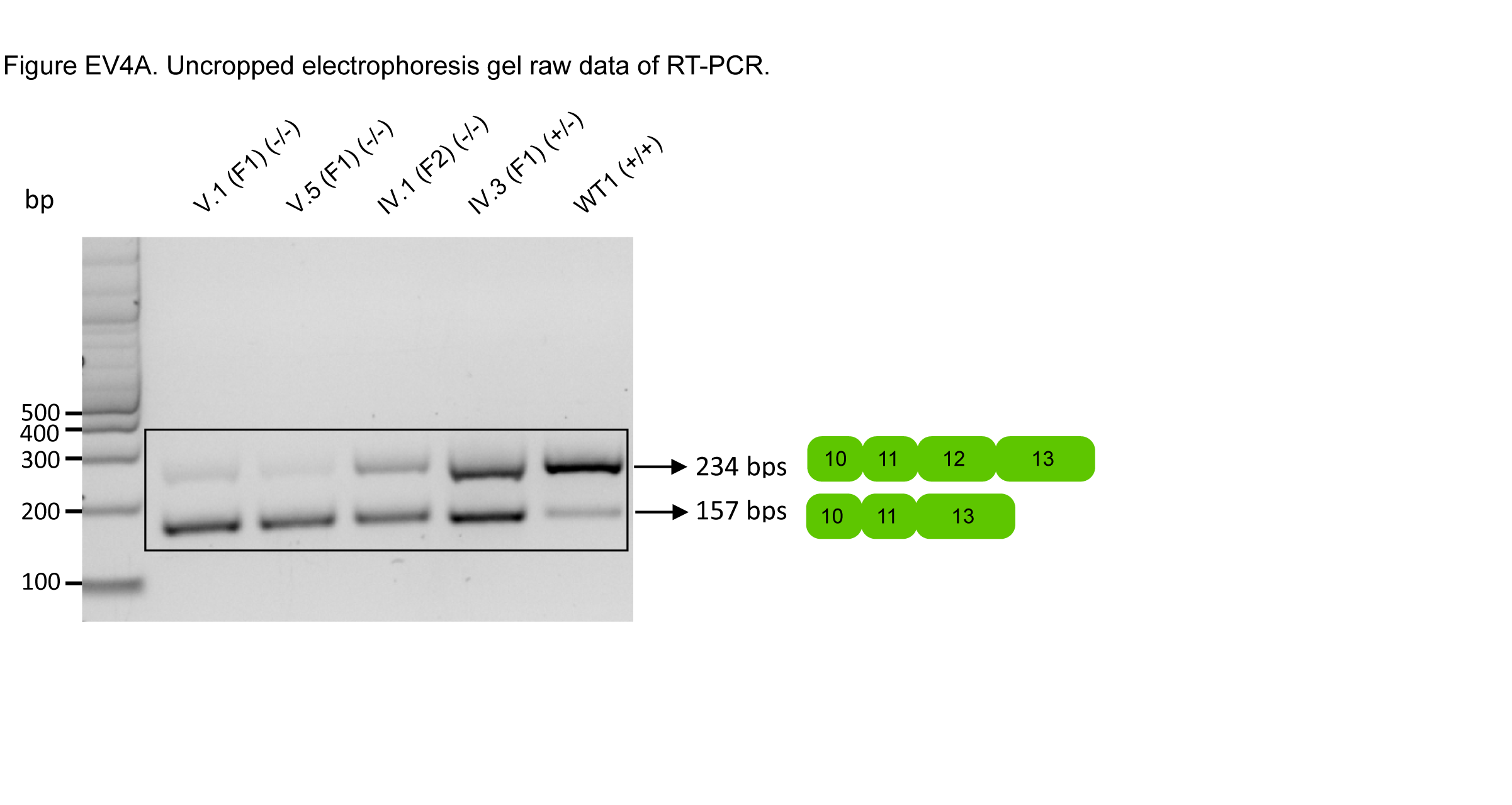

Supplement: Supplementary file 6 — Source Data for Expanded View [file EMMM-15-e16478-s001.zip › Expanded view source data/Figure EV3A (gel electrophoresis)/RT-PCR gel electrophoresis.tif]

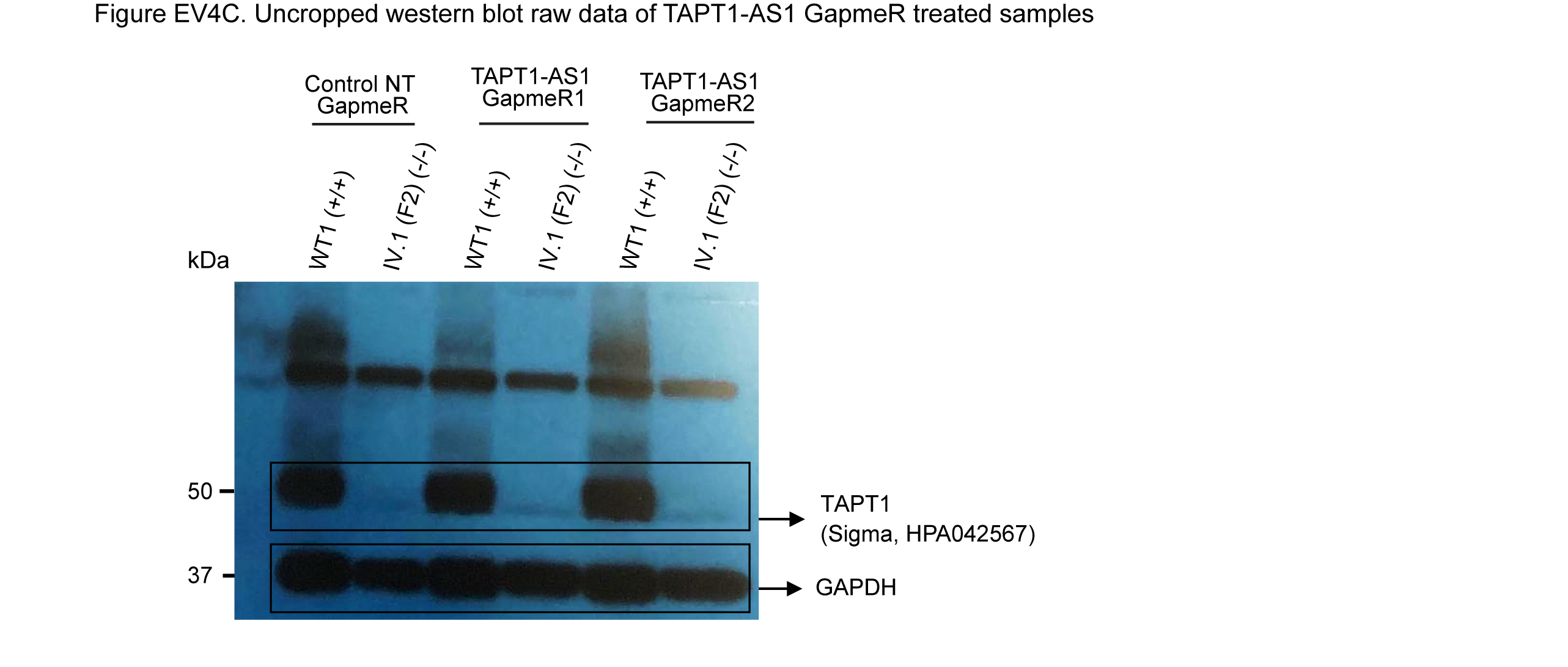

Supplement: Supplementary file 6 — Source Data for Expanded View [file EMMM-15-e16478-s001.zip › Expanded view source data/Figure EV4C (blot)/GapmeR western blot.tif]

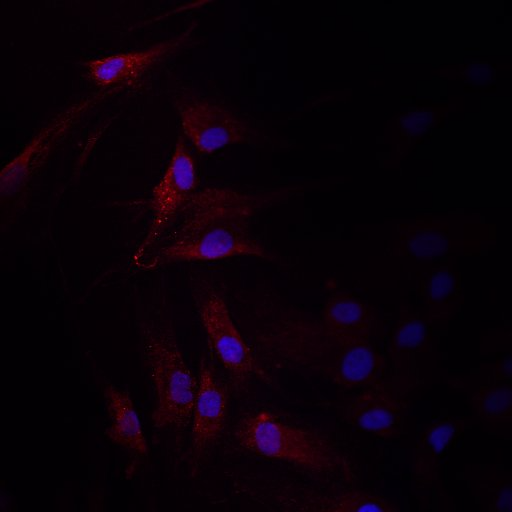

Supplement: Supplementary file 6 — Source Data for Expanded View [file EMMM-15-e16478-s001.zip › Expanded view source data/Figure EV5 (Microscopic images)/EV5B/WT1 (+_+).tiff]

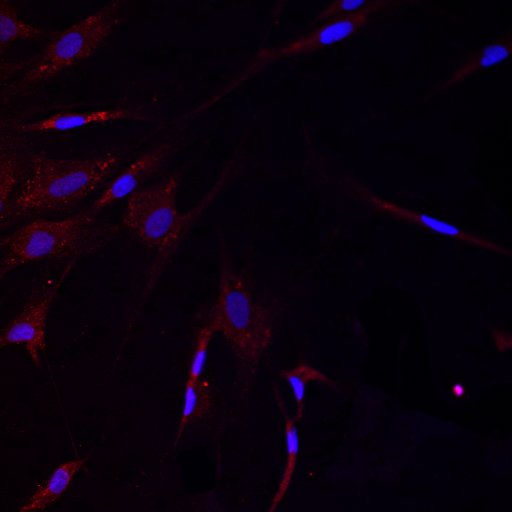

Supplement: Supplementary file 6 — Source Data for Expanded View [file EMMM-15-e16478-s001.zip › Expanded view source data/Figure EV5 (Microscopic images)/EV5B/IV.1(F2)(-_-).tiff]

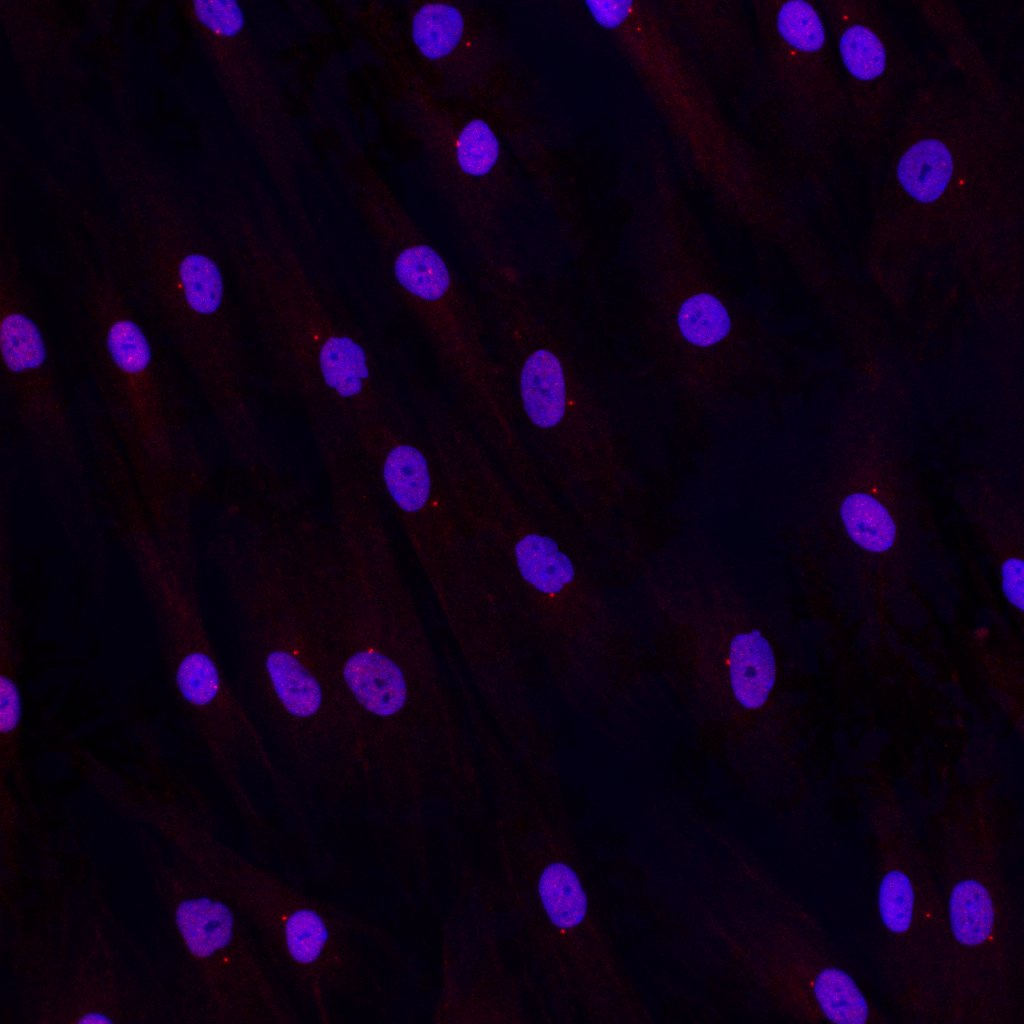

Supplement: Supplementary file 6 — Source Data for Expanded View [file EMMM-15-e16478-s001.zip › Expanded view source data/Figure EV5 (Microscopic images)/EV5A/WT1(+_+).tiff]

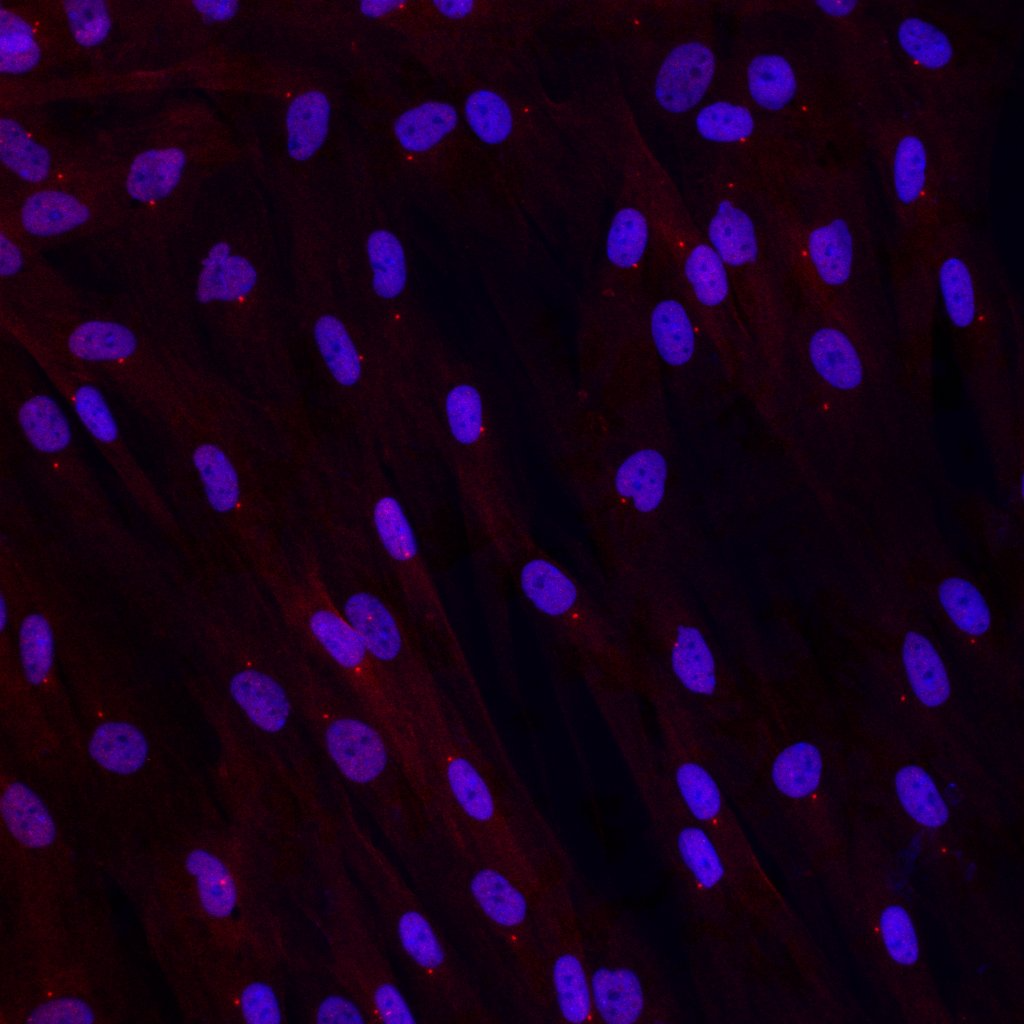

Supplement: Supplementary file 6 — Source Data for Expanded View [file EMMM-15-e16478-s001.zip › Expanded view source data/Figure EV5 (Microscopic images)/EV5A/IV.1(F2)(-_-).tiff]

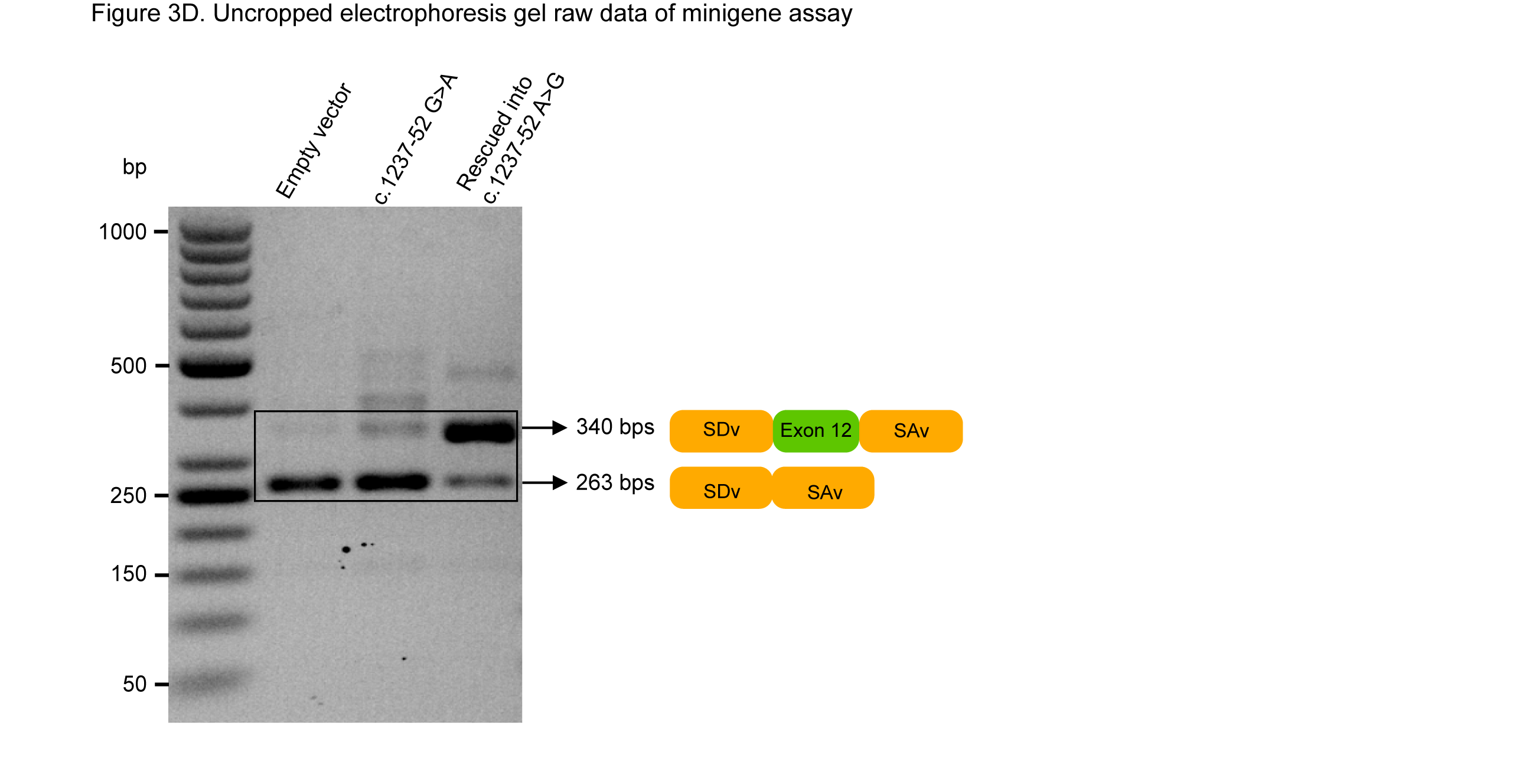

Supplement: Supplementary file 8 — Source Data for Figure 3 [file EMMM-15-e16478-s003.zip › Figure 3D (gel electrophoresis)/minigene RT-PCR gel.tif]

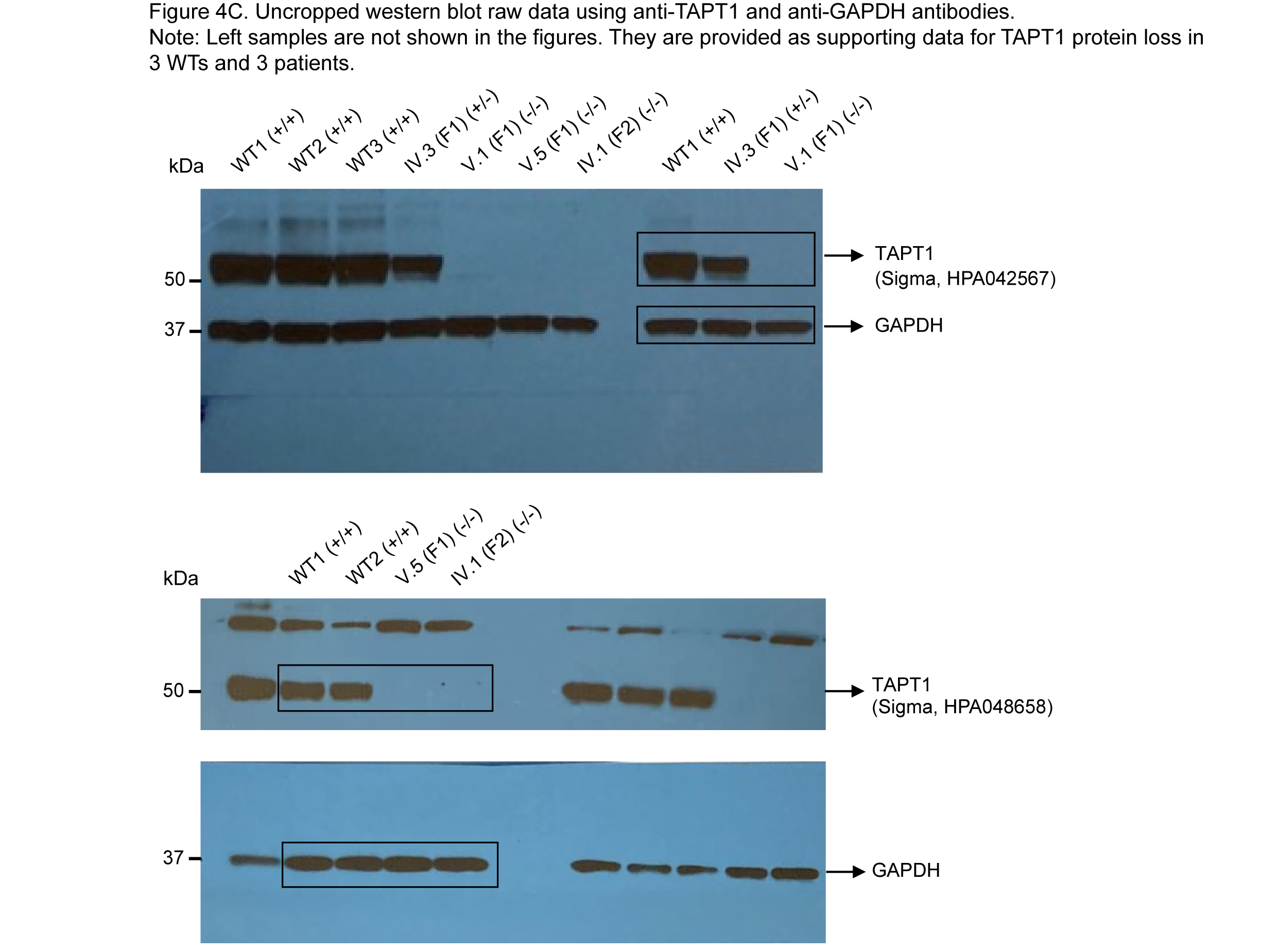

Supplement: Supplementary file 9 — Source Data for Figure 4 [file EMMM-15-e16478-s008.zip › Figure 4C (blot)/TAPT1 western blot.tif]

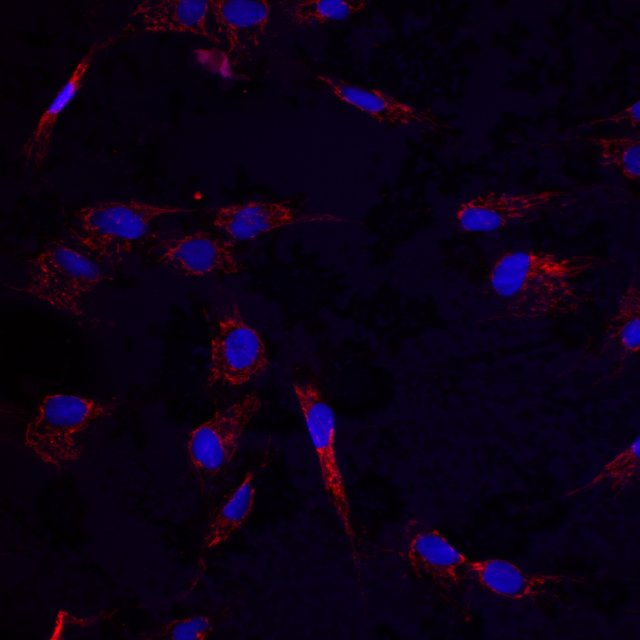

Supplement: Supplementary file 10 — Source Data for Figure 5 [file EMMM-15-e16478-s005.zip › Figure 5C (Microscopy images)/2. CANX/V.5(F1)(-_-).tiff]

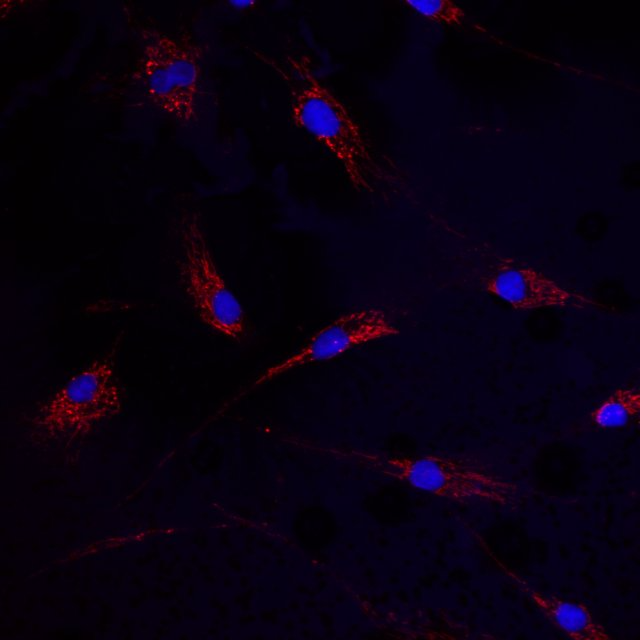

Supplement: Supplementary file 10 — Source Data for Figure 5 [file EMMM-15-e16478-s005.zip › Figure 5C (Microscopy images)/2. CANX/WT1(+_+).tiff]

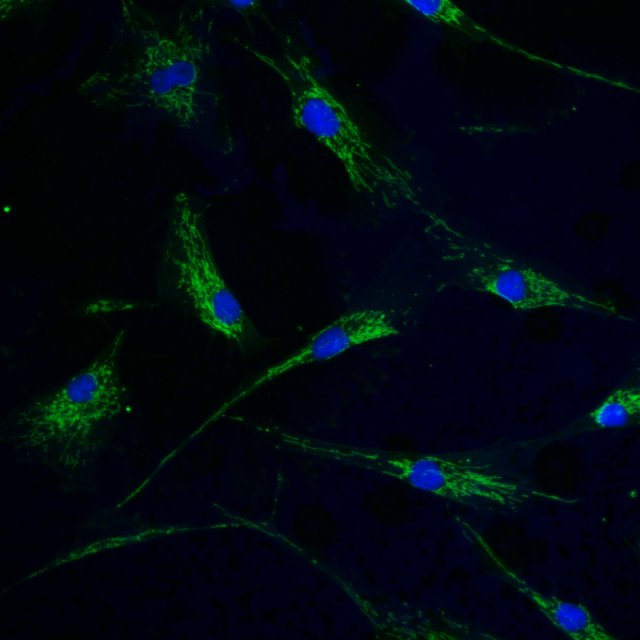

Supplement: Supplementary file 10 — Source Data for Figure 5 [file EMMM-15-e16478-s005.zip › Figure 5C (Microscopy images)/1. TOM20/WT1 (+_+).tiff]

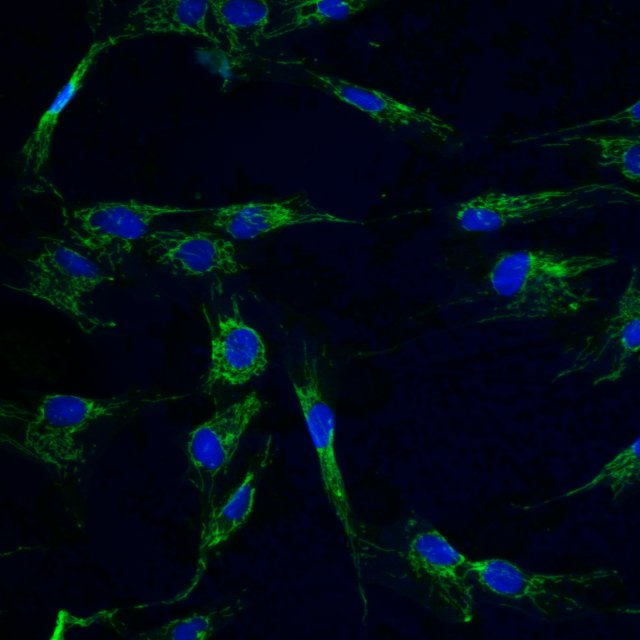

Supplement: Supplementary file 10 — Source Data for Figure 5 [file EMMM-15-e16478-s005.zip › Figure 5C (Microscopy images)/1. TOM20/V.5 (F1)(-_-).tiff]

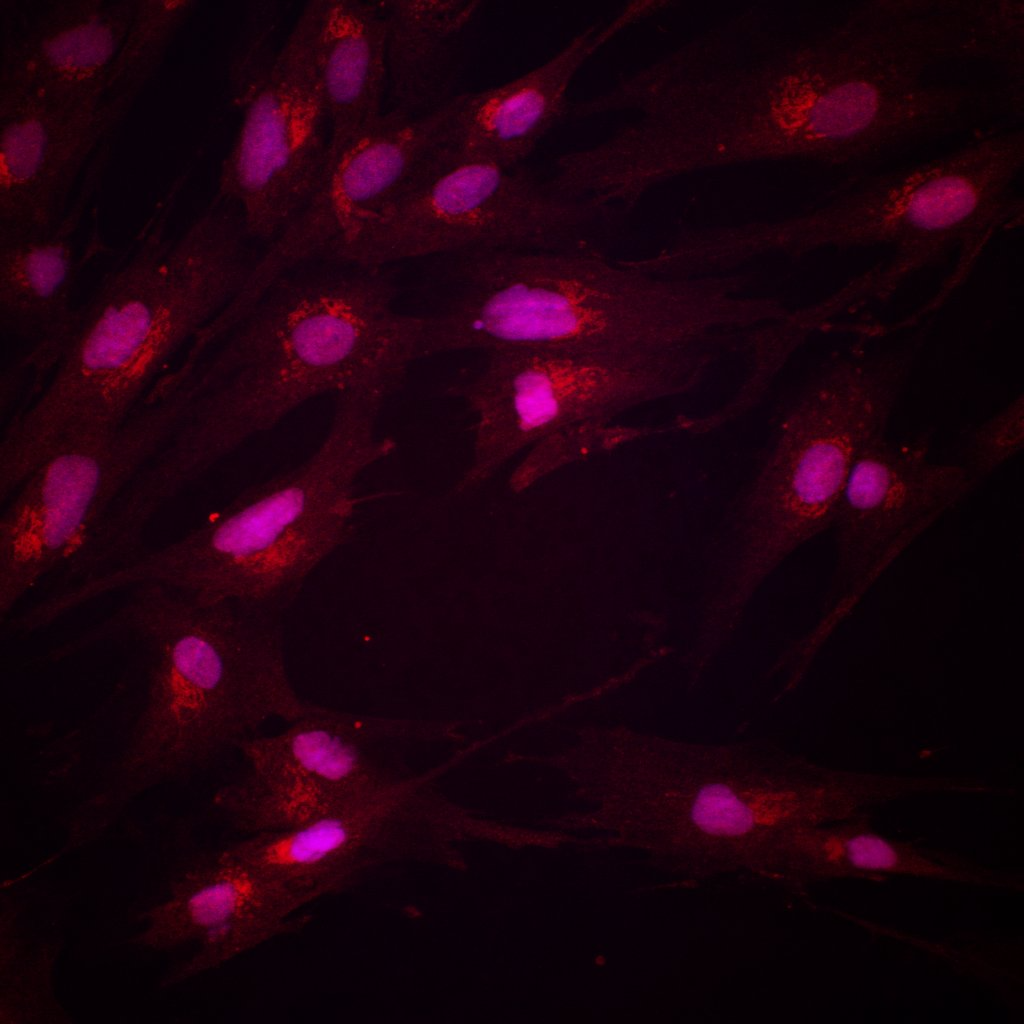

Supplement: Supplementary file 10 — Source Data for Figure 5 [file EMMM-15-e16478-s005.zip › Figure 5C (Microscopy images)/3. GLG1/V.5(F1)(-_-).tiff]

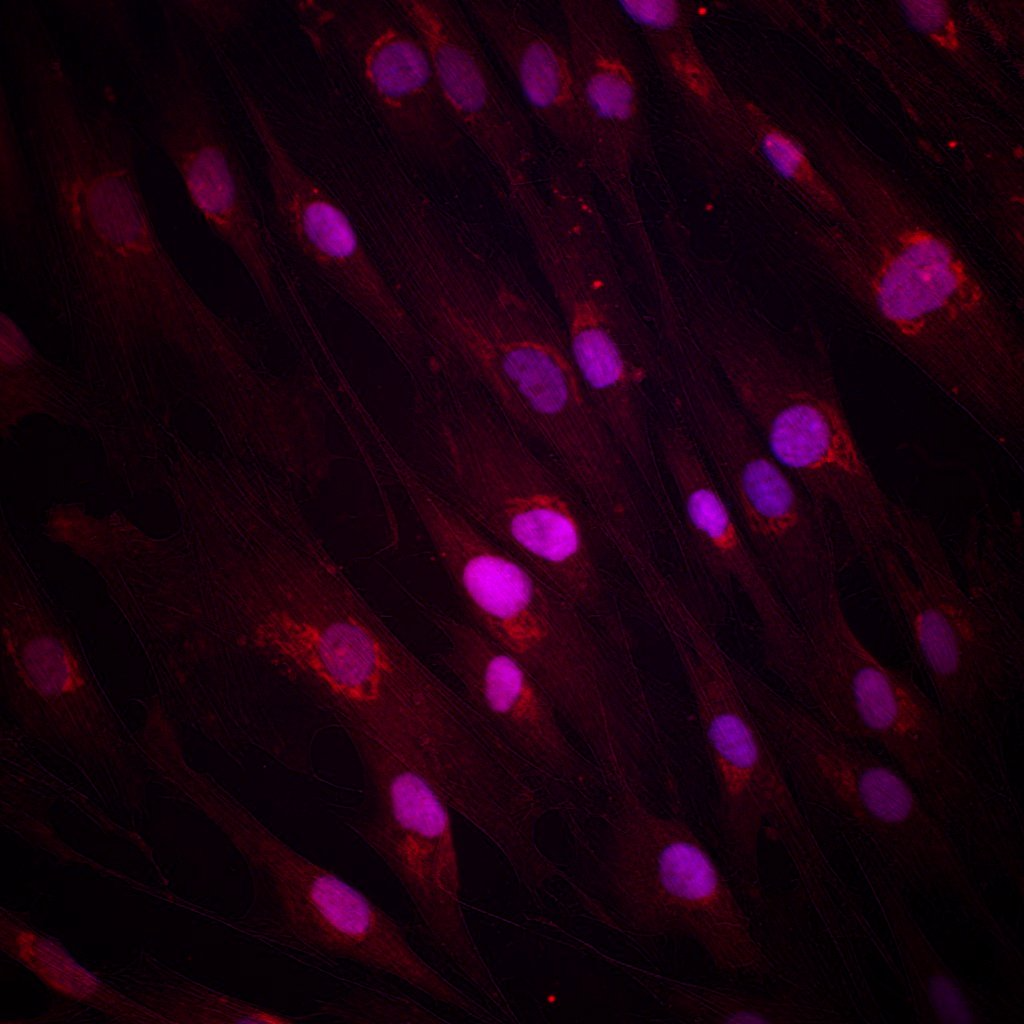

Supplement: Supplementary file 10 — Source Data for Figure 5 [file EMMM-15-e16478-s005.zip › Figure 5C (Microscopy images)/3. GLG1/WT1(+_+) replicate data.tiff]

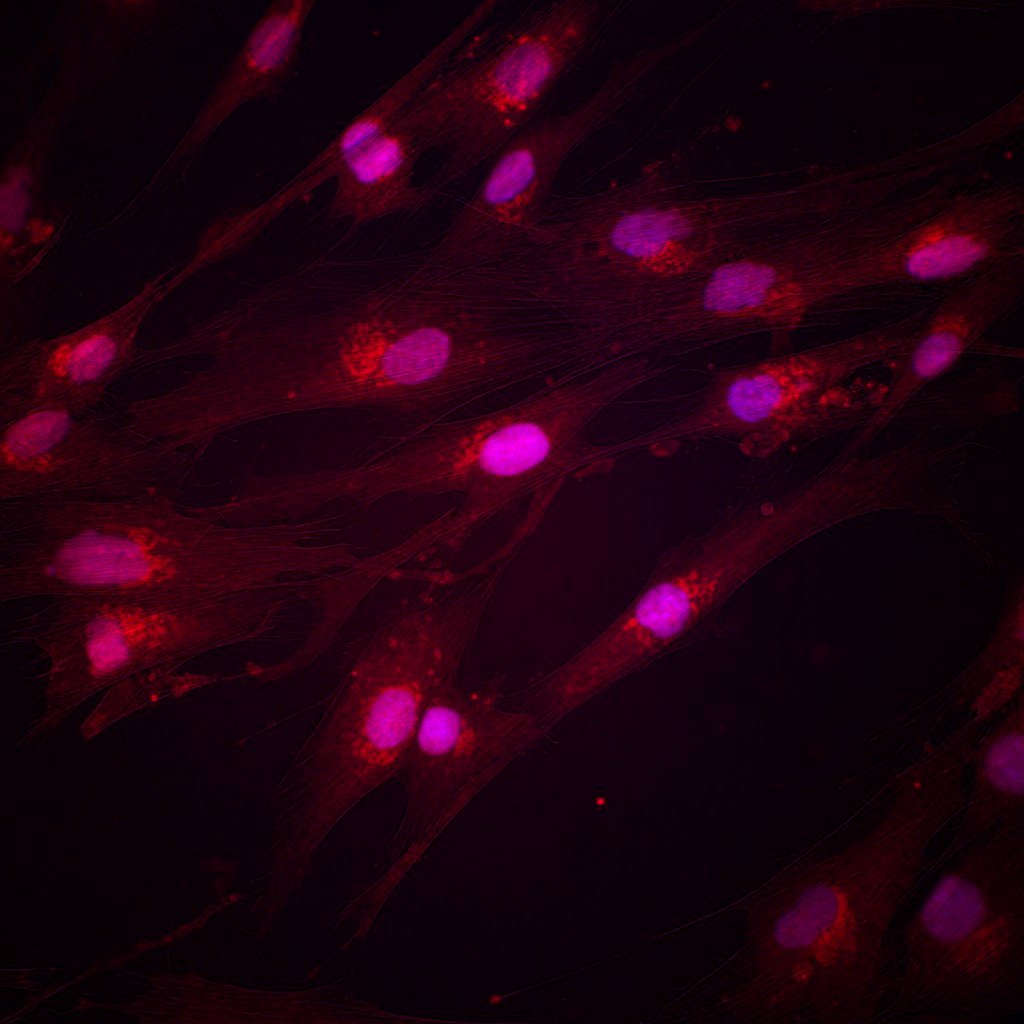

Supplement: Supplementary file 10 — Source Data for Figure 5 [file EMMM-15-e16478-s005.zip › Figure 5C (Microscopy images)/3. GLG1/V.5(F1)(-_-) replicate data.tiff]

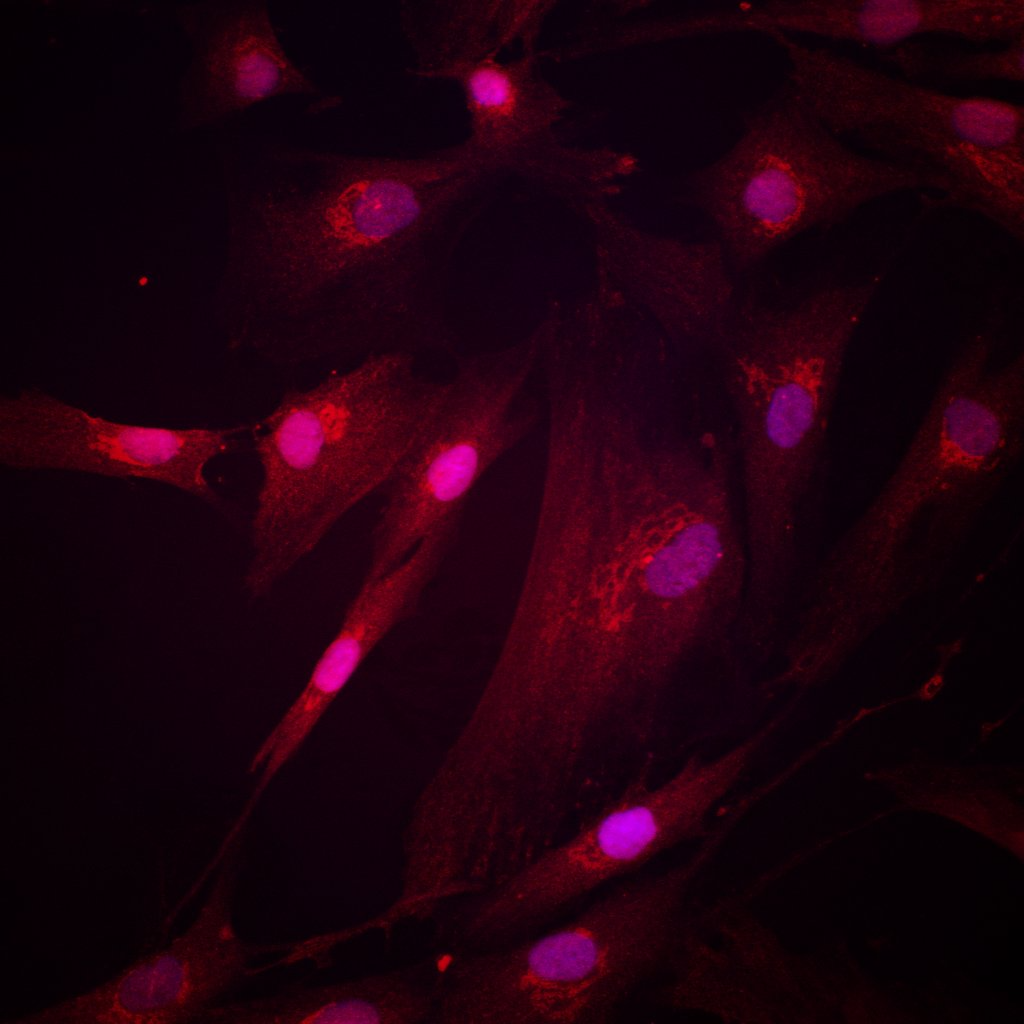

Supplement: Supplementary file 10 — Source Data for Figure 5 [file EMMM-15-e16478-s005.zip › Figure 5C (Microscopy images)/3. GLG1/WT1(+_+).tiff]

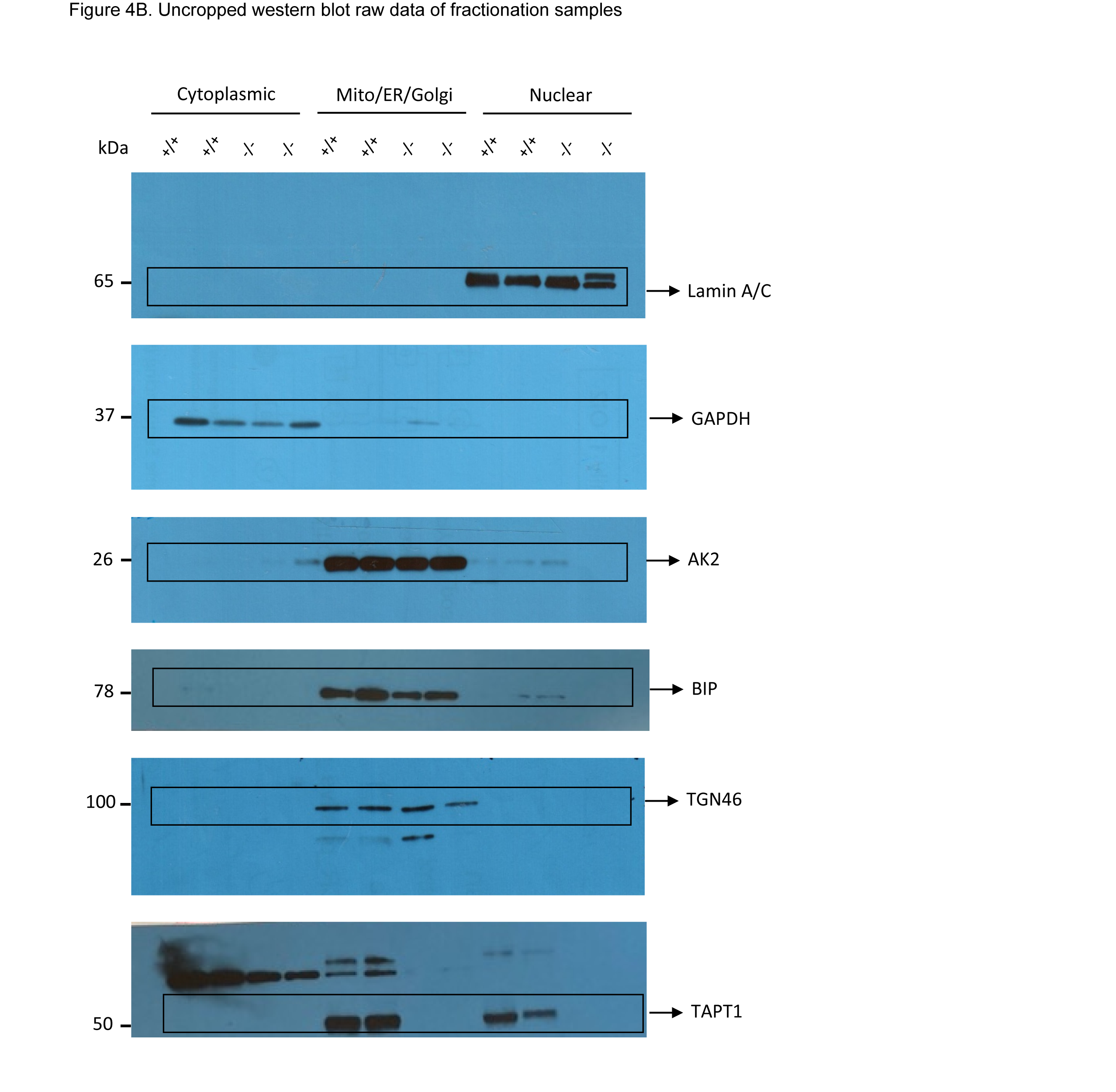

Supplement: Supplementary file 10 — Source Data for Figure 5 [file EMMM-15-e16478-s005.zip › Figure 5B (Blot)/Fractionation western blot.tif]
