# Supplementary material for: A progeroid syndrome caused by a deep intronic variant in TAPT1 is revealed by RNA/SI‐NET sequencing
Source: EMBO Mol Med. 2023 Jan 18;15(2):e16478. doi: 10.15252/emmm.202216478 (PMC9906387; doi:10.15252/emmm.202216478)
Supplement: Supplementary file 7 — PDF+ [file EMMM-15-e16478-s006.pdf]

Article

SOURCE DATA TRANSPARENT PROCESS OPEN ACCESS

EMBO Molecular Medicine

A progeroid syndrome caused by a deep intronic variant in *TAPT1* is revealed by RNA/SI-NET sequencing

Nasrinsadat Nabavizadeh[1,2,3,†], Annkatrin Bressin[4,†], Mohammad Shboul[5], Ricardo Moreno Traspas[1], Poh Hui Chia[1], Carine Bonnard[6], Emmanuelle Szenker-Ravi[1], Burak Sarıbaş[1,3], Emmanuel Beillard[7], Umut Altunoglu[3], Zohreh Hojati[2], Scott Drutman[8], Susanne Freier[4], Mohammad El-Khateeb[9], Rajaa Fathallah[9], Jean-Laurent Casanova[8,10,11,12,13], Wesam Soror[9], Alaa Arafat[9], Nathalie Escande-Beillard[3,14], Andreas Mayer[4,*] & Bruno Reversade[1,3,14,15,16,**]

Abstract

Exome sequencing has introduced a paradigm shift for the identification of germline variations responsible for Mendelian diseases. However, non-coding regions, which make up 98% of the genome, cannot be captured. The lack of functional annotation for intronic and intergenic variants makes RNA-seq a powerful companion diagnostic. Here, we illustrate this point by identifying six patients with a recessive Osteogenesis Imperfecta (OI) and neonatal progeria syndrome. By integrating homozygosity mapping and RNA-seq, we delineated a deep intronic *TAPT1* mutation (c.1237-52 G>A) that segregated with the disease. Using SI-NET-seq, we document that *TAPT1*'s nascent transcription was not affected in patients' fibroblasts, indicating instead that this variant leads to an alteration of pre-mRNA processing. Predicted to serve as an alternative splicing branchpoint, this mutation enhances *TAPT1* exon 12 skipping, creating a protein-null allele. Additionally, our study reveals dysregulation of pathways involved in collagen and extracellular matrix biology in disease-relevant cells. Overall, our work highlights the power of transcriptomic approaches in deciphering the repercussions of non-coding variants, as well as in illuminating the molecular mechanisms of human diseases.

Keywords non-coding variant; Osteogenesis Imperfecta; RNA-seq; SI-NET-seq; *TAPT1*
Subject Categories Development; Genetics, Gene Therapy & Genetic Disease; Musculoskeletal System
DOI 10.15252/emmm.202216478 | Received 21 June 2022 | Revised 13 December 2022 | Accepted 14 December 2022 | Published online 18 January 2023
EMBO Mol Med (2023) 15: e16478

Introduction

Whole exome sequencing (WES) targets less than 2% of our genome, whereas the majority of non-coding sequences are still understudied. These crucial sequences for gene regulation are to a large extent transcribed and form a significant portion of our genome which are also susceptible to harbor variants responsible for human diseases (Djebali *et al*, 2012; Khan *et al*, 2017; Chen

1 Laboratory of Human Genetics & Therapeutics, Genome Institute of Singapore, A*STAR, Singapore City, Singapore
2 Division of Genetics, Department of Cell and Molecular Biology & Microbiology, Faculty of Biological Science and Technology, University of Isfahan, Isfahan, Iran
3 Medical Genetics Department, Koç University School of Medicine, Istanbul, Turkey
4 Max Planck Institute for Molecular Genetics, Berlin, Germany
5 Department of Medical Laboratory Sciences, Jordan University of Science and Technology, Irbid, Jordan
6 Model Development, A*STAR Skin Research Labs (A*SRL), Singapore City, Singapore
7 Department of Biopathology, Centre Léon Bérard, Lyon, France
8 St. Giles Laboratory of Human Genetics of Infectious Diseases, Rockefeller Branch, Rockefeller University, New York, NY, USA
9 National Center for Diabetes, Endocrinology and Genetics, Amman, Jordan
10 Laboratory of Human Genetics of Infectious Diseases, Necker Branch, INSERM U1163, Necker Hospital for Sick Children, Paris, France
11 Imagine Institute, University of Paris, Paris, France
12 Howard Hughes Medical Institute, New York, NY, USA
13 Pediatric Hematology and Immunology Unit, Necker Hospital for Sick Children, Paris, France
14 Institute of Molecular and Cell Biology, A*STAR, Singapore City, Singapore
15 Department of Paediatrics, National University of Singapore, Singapore City, Singapore
16 Smart-Health Initiative, BESE, KAUST, Thuwal, Kingdom of Saudi Arabia
*Corresponding author. Tel: +49 30 8413 1264; E-mail: mayer@molgen.mpg.de
**Corresponding author. Tel: +966 545352602; E-mail: bruno@reversade.com
†These authors contributed equally to this work as first authors

© 2023 The Authors. Published under the terms of the CC BY 4.0 license.

et al, 2019; Jamshidi et al, 2019). Indeed, from the more than 4,000 Mendelian phenotypes reported to date, of which approximately 50% still lack the identification of the underlying genetic cause (Chong et al, 2015). This speaks to the necessity to further explore non-coding sequences. Whole-genome sequencing (WGS) provides a more comprehensive method to cover the full genome, however, a key challenge to its implementation is the prioritization of the vast amount of non-coding variants identified. This barrier to interpretation is in part driven by the lack of annotated information in intronic and intergenic regions which together comprise up to 98% of our genome. RNA-sequencing (RNA-seq) has proven to be a powerful complementary approach to overcome these hurdles by revealing the functional impact of the genetic variants at the transcriptome level. The use of RNA-seq in conjunction with WGS permits cross-referencing of endogenous RNA levels and splicing events to help prioritize disease-causing mutations at the DNA level (Cummings et al, 2017; Evrony et al, 2017; Kremer et al, 2017).

Here we report the study of six affected children from two consanguineous Jordanian families that presented with a congenital syndrome consisting of osteogenesis imperfecta (OI), severe developmental delay and neonatal progeria. By combining homozygosity mapping, RNA-seq and targeted Sanger sequencing, we identified an intronic homozygous variant (c.1237-52 G>A) in *TAPT1* (MIM612758) which entirely segregated with the disease. Using patient-derived fibroblasts, our downstream characterization methods including an *in vitro* splicing assay showed how this private non-coding mutation aggravates skipping of exon 12 leading to a TAPT1 protein-null allele.

TAPT1 which codes for a predicted transmembrane protein is involved in ER/Golgi pathways, human Cytomegalovirus (HCMV) infection and primary ciliogenesis (Baldwin et al, 1996, 2000; Jonikas et al, 2009; Symoens et al, 2015; LaMonte et al, 2016, 2020; Zhang et al, 2017a, 2017b). Our functional studies using patient-derived TAPT1-knockout cells could not detect patent anomalies in the pathways previously linked to TAPT1, indicating that its precise molecular function has yet to be ascertained. Notwithstanding, our RNA-seq and SI-NET-seq analyses revealed a role for TAPT1 in collagen and ECM biology, which is consistent with the clinical presentation of our patients. Overall, our study highlights the capacity of applying robust transcriptomic approaches to prioritize disease-causing genes and understand the underlying pathogenesis of Mendelian disease.

Results

A severe recessive progeroid syndrome with osteogenesis imperfecta

We investigated six severely-affected children from two consanguineous Jordanian families (Fig 1A and B) manifesting growth retardation, short stature, multiple bone deformities, lipodystrophy and neonatal progeria. The patients from both families had various craniofacial abnormalities including prominent forehead, plagiocephaly, depressed nasal bridge, nasal septum deviation, low set ears, ear deformities, micrognathia, and occult cleft palate (Figs 1C–E and EV1). The patients also suffered from microphthalmia, cataract, and bilateral esotropia. They had translucent, wrinkled skin with patent acrogeria and sparse hair with premature depigmentation (Figs 1C–E and EV1). They also displayed pectus excavatum and brachydactyly of both hands and feet (Figs 1C–E and EV1). X-ray and MRI (magnetic resonance imaging) tests were performed for patient V.1 (F1). X-ray images showed extensive deformity of the bones, bone dysplasia with bowing, and evidence of previous multiple fractures (Fig 1F). This proband had spared joints, a flattened epiphysis of the humeral bone, irregular growth of arm bones resulting in small deformed radius bone, and a bowed ulnar bone. She also presented a deformed clavicular bone with displacement of both claviculosternal and acromioclavicular joints, deformed shoulders, irregular development of the scapula, bilateral shallow acetabulum, abnormal contour of bilateral femoral head, and absent femoral diaphysis. X-rays also revealed severe calcification defects involving premature atherosclerotic vascular calcification, periarticular soft tissue calcification, and irregular calcification of carpal bones (Fig 1F). Brain abnormalities were also reported with cranial MRI showing defects in the white matter of the frontal and occipital lobes with pachygyria, possibly representing some form of leukodystrophy. The probands V.1 (F1) and V.5 (F1) died of severe respiratory infection and inflammation at the age of 10 and 4.5 years, respectively. The history of a similar disease was remarkable in this extended kindred. Two affected girls (IV.7 (F1) and V.13 (F1)) born to the mother's aunts who showed similar clinical manifestation and died of severe respiratory distress at the age of 5 years. Another case (V.12 (F1)) of 2 years of age is alive and manifests similar clinical features.

A deep intronic *TAPT1* variant segregates with the disease

Although the two families were reported to be unrelated, both originated from the Jordan valley. Assuming a founder mutation, we carried out homozygosity mapping for a total of 15 individuals including 3 affected patients (V.1 and V.5 (F1), IV.1 (F2)), 3 pairs of parents from both families, and 6 unaffected siblings from F1 (IV.4, IV.5, IV.6, V.2, V.3 and V.4). Homozygosity mapping confirmed distant relatedness between the 2 families with a minimal shared locus on chromosome 4 (4p16.1-p15.31) (hg19). The length of this Identical-by-Descent (IBD) locus was 8.4 Mb spanning a total of 39 candidate genes (Figs 2A and EV2A). We first performed whole-exome sequencing (WES) for V.1 (F1) and IV.1 (F2), but no compelling recessive mutations were found. To expand our search, we

Figure 1. Patients from two distantly related families present with a recessively inherited syndrome characterized by osteogenesis imperfecta and neonatal progeria.

A, B Pedigrees of two distantly related consanguineous families from Jordan, showing an autosomal recessive mode of inheritance of the disease. Black symbols and crossed symbols represent affected and deceased individuals, respectively.

C–E Pictures of investigated patients showing severe bone deformities and fractures, neonatal progeria, wrinkled skin, prominent forehead and pectus excavatum.

F Radiographs of affected V.1 (F1) showing several deficits in the bones including deformity, dysplasia, spared joints and evidence of previous fractures. Severe calcification defects can also be noticed, involving premature atherosclerotic vascular calcification, periarticular soft tissue calcification and irregular calcification of carpal bones.

 © 2023 The Authors

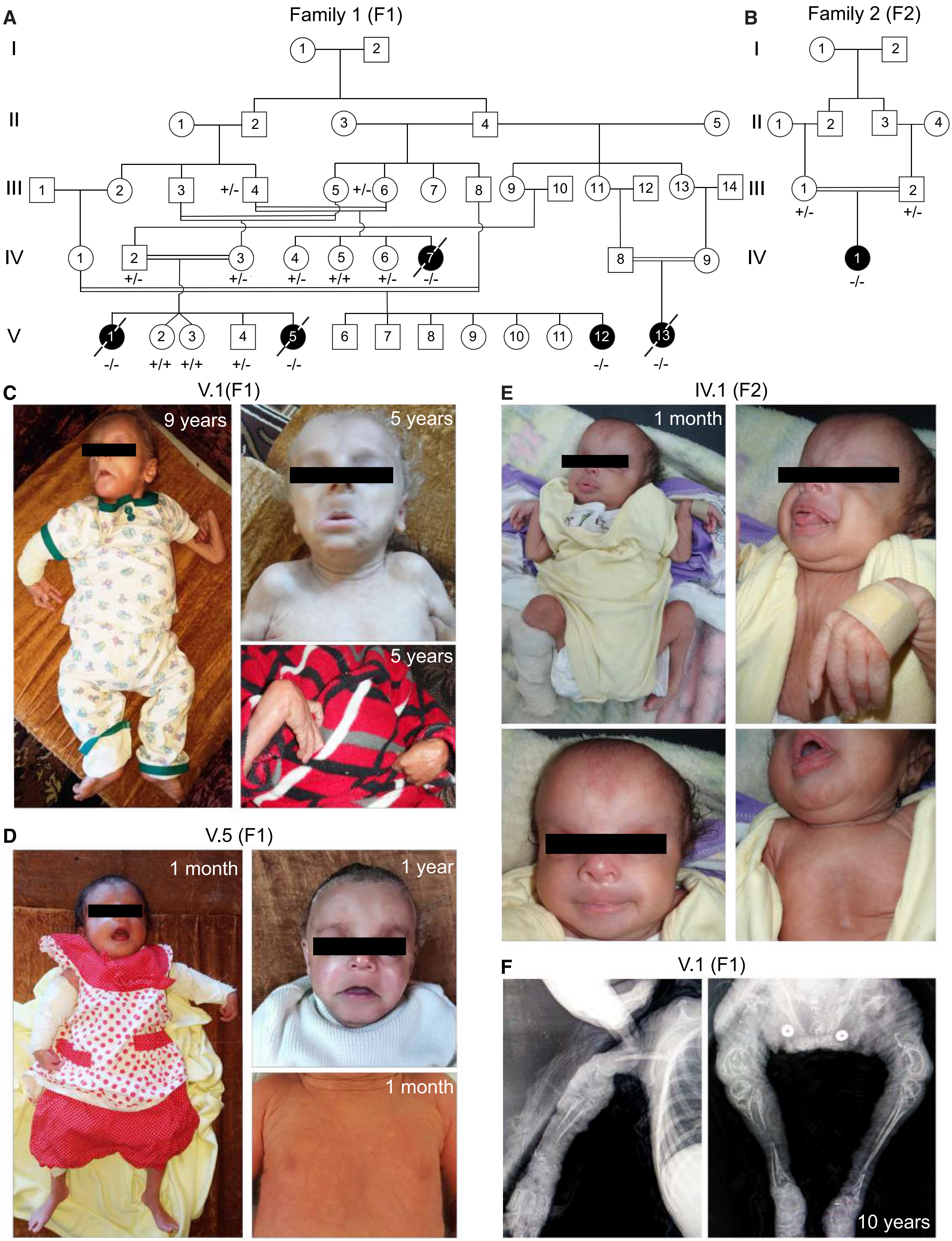
A
Family 1 (F1)
I
II
III
IV
V
-/-
+/+ +/+
+/-
B
Family 2 (F2)
C
V.1(F1)
9 years
5 years
5 years
D
V.5 (F1)
1 month
1 year
1 month
E
IV.1 (F2)
1 month
F
V.1 (F1)
10 years

Figure 1.

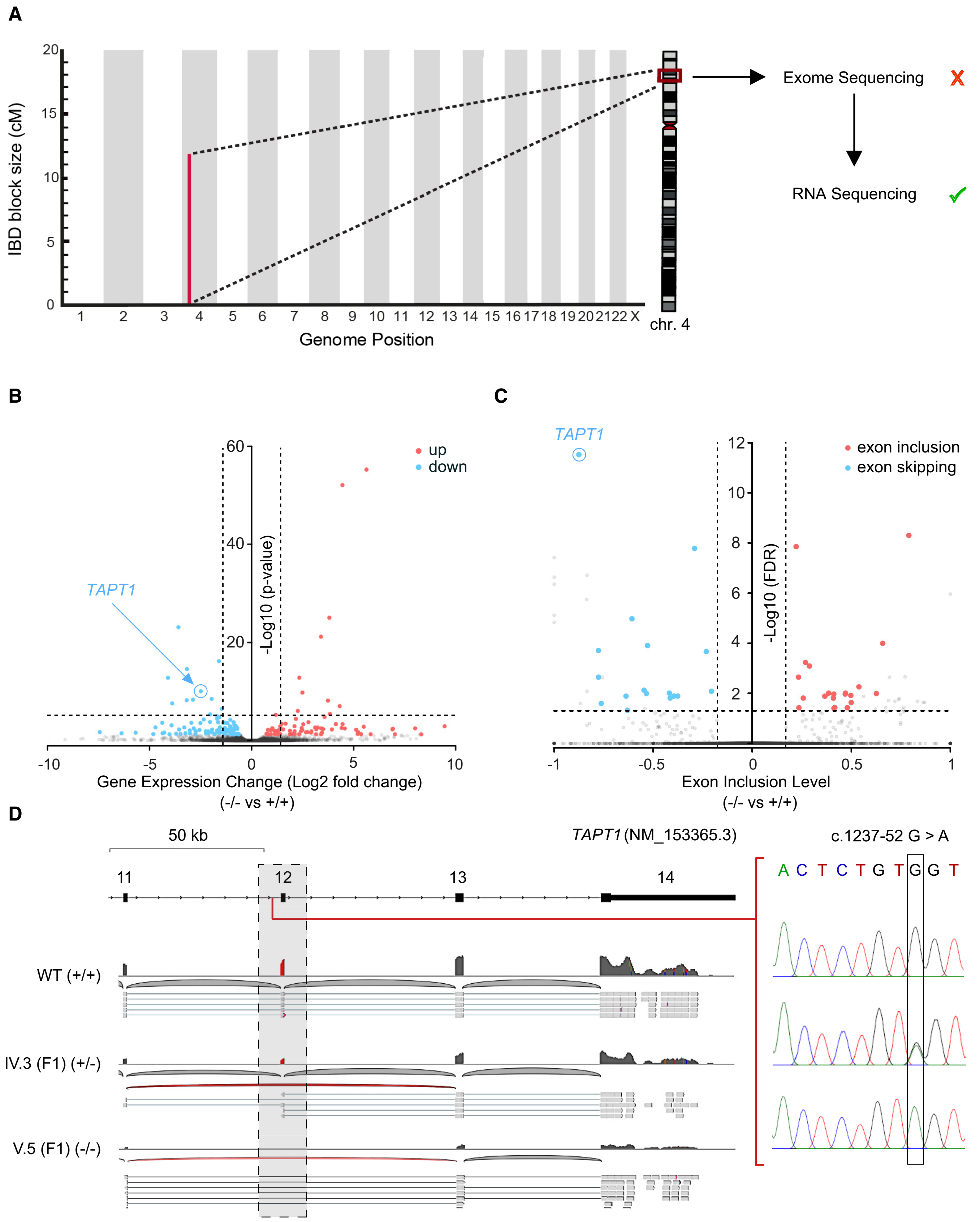

Figure 2.

© 2023 The Authors

Figure 2. Homozygosity mapping followed by RNA-seq uncovers a deep intronic recessive mutation in *TAPT1*.

A Schematic representation of the shared IBD region between both Jordanian families, located on Chromosome 4 (4p16.1–p15.31) with a size of ~ 12 cM. Although WES analysis did not reveal any mutations in the coding sequences located in the IBD region, RNA-seq analysis helped us to identify the disease causative gene from this locus.

B Volcano plot showing differentially expressed genes between WT (WT1 and WT2) and patient (V.1 (F1), V.5 (F1)) primary dermal fibroblasts. The vertical axis (*y*-axis) shows the −log10 *P*-value, whereas the horizontal axis (*x*-axis) displays the log2 fold change value. The red dots represent the upregulated transcripts; the blue dots represent the downregulated transcripts. A total of 172 genes were found significantly dysregulated. *TAPT1*, a gene located in the IBD region, appeared among the most significantly downregulated genes in the patients.

C Plot showing the alternative splicing analysis results from WT (WT1 and WT2) and patient (V.1 (F1), V.5 (F1)) primary dermal fibroblasts. The vertical axis (*y*-axis) shows the −log10 FDR (False Discovery Rate), whereas the horizontal axis (*x*-axis) represents the exon inclusion level (value ranging from −1 to 1). The red dots represent transcripts with exon inclusion events; the blue dots represent transcripts affected by exon skipping. A total of 63 aberrantly spliced genes were found in the patient cells, being *TAPT1* the most significant exon skipping event.

D (Left) Schematic representation showing the complete loss of exon 12 from *TAPT1* transcript in patient cells, as defined by our splicing analysis data. (Right) Chromatogram showing the novel intronic mutation (c.1237-52 G>A) we found entirely segregating with the disease in all available family members. For display purposes, results from the targeted Sanger sequencing in WT, IV.3 (F1) and V.5 (F1) individuals are shown. The mutation is present in heterozygosis in IV.3 (F1) (unaffected mother) and in homozygosis in V.5 (F1) (affected patient).

turned to an unbiased RNA-seq approach using primary cutaneous fibroblasts from 2 affected individuals (V.1 and V.5 (F1)), and 2 unrelated wild-type (WT1 and WT2) controls. Of the 39 candidate genes in the IBD region, our differential expression analysis data disclosed that *TAPT1* was the only significantly dysregulated transcript in the patient primary dermal fibroblasts (Log2 fold change = −2.5; Figs 2B and EV2B and D). Moreover, the alternative splicing analysis identified 63 genes with splicing defects in patient samples, *TAPT1* being the top significant transcript with an exon 12 skipping event (Figs 2C and D, and EV2C and D). Interestingly, homozygous mutations in *TAPT1* were previously reported as the genetic cause of complex osteochondrodysplasia (MIM616897) (Symoens *et al*, 2015). Although less severe, this disease bears strong clinical overlap with the syndrome reported in this study. The exon 12-skipping event prompted us to search for the presence of possible *TAPT1* intronic mutations. Targeted Sanger sequencing for this exon and its neighboring nucleotides (~ 300 bps) revealed a deep intronic single nucleotide polymorphism (NM_153365.3, c.1237-52 G>A) within intron 11 that entirely segregated with the disease in all available family members (Figs 2D and 3A). This variant was not reported in the Genome Aggregation Database (gnomAD). While there were similar variants described to occur in its vicinity, none of them were homozygous. Together, these findings indicate that the c.1237-52 G>A mutation within *TAPT1* intron 11 most likely caused the disease for the 6 affected children.

Exon 12 skipping targets *TAPT1* mutant transcripts for NMD, resulting in a protein-null allele

How does this deep intronic mutation in *TAPT1* lead to disease? To gain insights into the underlying disease-causing molecular mechanism, we applied a combined computational and experimental approach. We found that the private homozygous c.1237-52 G>A transition was predicted to serve as an alternative splicing branchpoint (Fig 3B), thereby resulting in the exclusion of *TAPT1* exon 12 (Fig 2D). To confirm the causality of this deep intronic mutation in exon 12 skipping, we adapted and used a minigene splicing assay (Westin *et al*, 2021; Iturrate *et al*, 2022; Rodriguez-Muñoz *et al*, 2022). Notably, transfection with the mutant construct showed that c.1237-52 G>A variant resulted mainly in a truncated splicing product (~ 260 bps), due to exon 12 skipping while the rescue construct without the mutation yielded a major full-length product containing exon 12 (Fig 3C). These *in vitro* findings were verified by RT–PCR on endogenous *TAPT1* transcripts using WT, heterozygous carrier and patient fibroblasts. In accordance with the results of the minigene assay, both *TAPT1* canonical and exon 12 skipped transcripts were detected in all tested samples. However, the private mutation enhanced exon 12 skipping (Fig EV3A).

As the complete loss of exon 12 creates a premature stop codon, we used orthogonal RT–qPCR validation tests to investigate whether the *TAPT1* mutant transcript was targeted for nonsense-mediated

Figure 3. *TAPT1* c.1237-52 G>A mutation triggers exon 12 skipping.

A Schematic representation of *TAPT1* and *TAPT1-AS1*, indicating the causative intronic mutation (c.1237-52 G>A). The transcription start sites and the direction of transcription are indicated by arrows. Scale bar represents 2 kb.

B Diagram showing the branchpoint scores for the target c.1237-52 position and flanking nucleotides in *TAPT1* intron 11 in both WT (+/+) and patient cells (−/−), as obtained from the RNABPS (Nazari *et al*, 2018), LaBranchoR (Paggi & Bejerano, 2018) and BPP (Zhang *et al*, 2017a) softwares. High branchpoint scores were predicted for the G>A transition in the patient cells using the RNABPS and LaBranchoR methods. The *x*-axis represents the nucleotide distance to the 3′ splice site (3′SS).

C Schematic illustration of minigene constructs and RT–PCR analysis of splicing products. The pSPL3 vector contains SDv and SAv exons (gray boxes) and functional intron (black line) in its backbone. SDv: splice donor vector; SAv: splice acceptor vector. *TAPT1* c.1237-52 G>A mutant fragments containing 200 bps of intron 11, exon 12 and 500 bps of intron 12 (green) were cloned into the *EcoRI* and *BamH1* cloning sites (pink) of the pSPL3 vector. Using site directed mutagenesis, the *TAPT1* c.1237-52 G>A mutant construct was rescued into c.1237-52 A>G (Purple arrow: c.1237-52 G>A; green arrow: rescued into c.1237-52 A>G). Green and red lines show canonical and internal/aberrant splicing, respectively. Two *TAPT1* minigene constructs and an empty pSPL3 vector were transfected into HEK293T cells for 24 h. Following RNA extraction and cDNA synthesis, RT–PCR was done using vector specific primers (F: SD6 forward; R: SA2 reverse). The 263 bp PCR product in the empty vector showed internal splicing between SDv and SA2 exons. In c.1237-52 G>A mutant minigene construct, the majority of splicing products had a size of 263 bp due to the aberrant exon 12 skipping while in the rescued construct, most of the transcripts had the expected size of 340 bps. Direct Sanger sequencing confirmed the identity of the normal and exon-12 skipped products.

Source data are available online for this figure.

© 2023 The Authors

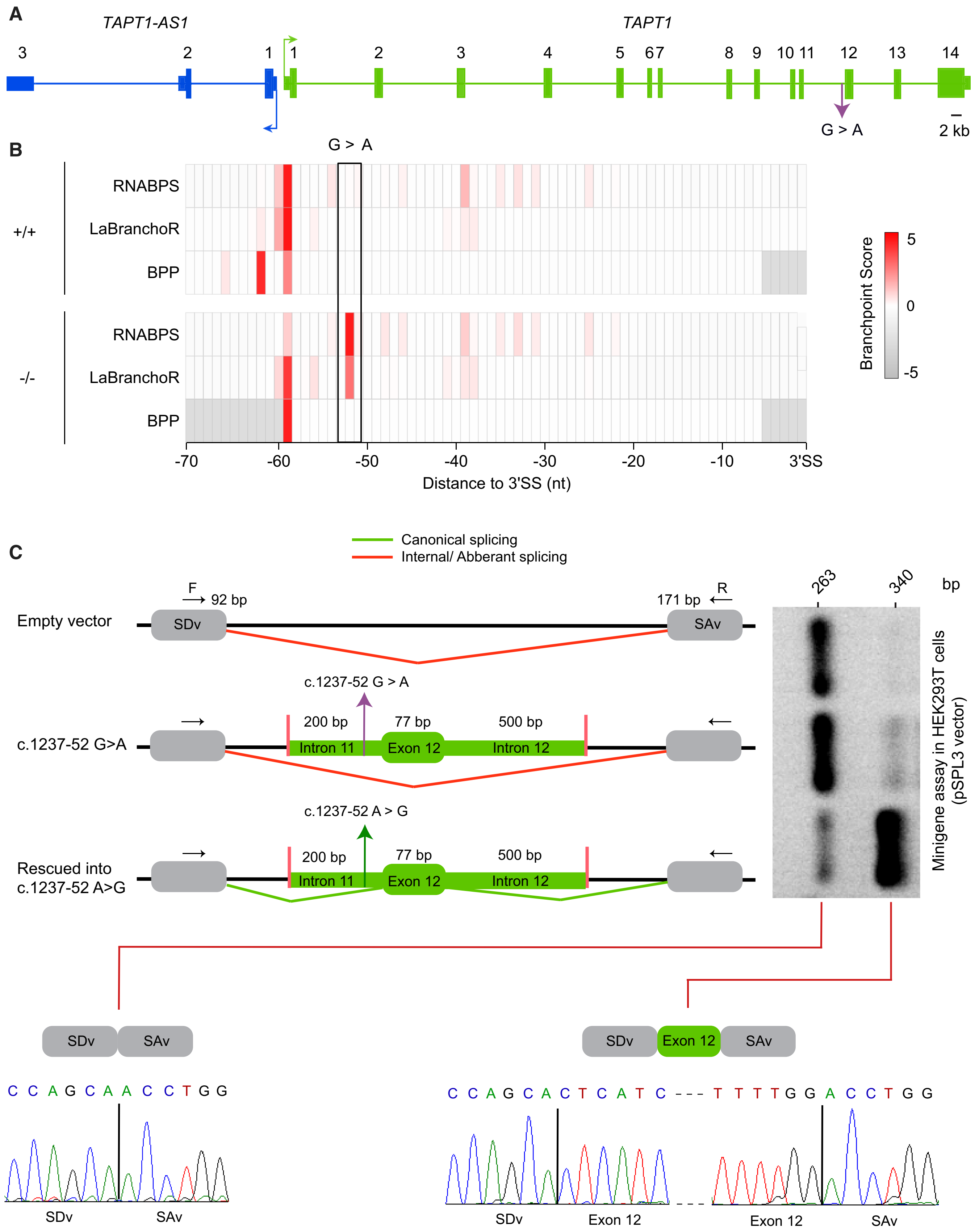

Figure 3.

© 2023 The Authors

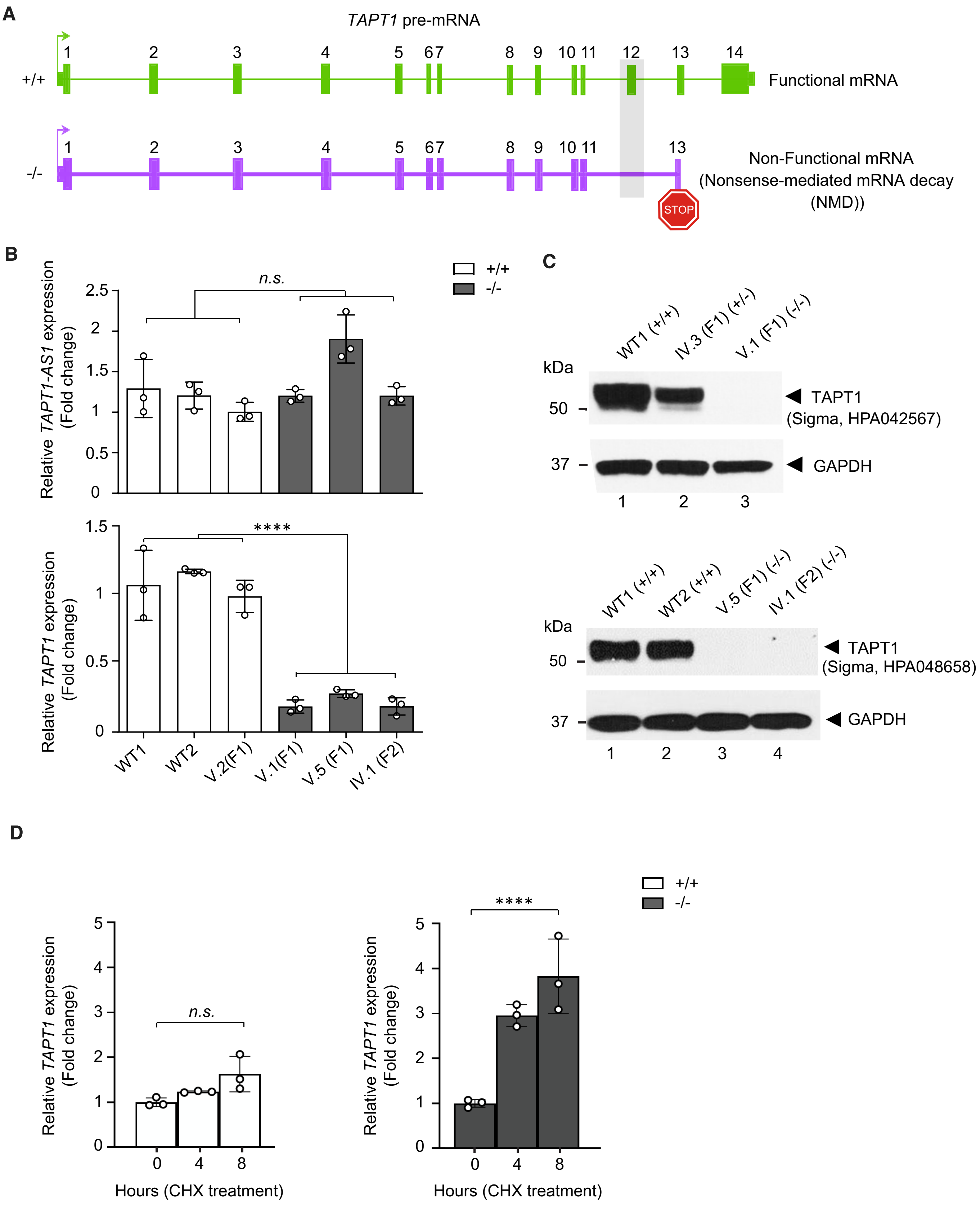
A
TAPT1 pre-mRNA
+/+
1 2 3 4 5 6 7 8 9 10 11 12 13 14
Functional mRNA
-/-
1 2 3 4 5 6 7 8 9 10 11 13
STOP
Non-Functional mRNA
(Nonsense-mediated mRNA decay
(NMD))
B
Relative TAPT1-AS1 expression
(Fold change)
2.5
2
1.5
1
0.5
0
n.s.
+/+
-/-
Relative TAPT1 expression
(Fold change)
1.5
1
0.5
0
****
WT1
WT2
V.2(F1)
V.1(F1)
V.5 (F1)
IV.1 (F2)
C
kDa
WT1 (+/+)
IV.3 (F1) (+/-)
V.1 (F1) (-/-)
50
37
TAPT1
(Sigma, HPA042567)
GAPDH
1 2 3
kDa
WT1 (+/+)
WT2 (+/+)
V.5 (F1) (-/-)
IV.1 (F2) (-/-)
50
37
TAPT1
(Sigma, HPA048658)
GAPDH
1 2 3 4
D
+/+
-/-
Relative TAPT1 expression
(Fold change)
5
4
3
2
1
0
n.s.
0 4 8
Hours (CHX treatment)
****
0 4 8
Hours (CHX treatment)

Figure 4.

Figure 4. Exon-12 skipped transcripts undergo NMD to create a protein-null allele.

A Schematic representation showing that the complete loss of exon 12 in *TAPT1* results in a premature stop codon, which targets the transcript for nonsense-mediated mRNA decay.

B qPCR results using specific primers for *TAPT1* and *TAPT1-AS1* in 3 WT (WT1, WT2, V.2 (F1)) and 3 affected (V.1 (F1), V.5 (F1), IV.1 (F2)) primary fibroblasts. *TAPT1* mRNA is significantly reduced in all patients compared with WTs, whereas *TAPT1-AS1* transcript levels are unaffected. Fold change relative to V.2 (F1) is plotted as mean ± SD. Asterisks indicate conventional statistical significance (Student's *t*-test; n.s. *P*-value > 0.05, ****P-value < 0.0001).

C Western blot analysis of endogenous TAPT1 protein (~ 60 kDa) using whole protein extracts from primary dermal fibroblasts from WT (WT1 and WT2), heterozygous (IV.3 (F1)) and homozygous (V.1 (F1), V.5 (F1) and IV.1 (F2)) individuals and two different commercial antibodies (top: Sigma, HPA042567; bottom: Sigma, HPA048658). Results show a complete absence of TAPT1 protein in patient samples. GAPDH was used as a loading control.

D qPCR analysis of *TAPT1* expression in 3 WT (WT1, WT2, WT3) and 3 affected (V.1 (F1), V.5 (F1), IV.1 (F2)) primary fibroblasts treated with cycloheximide (CHX). CHX was used to block nonsense mediated decay (NMD). Our results showed a time dependent increase in the level of *TAPT1* transcripts in all 3 patient cells while *TAPT1* RNA level remained constant in the WT cells. For each graph, fold change relative to non-treated condition is plotted as mean ± SD. Asterisks indicate conventional statistical significance (Student's *t*-test; n.s. *P*-value > 0.05, ****P-value < 0.0001).

Source data are available online for this figure.

decay (NMD; Fig 4A). Our data confirmed the statistically significant reduction of endogenous *TAPT1* mRNA levels in three of the patients' primary fibroblasts compared with WT individuals (Fig 4B). We next examined the effect of the identified deep intronic *TAPT1* mutation on the expression of its encoded protein. We employed two different commercial antibodies to detect endogenous TAPT1 in protein extracts from primary fibroblast cultures from two distinct patients and two WT individuals. Western blotting with both antibodies showed a complete loss of endogenous TAPT1 protein in all patient cells carrying the c.1237-52 G>A mutation in homozygosity (Fig 4C). Notably, the fibroblasts from the mother IV.3 (F1) showed intermediate TAPT1 protein levels, which were also consistent with her heterozygous genotype (Fig 4C).

To clarify whether NMD was responsible for the degradation of aberrant transcripts in patient cells, we treated the patient and WT fibroblast with a potent NMD inhibitor cycloheximide (CHX) and assessed the rescue of NMD-sensitive transcripts by qPCR. Interestingly, the level of *TAPT1* transcripts was significantly increased in the patient cells upon CHX treatment, indicating that the mutation induced NMD (Fig 4D). A mild but not significant increase was also observed in the treated WT cells due to low levels of *TAPT1* exon-12 skipped transcripts. Moreover, the c.1237-52 G>A mutation had no impact on the stability of the *TAPT1* RNA (Fig EV4A). Taken together, these findings indicate that the novel mutant variant reported in this study behaves as a protein-null allele by creating an aberrant mis-spliced *TAPT1* transcript, which undergoes degradation before being translated.

The *TAPT1* antisense transcript is inconsequential for *TAPT1* gene expression

TAPT1 is situated head-to-head with its sequence-related antisense gene *TAPT1-AS1* (Fig 3A), which encodes a long non-coding RNA. Such upstream antisense transcripts can play a critical role in the regulation of gene expression (Faghihi & Wahlestedt, 2009; Seila *et al*, 2009; Pelechano & Steinmetz, 2013; Lloret-Llinares *et al*, 2016), in particular towards their associated protein-coding genes (Faghihi *et al*, 2008; Yu *et al*, 2008). Here, because of the manifest physical proximity of *TAPT1* and *TAPT1-AS1*, it is likely that both genes are expressed in a coordinated manner through shared regulatory elements as previously described for the majority of long non-coding RNA:mRNA gene pairs (Sigova *et al*, 2013). As such, we examined whether the downregulation of *TAPT1* may also alter the expression levels of *TAPT1-AS1*. qPCR data showed no overt changes in *TAPT1-AS1* levels in patients' fibroblasts relative to control cells (Fig 4B), thereby indicating that *TAPT1* downregulation does not affect the expression of its neighbor antisense transcript. To further investigate the possible regulatory function of *TAPT1-AS1* on its target gene, we knocked down the endogenous transcript in WT and TAPT1 mutant fibroblasts by transient transfection of two different *TAPT1-AS1* GapmeRs. As evidenced by our qPCR results, no significant alterations were detected in *TAPT1* mRNA levels (Fig EV4B), although both GapmeRs achieved the near complete depletion of *TAPT1-AS1* transcripts in both control and patient cells. In addition, TAPT1 protein expression was also found unaffected in a *TAPT1-AS1* knocked down context (Fig EV4C). These results argued against the potential regulatory role of *TAPT1-AS1* on *TAPT1* expression, and hence excluded the possibility that this antisense transcript could have an impact on the pathogenesis of the disease observed in our patients.

TAPT1 is enriched in the ER/Golgi and is dispensable for HCMV gH infection

TAPT1 codes for a protein termed Transmembrane Anterior Posterior Transformation 1, with 5 membrane-spanning helices (Fig 5A). Its cellular localization has been reported to be either in the endoplasmic reticulum (ER) or at the centrosome (Jonikas *et al*, 2009; Symoens *et al*, 2015; Zhang *et al*, 2017b). In order to gain further insights in its cellular localization, we performed immunofluorescence (IF) staining with two commercial TAPT1 antibodies in WT and mutant primary dermal fibroblasts. Each antibody yielded different staining patterns especially for centrosome and the cytoplasm, which were identical between control and TAPT1 knockout cells (Fig EV4D and E). This result clearly indicated that these antibodies were not suitable for IF purposes. Since we validated the use of the same antibodies for western blotting, we opted to conduct subcellular fractionation on patient and WT fibroblasts as an alternative strategy to examine its subcellular localization. Endogenous TAPT1 was enriched in the Mito/ER/Golgi fractions and, to a lesser extent, in the nuclear fractions (Fig 5B). These data were consistent with the previous reports on EMP65, the homologous TAPT1 protein in yeast (Jonikas *et al*, 2009; Zhang *et al*, 2017b), and pfcarl, the homologous TAPT1 protein in plasmodium (LaMonte *et al*, 2020), showing a preferential localization in the ER/Golgi apparatus. Additional evidence of TAPT1 localization in the

© 2023 The Authors

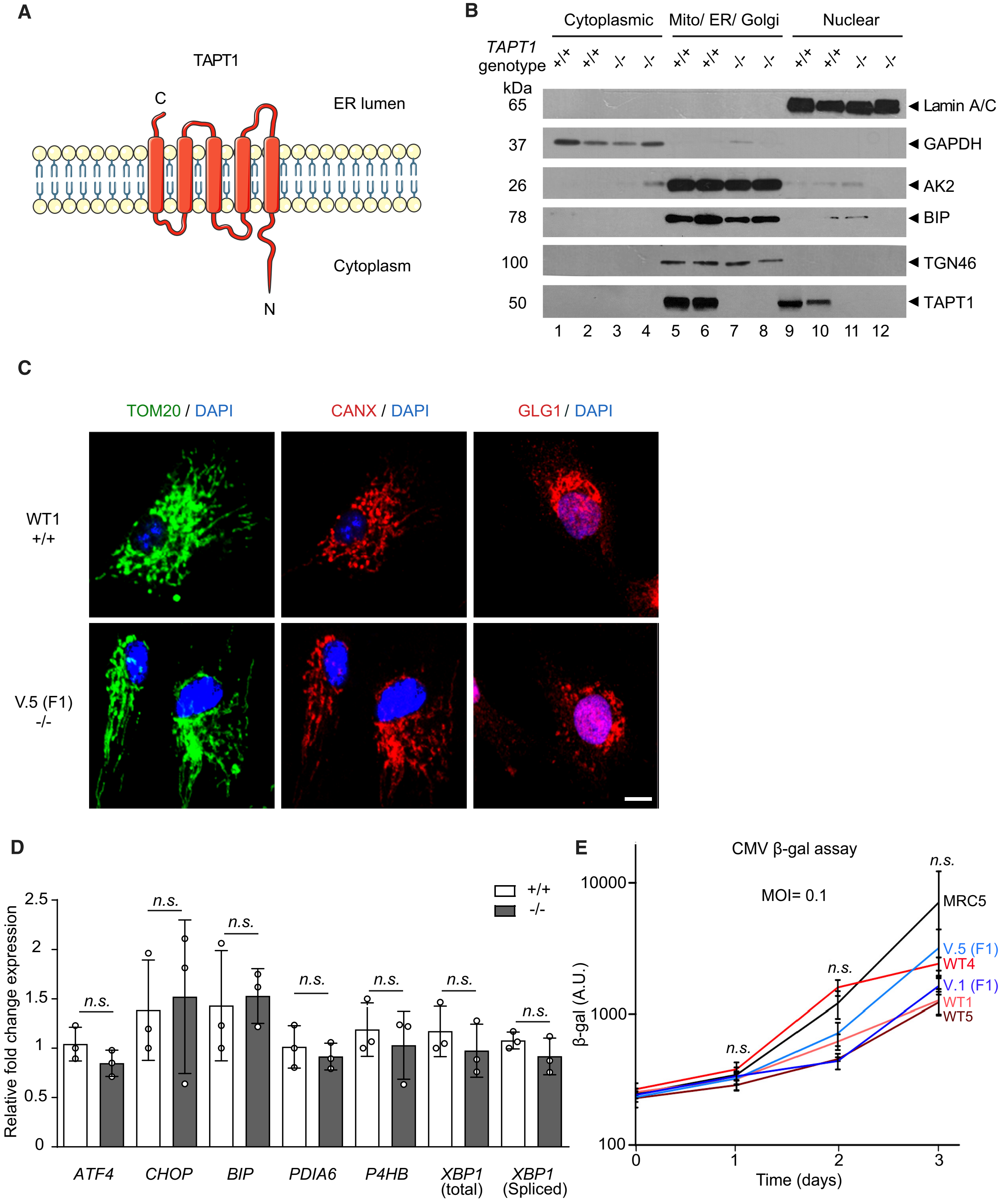
A
TAPT1
C
ER lumen
Cytoplasm
N
B
Cytoplasmic
Mito/ ER/ Golgi
Nuclear
TAPT1 genotype
kDa
65
37
26
78
100
50
Lamin A/C
GAPDH
AK2
BIP
TGN46
TAPT1
1 2 3 4 5 6 7 8 9 10 11 12
C
TOM20 / DAPI
CANX / DAPI
GLG1 / DAPI
WT1 +/+
V.5 (F1) -/-
D
Relative fold change expression
+/+
-/-
n.s.
ATF4
CHOP
BIP
PDIA6
P4HB
XBP1 (total)
XBP1 (Spliced)
E
CMV β-gal assay
MOI= 0.1
β-gal (A.U.)
MRC5
V.5 (F1)
WT4
V.1 (F1)
WT1
WT5
Time (days)

Figure 5.

◀ **Figure 5. TAPT1 cellular localization and functional data.**

A TAPT1 predicted topology: a membrane-spanning protein consisting of 5 transmembrane helices (Uniprot database).

B Western blot analyses for TAPT1 (~ 60 kDa) using cytosolic, Mito/ER/Golgi and nuclear protein extracts from primary dermal fibroblasts of two WTs (WT1 and WT2) and two patients (V.5 (F1) and IV.1 (F2)). TAPT1 protein is highly enriched in the Mito/ER/Golgi fraction, and to a lower extent in the nuclear fraction. GAPDH, TGN46 and BiP served as a cytosolic, Golgi network and ER markers, respectively. Adenylate Kinase (AK2) was used as a mitochondrial marker. Laminin A/C was used as a nuclear marker.

C Immunofluorescence staining of mitochondria using anti-TOM20 (green), ER using anti-CANX (red) and Golgi using anti-GLG1 (red) in primary dermal fibroblasts from WT1 and V.5 (F1). Similar staining patterns are observed with the three antibodies in both cell lines. Scale bar represents 10 μm.

D qPCR analysis of a panel of canonical ER stress markers shows no significant differences in 3 patients (−/−) (V.I (F1), V.5 (F1) and IV.1 (F2)) primary dermal fibroblasts compared with WTs (+/+) (WT1, WT2 and WT3) cells. Fold change relative to WT is plotted as mean ± SD. Statistical significance was tested by Student's *t*-test (n.s. *P*-value > 0.05).

E CMV cell infection assay on 2 patient (V.1 (F1) and V.5 (F1)) and 3 WT (WT1, WT4 and WT5) primary dermal fibroblast cell lines, using β-galactosidase activity as a readout. MRC5 cell line was used as a positive control. All of the cells were infected by the HCMV strain RC256 at a MOI = 0.1. Data are shown as mean ± SD of three technical replicates. Statistical significance was tested by Student's *t*-test (n.s. *P*-value > 0.05).

Source data are available online for this figure.

secretory pathway was supported by TAPT1 partner protein SUCO localization in various human cell lines (Hein *et al*, 2015).

We then carried out a series of functional tests by comparing WT and patient fibroblasts in order to gain a better understanding of TAPT1's cellular function. Several studies in yeast have reported that EMP65 is critically involved in the Unfolded Protein Response (UPR; Jonikas *et al*, 2009) and ER-Associated Degradation (ERAD) pathways (Zhang *et al*, 2017b). However, we could not document significant alterations in the expression levels of a panel of ER stress-associated markers in *TAPT1* knockout cells by qPCR (Fig 5D). IF staining did not reveal obvious defects in ER- (using anti-CANX antibody), GOLGI- (using anti-GLG1 antibody) or mitochondrial-morphology (using anti-TOM20 antibody) in patient *TAPT1* knockout cells compared with WT cells (Fig 5C).

Two early publications had reported that *TAPT1* encoded for a receptor of the human cytomegalovirus (HCMV) gH (Baldwin *et al*, 1996, 2000). We revisited this claim by testing whether human TAPT1-null patient cells were resistant to HCMV strain RC256 infection. The HCMV strain RC256 is a recombinant virus carrying the *Escherichia coli lacZ* gene as a marker under the control of the β-gal gene promoter (Spaete & Mocarski, 1987). Our β-gal reporter assay showed no discernable differences between WT and mutant patient cells (Fig 5E), hence indicating that TAPT1 is not essential for HCMV infection, further suggesting the likely presence of other cellular receptors which would permit HCMV cellular entry in the absence of TAPT1.

Extracellular matrix and collagen-related pathways are dysregulated in TAPT1-null cells

To gain insights into the cellular role of TAPT1 and the disease mechanism, we investigated which genes and pathways were altered in TAPT1-null patient cells. The combined analysis of RNA-seq and SI-NET-seq data was highly informative in this context. While RNA-seq quantifies the steady-state RNA levels, SI-NET-seq provides a quantitative measure of the RNA polymerase II (Pol II) occupancy with single-nucleotide precision genome-wide (Arnold *et al*, 2021). SI-NET-seq is an improved variant of the NET-seq approach (Mayer *et al*, 2015) that relies on spike-ins, thereby allowing quantitative comparisons between conditions (Arnold *et al*, 2021). While the RNA-seq data identified *TAPT1* as the only dysregulated gene on the candidate Chr.4 locus, it also provided an unbiased list of 172 significantly ($P < 0.05$) dysregulated genes in the patients' fibroblasts at the mRNA level (Fig 6A; Dataset EV1). Of these significantly altered genes, a similar fraction was up- and downregulated (Fig 6A; Dataset EV1). Beyond *TAPT1* which is the fifth most significantly downregulated gene in mutant cells, the dysregulation of several other target genes such as *RARRES2*, *ZIC1*, and *ZIC4* was validated by qPCR (Fig 6B). Moreover, SI-NET-seq results revealed a total of 317 genes with aberrant Pol II occupancy and dysregulated nascent RNAs in the patient cells (Figs 6C and EV5A and B; Dataset EV2). For the majority of these genes (70%), the density of transcriptionally engaged Pol II was significantly increased in patient cells (Fig 6C; Dataset EV2). The Pol II occupancy at *TAPT1* was not changed in patient cells, indicating that Pol II transcription of *TAPT1* was not impacted by the mutation (Fig 6C). Importantly, the integrated analysis of RNA-seq and SI-NET-seq data provided a comprehensive view on the molecular pathways that were affected by the *TAPT1* mutation. A subsequent pathway analysis of genes with either a significantly altered mRNA level or Pol II occupancy consistently revealed that extracellular matrix (ECM) organization and collagen-related pathways were highly enriched in our analysis (Fig 6D), further supporting a role for TAPT1 in the ECM and collagen dynamics.

Transcriptional compensation of ECM and collagen-related genes in parent cells

In heterozygous parent cells, the TAPT1 protein level was reduced but not completely abolished as in patient cells (Fig 4C). The remaining level of TAPT1 seemed to be sufficient for cell functions, thereby protecting the parents from a clinical manifestation. To gain insights into the underlying protection mechanism, we performed SI-NET-seq experiments on parent fibroblasts. SI-NET-seq revealed that nascent transcription was changed for fewer genes (170 genes) in parent cells (Fig EV5C; Dataset EV3) when compared with patient cells (317 genes). Surprisingly, an integrated Reactome pathway analysis of parent and patient fibroblasts uncovered a high overlap of affected pathways (Fig EV5D). However, from the 24 genes that were linked to the pathogenic pathways (Fig EV5E), only five genes (*COL3A1*, *COL5A2*, *COL8A1*, *COMP*, *ELN*) exhibited a significant change in nascent Pol II transcription in both parent and patient cells. The remaining deregulated genes in patient cells were not altered in parent cells

 © 2023 The Authors

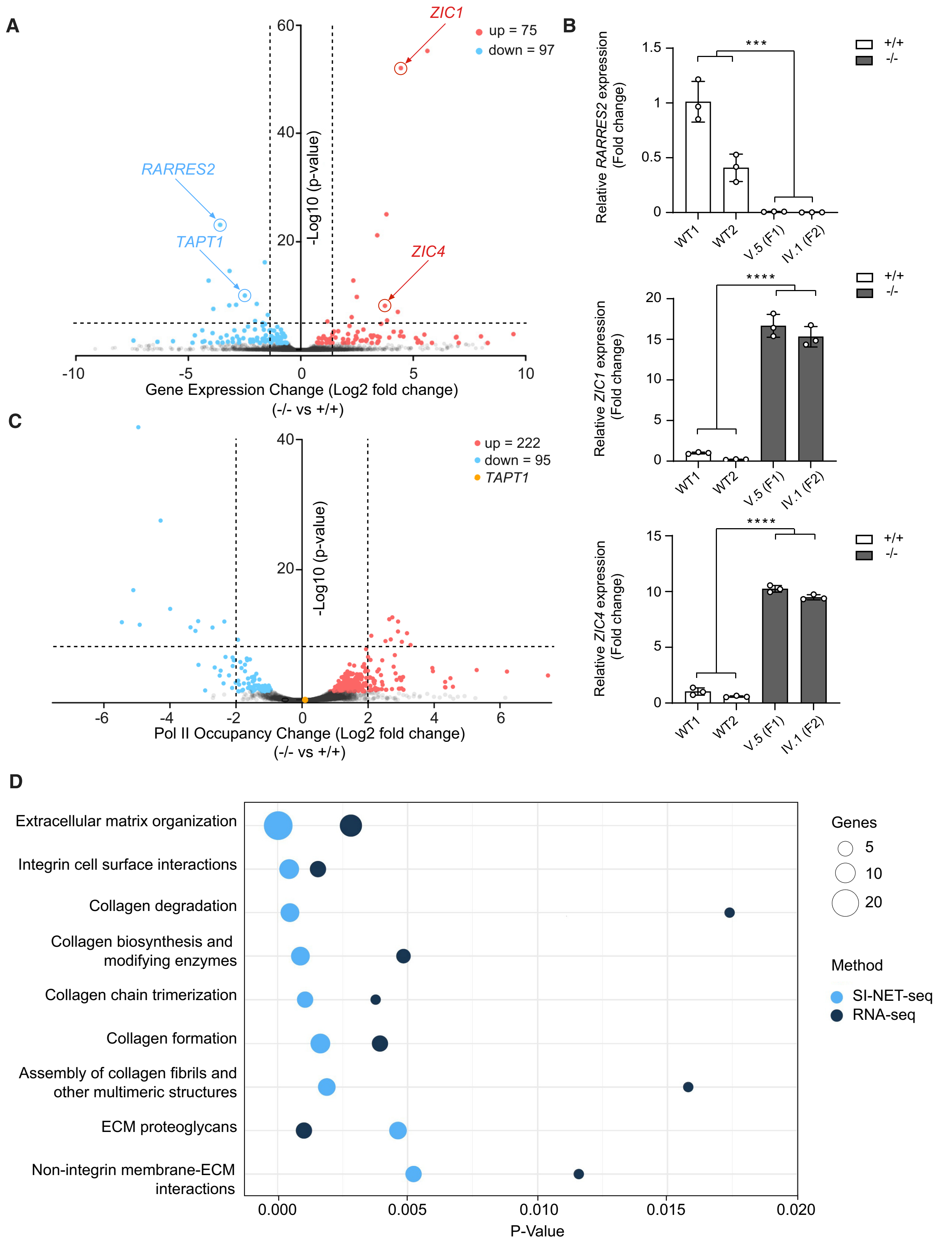
A
ZIC1
up = 75
down = 97
RARRES2
TAPT1
ZIC4
-Log10 (p-value)
Gene Expression Change (Log2 fold change)
(-/- vs +/+)
B
Relative RARRES2 expression
(Fold change)
+/+
-/-
WT1
WT2
V.5 (F1)
IV.1 (F2)
Relative ZIC1 expression
(Fold change)
Relative ZIC4 expression
(Fold change)
C
up = 222
down = 95
TAPT1
Pol II Occupancy Change (Log2 fold change)
(-/- vs +/+)
D
Extracellular matrix organization
Integrin cell surface interactions
Collagen degradation
Collagen biosynthesis and modifying enzymes
Collagen chain trimerization
Collagen formation
Assembly of collagen fibrils and other multimeric structures
ECM proteoglycans
Non-integrin membrane-ECM interactions
P-Value
Genes
Method
SI-NET-seq
RNA-seq

Figure 6.

Figure 6. Integrated analysis of SI-NET-seq and RNA-seq data revealed enrichment of collagen and ECM-related pathways in TAPT1-null cells.

A Volcano plot showing differentially expressed genes as determined by RNA-seq in patient primary dermal fibroblasts (V.1 (F1), V.5 (F1)) compared with WT (WT1 and WT2) cells. The y-axis shows the −log10 *P*-value, whereas the x-axis displays the log2 fold change value. The red dots represent 75 significantly upregulated genes, and the blue dots represent 97 significantly downregulated genes.

B qPCR validation test for 3 top dysregulated genes (*RARRES2*, *ZIC1*, and *ZIC4*) detected by RNA-seq. The analysis was performed on RNA samples independent from those sent for RNA-seq for 2 WTs (WT1 and WT2) and 2 patients (V.5 (F1) and IV.1 (F2)). Fold change relative to WT1 is plotted as mean ± SD of three technical replicates. Asterisks indicate statistical significance (Student's *t*-test; ****P*-value < 0.001, *****P*-value < 0.0001).

C Volcano plot showing genes with an altered occupancy of transcriptionally engaged Pol II in patients (V.1 (F1) and V.5 (F1)) compared with WT (WT1 and WT2) primary fibroblast cells using SI-NET-seq. The y-axis shows the −Log10 *P*-value, whereas the x-axis indicates the log2 fold change value for the Pol II occupancy. The Pol II density is increased in 222 genes (red dots), and decreased in 95 genes (blue dots). The yellow dot represents *TAPT1*.

D Bubble plot showing enrichment of collagen and extracellular matrix (ECM) pathways from the integrated reactome pathway analysis (Jassal *et al*, 2020) of the SI-NET-seq (light blue circles) and RNA-seq (dark blue circles) data. Enriched pathways are indicated on the y-axis, and the corresponding *P*-values are shown on the x-axis. The size of the circles represents the number of altered genes from each pathway.

(Fig EV5E). Instead, we detected the transcriptional activation of a set of genes (*CTSK*, *FN1*, *MME*, *MMP3*, *PPIB*) in parent cells associated with collagen- and ECM-related pathways. This significant transcriptional increase may partially compensate for the effects observed in patient cells. Together, these findings support the view that the deregulation of transcription in parent cells was below a potential syndrome-causing threshold.

Discussion

Phenotypic spectrum of TAPT1 insufficiency

Here, we report the successful identification of a genetic variant causing a recessive Mendelian syndrome in six affected children from 2 families presenting with severe bone defects, developmental delay and premature aging. As we did not detect any recessive mutations by WES, we followed an alternative analytical pipeline which involved homozygosity mapping, RNA-sequencing and targeted Sanger sequencing. We eventually identified a deep intronic mutation (c.1237-52 G>A) in the *TAPT1* gene that entirely segregated with the disease. It is known that pathogenic deep intronic mutations can induce splicing abnormalities, which in most cases lead to mRNA NMD due to the introduction of premature termination codons (PTCs) (Naruto *et al*, 2015; Fusco *et al*, 2019; Deng *et al*, 2020; Malekkou *et al*, 2020). Our prediction analysis suggested that the novel c.1237-52 G>A mutation is likely behaving as an alternative splicing branchpoint which triggers aberrant exon 12 skipping in the *TAPT1* pre-mRNA as confirmed by RT-PCR and the *in vitro* minigene splicing assay. This splicing aberration disrupts the reading frame and introduces a premature stop codon which targets mutant *TAPT1* transcripts for NMD in patient's fibroblasts. As expected and evidenced by western blotting data, the significant drop in *TAPT1* mRNA levels prevents the translation of a truncated protein. Moreover, SI-NET-seq showed that nascent transcription at the *TAPT1* gene was not affected by the mutation, confirming that the disease arises from a post-transcriptional dysregulation.

Genetic defects in *TAPT1* were firstly reported by Symoens *et al* (2015) in two consanguineous families with a complex and lethal osteochondrodysplasia syndrome (MIM616897) (Symoens *et al*, 2015). Furthermore, Patel *et al* (2017) reported a homozygous truncating mutation (c.846 + 2insT) in *TAPT1* segregating with pediatric cataract, although these patients did not show any evidence of skeletal defects (Patel *et al*, 2017). In addition to the shared clinical features with these previously reported TAPT1-deficient patients, including bone abnormalities and cataract, our affected children also suffered from neonatal progeria, characterized by wrinkled and thin skin, premature depigmentation and lipodystrophy. This vast phenotypic variation may be driven by the severity of the alleles identified. Our six new patients carry a complete loss-of-function mutation resulting in a protein-null allele, whereas both prior studies showed partial loss-of-function mutations including missense and in-frame exon 6 and exon 10 skipping (Symoens *et al*, 2015; Patel *et al*, 2017). Another possibility is that, as is the case for *LMNA* (Worman, 2012), a wide range of *TAPT1* diseases exist depending on which domain of the protein is mutated, thus accounting for the observed phenotypic heterogeneity.

TAPT1-deficiency resembles a collagenopathy

To date, RNA-seq stands out as the gold-standard technique to identify affected signaling pathways underlying a certain disease. To identify cellular processes that are affected upon *TAPT1* mutation, we performed an integrated pathway enrichment analysis combining RNA-seq and SI-NET-seq results. RNA-seq and SI-NET-seq uncovered genes with a significant change in transcript levels and nascent transcription in patient cells, respectively. Despite the different types of data, we observed a strong overlap in dysregulated pathways between both datasets. Collagen- and ECM-related pathways standout as the most significant hits from this combined analysis, indicating a dysregulation of these processes in patient cells. This interesting finding is consistent with our patients' phenotype, which manifests with severe bone defects and skin abnormalities. Collagens are the most abundant proteins made by the human body and serve to provide structural support, tensile strength while mediating cell adhesion, and migration (Frantz *et al*, 2010; Rozario & DeSimone, 2010). The bone tissue and the skin dermis account for 80% of the total collagen content of the body (Calleja-Agius *et al*, 2013). Importantly, the majority of genetic alterations causing bone defects affect collagen themselves e.g., COL1A1 (MIM114000, MIM619115, MIM130060, MIM166200, MIM166210, MIM259420, MIM166220, MIM166710), COL1A2 (MIM619120, MIM617821, MIM225320, MIM166210, MIM259420, MIM166220, MIM166710), or enzymes dedicated to their processing and secretion such as P3H1 (MIM610915), CRTAP (MIM610682) and TANGO1 (MIM619269) (Forlino & Marini, 2016; Lekszas *et al*, 2020; Guillemyn *et al*, 2021). Interestingly, SI-NET-seq in combination with pathway analysis for the heterozygous mother cells with reduced TAPT1 levels also showed a deregulation in collagen and ECM

 © 2023 The Authors

pathways. However, in parent cells less genes of the pathogenic pathways were altered and transcription of a set of genes that are associated with collagen- and ECM pathways were indeed activated, likely buffering the phenotypic consequences for the parent. The clinical manifestations and transcriptomics results shown here support the hypothesis that TAPT1-deficiency belongs to the heterogeneous group of collagenopathies.

Previous computational and experimental interactome analyses proposed that TAPT1 physically interacts with two additional ER-resident proteins: SUCO (SUN Domain Containing Ossification Factor; Hein *et al*, 2015; preprint: Parvez *et al*, 2020) and P4HB, also known as PDI1 (protein disulfide isomerase 1; preprint: Parvez *et al*, 2020). Notably, mutations in SUCO and P4HB have been linked to skeletal dysplasia (Rauch *et al*, 2015; Ouyang & Yang, 2017; Balasubramanian *et al*, 2018; Maddirevula *et al*, 2018; Porntaveetus *et al*, 2018; Li *et al*, 2019) in humans, which aligns with the TAPT1 loss-of-function clinical presentation. *Tapt1* and *Suco* mutant mice successfully phenocopy their corresponding human disease as they also present with severe skeletal defects (Howell *et al*, 2007; Sohaskey *et al*, 2010). These two proteins form a highly conserved complex which is present in all eukaryotic cells from yeast (Jonikas *et al*, 2009; Friederichs *et al*, 2012) to humans (Hein *et al*, 2015). EMP65 and SLP1, the yeast homologs for TAPT1 and SUCO respectively, have been shown to be involved in the ER quality-control machinery including UPR (Jonikas *et al*, 2009) and ERAD pathways (Zhang *et al*, 2017b). Surprisingly, our functional analyses did not reveal major abnormalities in the ER morphology and expression levels of ER stress markers in *TAPT1*-null cells. Previous research actually reported unaltered protein levels of ER chaperones, including BIP/GRP78, Calnexin, and GRP94, in *Suco*-null mouse osteoblasts (Sohaskey *et al*, 2010), which is consistent with our results assuming a common functional pathway for TAPT1 and SUCO. P4HB, the other proposed interacting partner for TAPT1, serves as a prototypic thiol isomerase that is involved in the hydroxylation of proline residues in collagen fibers (Annunen *et al*, 1997; Kukkola *et al*, 2003; Benham, 2012). Therefore, our data add to previous studies supporting the hypothesis of TAPT1, SUCO, and P4HB may form a functional complex residing in the ER/GOLGI and playing a key role in collagen post-translational processing with a particular relevance to skeletal development in vertebrate species. In accordance with this idea, delayed collagen folding and secretion was documented in *TAPT1* mutant fibroblasts (Symoens *et al*, 2015).

What could be TAPT1's universal function in eukaryotic cells?

Although loss-of-function mutations in *TAPT1*, *SUCO* and *P4HB* in humans all result in osteochondrodysplasia-like phenotypes, the homologs of these genes in lower organisms lead to unrelated phenotypes when absent. *POD1*, the TAPT1 homolog in *Arabidopsis*, was shown to be involved in pollen tube formation (Li *et al*, 2011). *F26F2.7*, the *Caenorhabditis elegans* homolog of *TAPT1* is a critical gene for embryonic viability with yet undetermined function (Maeda *et al*, 2001). In *Plasmodium falciparum*, the causative pathogen for malaria, mutations in *TAPT1's* homolog, *pfcarl*, confer resistance to various structurally unrelated antimalarial compounds which appear to target the ER/Golgi function of the parasite (Meister *et al*, 2011; Kuhen *et al*, 2014; LaMonte *et al*, 2016). In the unicellular fungus *Saccharomyces cerevisiae*, TAPT1 which is known as Emp65, is required for the stability of soluble proteins that are targeted to the secretory pathway (Zhang *et al*, 2017b). Notably, none of these species of plants, invertebrates or fungus possess genes coding for collagens, arguing that TAPT1's role in all eukaryotic cells must be unrelated to collagen biology *per se*, but instead fulfill a more essential cellular role that is yet to be deciphered.

Materials and Methods

Sample collection and clinical assessment

The affected children were firstly diagnosed at the National Center for Diabetes, Endocrinology and Genetics (NCDEG) (Amman, Jordan) with severe osteogenesis imperfecta. In total, 15 saliva samples were collected from members of the two families including parents, affected and unaffected siblings. Genomic DNA from saliva samples was isolated using the Origene DNA Collection Kit (OG-500, DNAGenotek). Skin biopsies from three affected (V.I (F1), V.5 (F1) and IV.1 (F2)) and one unaffected (IV.3 (F1)) family members were also collected. Informed consent was obtained from all individuals in accordance with local ethical review board requirements in Jordan and Singapore (A*STAR IRB reference code #2019-087, Singapore). Informed consent was also obtained from patients to publish their photos. All the experiments with human samples were in accordance with the principles set out in the WMA Declaration of Helsinki and the Department of Health and Human Services Belmont Report.

Genotyping and homozygosity mapping

SNP genotyping was performed on the genomic DNA from 15 affected and unaffected individuals from both families using Illumina HumanCoreExome-12v1 Bead-Chips. Identity-by-descent (IBD) mapping detected common homozygous regions in the 3 affected individuals using Wolfram Mathematica data-analysis software. IBD homozygous blocks were identified as regions > 2 cM. Candidate homozygous regions were refined by excluding the shared homozygous regions with unaffected individuals. Finally, a single identical and homozygous region was revealed on Chr. 4 (4p16.1–p15.31) (hg19).

Whole exome sequencing (WES)

The Ion TargetSeq™ Exome and Custom Enrichment Kit (Life Technologies) was used for exome capture from 1 μg of genomic DNA from individuals V.I (F1) and IV.1 (F2). The Ion OneTouch System (Life Technologies) was used for exome library preparation. Sequencing was performed using the Ion Proton Instrument (Life Technologies) with one Ion PI chip (Life Technologies). The variants were annotated with their associated gene and location. No candidate variant was found using various filtering parameters.

RNA-Sequencing

RNA from primary dermal fibroblasts from 2 patients (V.I (F1) and V.5 (F1)), and 2 unrelated wild types (WT1 and WT2) was extracted

© 2023 The Authors

using the RNeasy Mini Kit (Qiagen). After measuring RNA quantity and integrity using the Agilent Bioanalyzer 2100 (Agilent Technologies), libraries were sequenced on a Illumina HiSeq/Novaseq sequencer. Reads were aligned to the GRCh38.p12 human reference genome using STAR v2.5.3a (Dobin *et al*, 2013) with default parameters in paired-end mode.

For differential gene expression analysis, we quantified the transcript abundance of the annotated genes from GENCODE v28 (Frankish *et al*, 2019) using HTSeq v0.11.4 (Anders *et al*, 2015) in "union" mode. Significant changes between the conditions were tested using DESeq2 v1.25.4 (Love *et al*, 2014). We defined genes as significantly dysregulated when they had an FDR adjusted *P*-value of <0.05. For alternative splicing analysis, we focused on alternative exon inclusion and exclusion events between wild-type and patient samples. After read mapping, we identified all exons from GENCODE v28 annotation (Frankish *et al*, 2019) showing an "exon inclusion level" difference of at least 10% using rMATs v3.1.0 (Shen *et al*, 2014). The "exon inclusion level" of an exon describes the fraction of reads accounting for the inclusion of the exon. We defined alternative exon usage as an event between conditions with a significant (FDR < 0.05) difference in the "exon inclusion level". Splicing events that were supported by less than 5 reads were excluded.

SI-NET-sequencing

For spike-in NET-seq (SI-NET-seq), 15×10^6 primary dermal fibroblasts were mixed with 3×10^6 murine NIH 3T3 cells. Murine NIH 3T3 cells served as spike-in controls. The cells were tested negative for mycoplasma. All subsequent steps of the SI-NET-seq experiments were performed as recently described (Arnold *et al*, 2021) with the following modification. For reverse transcription of nascent RNAs, the SuperScript IV Reverse Transcriptase (ThermoFisher) was used.

Processing of SI-NET-seq data was performed as previously described (Arnold *et al*, 2021) with some modifications. Briefly, adaptor sequences and unique molecular identifiers (UMIs) were trimmed by cutadapt v2.4 (Martin, 2011) and a custom python script, which preserves information of UMI sequences for the corresponding reads. The obtained reads were aligned to a joined reference genome from human GRCh38.p12 and mouse GRCm38.p6 using the STAR v2.5.3a aligner (Dobin *et al*, 2013). For uniquely mapped reads, the position corresponding to the 3′-end of the nascent RNA fragment was recorded. We excluded reads that originated from reverse transcription mispriming and from PCR duplication using the UMI sequence information as described previously (Gajos *et al*, 2021). Additionally, sequenced splicing intermediates were excluded. We masked regions that were transcribed by Pol I and III, as well as loci of short chromatin-associated RNAs, which were extracted from annotations in GENCODE v28/v29 (Frankish *et al*, 2019; mouse: M18 and M22), RefSeq v109 (O'Leary *et al*, 2016), miRBase v22.1 (Kozomara *et al*, 2019) and the UCSC's RepeatMasker (Jurka *et al*, 2005). In the final step of data processing, we split the spiked-in mouse observations from sample observations.

We statistically tested the significance of changes in the Pol II occupancy. First, we quantified the Pol II occupancy at actively transcribed genes using SI-NET-seq data. Active genes had a calculated TPM value of at least one using RSEM v1.3.1 (Li & Dewey, 2011) quantifications from wild-type RNA-seq data. Second, we tested for significant changes in the Pol II occupancy using DEseq2 v1.25.4 (Love *et al*, 2014). For data normalization, we calculated the "Relative Log Expression" on Pol II occupancy measurements from spiked-in mouse cells. Quantification of Pol II occupancy in mouse was calculated as for sample observations. To define actively transcribed genes, we used RNA-seq data available for NIH3T3 mouse cells (ENCODE: ENCSR000CLW) (Davis *et al*, 2018). Changes in the Pol II occupancy at genes with an FDR adjusted *P*-value of 0.05 or smaller were considered significant.

Segregation analysis

The position coordinates and sequence of the candidate gene were obtained from the UCSC database. The region of the candidate mutation was amplified by PCR from genomic DNA from all 15 individuals using specific primers. Direct Sanger sequencing was performed on the PCR products using the BigDye Terminator Cycle Sequencing Kit (Applied Biosystems). Primer sequences are shown in Table EV1.

Cell culture and drug treatment

Primary dermal fibroblasts of affected and unaffected individuals were derived from skin biopsies following standard procedures (Vangipuram *et al*, 2013). All human cell lines were cultured at 5% CO_2 and 37°C in high glucose DMEM (HyClone) supplemented with 10% fetal bovine serum (FBS) (HyClone), 1X penicillin/streptomycin (Thermo Fisher Scientific) and 2 mM L-glutamine (Biological Industries), and tested negative for mycoplasma using the MycoAlert™ Mycoplasma Detection Kit (Lonza). Murine NIH 3 T3 cells (ATCC: CRL-1658) were grown in DMEM containing 10% FBS (Bovine Calf Serum, iron-fortified, Sigma) and 5% penicillin–streptomycin. To block NMD, the patient and WT cells were treated with 100 μg/ml of cycloheximide (CHX) for 4 and 8 h or with DMSO as a negative control (Rio Frio *et al*, 2008). Besides, RNA stability was checked by treating the cells with 5 μg/ml of actinomycin D (ActD) as a transcription inhibitor (Lai *et al*, 2019) for 0.5, 1 and 1.5 h.

Quantitative PCR

Total RNA was isolated from primary dermal fibroblasts using the RNeasy Mini Kit (Qiagen). RNA (1 μg) was reverse transcribed using the Iscript™ cDNA Synthesis Kit (Bio-Rad) according to the manufacturer's instructions. Transcript levels were assessed using the Power SYBR™ Green PCR Master Mix (Applied Biosystems) and specific primers (Table EV1) on the ABI Prism 7900HT Fast qPCR System (Applied Biosystems). qPCR assays involved three biological replicates per condition and three technical replicates per sample ($N = 3$, $n = 3$). *GAPDH* was used as the housekeeping gene to normalize gene expression.

Western blot

Total cellular protein extracts from primary dermal fibroblasts were obtained using RIPA buffer supplemented with 1X Protease

© 2023 The Authors

Inhibitor Cocktail (Roche). Nuclear, Mito/ER/Golgi, and Cytoplasmic fractions were prepared using the Cell Fractionation Kit Standard (Abcam, ab109719) following the manufacturer's instructions. Protein concentrations were measured using the Pierce™ BCA Protein Assay Kit (Thermo Fisher Scientific). Samples were reduced in Laemmli loading buffer containing dithiothreitol, and denatured at 95°C for 5 min. Equal amounts of protein were loaded on precast 10% Tris/Glycine/SDS polyacrylamide gradient gels (Bio-Rad), followed by transferring on PVDF membranes (Bio-Rad) using the Trans-Blot® Turbo™ Transfer System (Bio-Rad). Membranes were blocked in 5% milk in TBST for 1 h at room temperature, and subsequently probed with the following primary antibodies diluted in 5% milk in TBST overnight at 4°C: mouse anti-GAPDH (1:1,000; Santa-Cruz, sc-47724), rabbit anti-TAPT1 (1:1,000; Sigma, HPA042567, reacted with TAPT1 sequence covering exon 13–14), rabbit anti-TAPT1 (1:1,000; Sigma, HPA048658, reacted with TAPT1 sequence covering exon 6–8), mouse anti-BIP (1:1,000; BD Biosciences, 610978), rabbit anti-TGN-46 (1:1,000; Abcam, ab50595), rabbit anti-AK2 (1:1,000; Proteintech, 11014-1-AP) and mouse anti-Lamin A/C (1:1,000; EMD Millipore, MAB3211). After washes in TBST, secondary anti-mouse/HRP or anti-rabbit/HRP antibodies were used at 1:4,000 dilution in 5% milk in TBST for 1 h at room temperature. The signal was revealed with the SuperSignal™ West Chemiluminescent Substrate System (Thermo Fisher Scientific, #34080/34076/34096) and developed using CL-Xposure™ Films (Thermo Fisher Scientific) in a Carestream Kodak developer.

Immunofluorescence analysis

Primary dermal fibroblasts were cultured on 8-well glass chamber slides (Millicell EZ SLIDES) and fixed for 15 min in 4% paraformaldehyde in PBS at room temperature. The cells were permeabilized with 0.3% Triton-X100 in PBS for 15 min, and blocked in 1% BSA in PBS for 1 h at room temperature. Samples were then incubated with the following primary antibodies diluted in 1% BSA in PBS overnight at 4°C: rabbit anti-TAPT1 (1:1,000; Sigma, HPA042567), rabbit anti-TAPT1 (1:1,000; Sigma, HPA048658), rabbit anti-TOM20 (1:1,000; Proteintech, 11802-1-AP), rabbit anti-Calnexin (1:2,000; Abcam, ab22595) and mouse anti-GLG1 (1:500; Abcam, ab103439). For visualization, 1:500 secondary antibodies conjugated to Alexa Fluor 568 or Alexa Fluor 488 (Invitrogen, Molecular Probes) were incubated for 1 h at room temperature in the dark. 1 μg/ml DAPI (Life Technologies) was used for DNA staining, and cells were mounted using ProLong™ Diamond Antifade Mountant (Invitrogen). Images were captured using a FV1000 Olympus inverted confocal microscope equipped with a Leica camera.

Minigene splicing assay

To confirm the potential role of *TAPT1* deep intronic mutation (c.1237-52 G>A) in splicing defect, an *in vitro* minigene assay was done using pSPL3 exon trapping vector (Westin *et al*, 2021; Iturrate *et al*, 2022; Rodriguez-Muñoz *et al*, 2022). A genomic DNA fragment from patient cells (V.1(F1)) containing *TAPT1* exon 12 flanked by 200 bps upstream and 500 bps downstream intronic sequences were cloned into pSPL3 vector (Invitrogen) using *EcoR1/BamH1* restriction sites. Subsequently, the mutant construct was used as a template to generate a rescue construct by introducing c.1237-52 A>G change using QuickChange II XL kit (Agilent). Both mutant and rescue constructs were verified by direct Sanger sequencing. Then, HEK293T cells were transfected with 4 μg of DNA (pSPL3-c.1237-52 G>A, pSPL3-rescue or empty pSPL3 as a control) using Opti-MEM (Gibco) and Lipofectamine 3000 reagent (Invitrogen). Total RNA was extracted 24 h after transfection by NucleoSpin RNA kit and 3 μg of RNA was used for cDNA synthesis (Qiagen). To compare the splicing patterns of cells transfected with different constructs, RT–PCR was performed using vector-specific primers (SD6 and SA2). The PCR products were loaded on 2% agarose gel and purified after gel extraction. The transcripts were analyzed by direct Sanger sequencing. Primer sequences are shown in Table EV1.

The paper explained

Problems

A key step towards understanding Mendelian diseases is to identify the disease-causing mutation. A milestone in the identification of the relevant germline variations was the development of exome sequencing, the genome-wide determination of all exonic sequences encoding for proteins. However, 98% of the human genome is non-coding and escapes detection by exome sequencing including intronic regions. Since non-coding regions contain gene regulatory signals such as splicing regulatory sites, alterations in these genomic areas can have deleterious consequences for gene expression and cell function. Reliable strategies to uncover disease-causing variants in the non-coding portion of the genome and their functional consequences are still lacking.

Results

In this study, we used homozygosity mapping, RNA sequencing (RNA-seq) and targeted Sanger sequencing to identify a deep intronic mutation in the *TAPT1* gene (c.1237-52 G>A) in patient fibroblasts causing a recessive progeroid syndrome. An integrative analysis of spike-in controlled native elongating transcript sequencing (SI-NET-seq) and RNA-seq data provided insights into the molecular disease mechanisms caused by the intronic mutation. SI-NET-seq revealed that the intronic variant was inconsequential for nascent transcription at *TAPT1* pointing to a post-transcriptional defect. Further analyses showed enhanced skipping of *TAPT1* exon 12 *in vitro* and in patient cells which introduces a premature stop codon. The truncated RNA is rapidly degraded via the nonsense-mediated decay pathway resulting in a TAPT1-null allele. Finally, the transcriptomic data uncovered pathways involved in collagen and extracellular matrix biology as most significantly changed in patient cells. In parent cells, these pathways were less affected and buffered by a transcriptional compensation mechanism providing a plausible explanation for why parents had no clinical manifestation.

Impact

The integrative transcriptomic approach can now be applied to other diseases for which exome sequencing could not reveal the underlying cause. Our approach will help to identify new disease-causing mutations in the non-coding genome contributing to the ultimate goal of obtaining a systematic map for non-coding pathogenic variants of the human genome. At the same time, RNA-/SI-NET-seq data can illuminate the molecular disease mechanisms and pathways that are significantly impacted. A more complete understanding of the causes and the pathogenic mechanisms of these diseases will improve diagnosis and holds the promise to open new strategies for therapeutic interventions.

© 2023 The Authors

GapmeR transfection

Primary dermal fibroblasts were seeded on 6-well plates at a density of 100,000 cells per well. The following day, cells were transfected using the Lipofectamine RNAiMAX Reagent (Invitrogen) with two GapmeRs specific for *TAPT1-AS1* and a non-targeted GapmeR as a negative control at a 40 nM concentration. The GapmeRs were purchased from Qiagen (Germany), and their sequences are given in Table EV1. A 72 h post-transfection, RNA and protein were harvested for downstream experiments.

CMV β-Gal assay

20,000–40,000 cells were plated per well in 96-well plates. The MRC5 human lung fibroblast cell line was used as a positive control. The next day after seeding, the virus (CMV strain RC256 ATCC VR-2356) was added in DMEM supplemented with 10% FBS at MOI = 0.1 and absorbed for 1 h at room temperature. Then the virus was aspirated off and the plates were carefully washed twice with PBS. The 80 µl of DMEM supplemented with 10% FBS were added back to each well and the plate was returned to the incubator. β-gal activity was read at different time points (Days 0, 1, 2 and 3), involving three replicates per time point and per cell line. For that purpose, 20 µl of 5X lysis buffer (500 mM K-phos pH 7.8, 1% Triton X-100) were first added to each well. After pipetting up and down, samples were incubated for 15 min at 37°C. Then, 10 µl of the lysates were transferred to a plate with Galacto-Star, which was subsequently covered with foil and incubated at room temperature for 20 min. The signal was finally measured with a luminescence plate reader.

Statistical analyses

The statistical comparison of two groups were made by two-tailed Student's *t*-test. The values are presented as mean ± SD. *P*-value < 0.05 was considered statistically significant. All of the experiments were done in at least three technical replicates.

Data availability

The RNA-seq and SI-NET-seq datasets produced in this study are available in the Gene Expression Omnibus (GSE197120; https://www.ncbi.nlm.nih.gov/geo/query/acc.cgi?acc=GSE197120) database.

Expanded View for this article is available online.

Acknowledgements

B.R. is a fellow of the Branco Weiss Foundation and EMBO Young Investigator. This work was also supported by a Strategic Positioning Fund on Genetic Orphan Diseases (GODAFIT) and an Use-Inspired Basic Research (UIBR) grant from Agency for Science, Technology and Research (A*STAR) in Singapore to B.R. This work was also funded by the Max Planck Society (to A.M.) and the Deutsche Forschungsgemeinschaft (DFG, grant 418415292 to A. M.). This work was also supported by a 2232 International Fellowship for Outstanding Researchers Program of TÜBİTAK (Project No: 118C318 to N.E.B). Funding for the open access charge was provided by the Max Planck Society. We are profoundly grateful to all patient family members for their participation in this study. We would like to thank all members of Reversade laboratory for their kind help and support. We would like to thank Dr. Shokouh Karimi for her help in careful clinical evaluation. We would also acknowledge Dr. Victor L. Ruiz-Perez for his generosity in providing the pSPL3 trapping vector for this study. We thank the Sequencing facility of the MPI for Molecular Genetics for sequencing.

Author contributions

Nasrinsadat Nabavizadeh: Conceptualization; data curation; software; formal analysis; validation; investigation; visualization; methodology; writing—original draft; project administration; writing—review and editing. **Annkatrin Bressin:** Data curation; software; formal analysis; methodology; writing—review and editing. **Mohammad Shboul:** Methodology; writing—review and editing. **Ricardo Moreno Traspas:** Validation; methodology; writing—review and editing. **Poh Hui Chia:** Supervision; methodology; writing—original draft. **Carine Bonnard:** Formal analysis; methodology; project administration; writing—review and editing. **Emmanuelle Szenker-Ravi:** Validation; methodology; writing—review and editing. **Burak Sarıbaş:** Validation; methodology. **Emmanuel Beillard:** Methodology; writing—review and editing. **Umut Altunoglu:** Methodology. **Zohreh Hojati:** Methodology. **Scott Drutman:** Validation; methodology. **Susanne Freier:** Methodology. **Mohammad El-Khateeb:** Methodology. **Rajaa Fathallah:** Methodology. **Jean-Laurent Casanova:** Methodology. **Wesam Soror:** Methodology. **Alaa Arafat:** Methodology. **Nathalie Escande-Beillard:** Resources; validation; methodology; writing—review and editing. **Andreas Mayer:** Conceptualization; resources; data curation; software; formal analysis; supervision; funding acquisition; methodology; writing—review and editing. **Bruno Reversade:** Conceptualization; resources; data curation; software; formal analysis; supervision; funding acquisition; validation; investigation; visualization; methodology; writing—original draft; project administration; writing—review and editing.

Disclosure and competing interest statement

None of the authors have any financial interest related to this work and therefore declare no conflict of interest.

For more information

Online Mendelian Inheritance in Man (OMIM): https://www.omim.org/
PubMed: https://pubmed.ncbi.nlm.nih.gov/
UCSC Genome Browser: https://genome.ucsc.edu/
Ensembl genome browser: https://www.ensembl.org/index.html
UniProt: www.uniprot.org
Reactome: https://reactome.org/
Reversade Lab: www.reversade.com
Mayer Lab: www.molgen.mpg.de/mayer-lab

References

Anders S, Pyl PT, Huber W (2015) HTSeq--a python framework to work with high-throughput sequencing data. *Bioinformatics* 31: 166–169

Annunen P, Helaakoski T, Myllyharju J, Veijola J, Pihlajaniemi T, Kivirikko KI (1997) Cloning of the human prolyl 4-hydroxylase alpha subunit isoform alpha(II) and characterization of the type II enzyme tetramer. The alpha(I) and alpha(II) subunits do not form a mixed alpha(I)alpha(II)beta2 tetramer. *J Biol Chem* 272: 17342–17348

Arnold M, Bressin A, Jasnovidova O, Meierhofer D, Mayer A (2021) A BRD4-mediated elongation control point primes transcribing RNA polymerase II for 3′-processing and termination. *Mol Cell* 81: 3589–3603.e13

 © 2023 The Authors

Balasubramanian M, Padidela R, Pollitt RC, Bishop NJ, Mughal MZ, Offiah AC, Wagner BE, McCaughey J, Stephens DJ (2018) P4HB recurrent missense mutation causing Cole-carpenter syndrome. *J Med Genet* 55: 158 – 165

Baldwin BR, Kleinberg M, Keay S (1996) Molecular cloning and expression of receptor peptides that block human cytomegalovirus/cell fusion. *Biochem Biophys Res Commun* 219: 668 – 673

Baldwin BR, Zhang CO, Keay S (2000) Cloning and epitope mapping of a functional partial fusion receptor for human cytomegalovirus gH. *J Gen Virol* 81: 27 – 35

Benham AM (2012) The protein disulfide isomerase family: key players in health and disease. *Antioxid Redox Signal* 16: 781 – 789

Calleja-Agius J, Brincat M, Borg M (2013) Skin connective tissue and ageing. *Best Pract Res Clin Obstet Gynaecol* 27: 727 – 740

Chen J, Ma N, Zhao X, Li W, Zhang Q, Yuan S, Tan YQ, Lu G, Lin G, Du J (2019) A rare deep intronic mutation of PKHD1 gene, c.8798-459C>a, causes autosomal recessive polycystic kidney disease by pseudoexon activation. *J Hum Genet* 64: 207 – 214

Chong JX, Buckingham KJ, Jhangiani SN, Boehm C, Sobreira N, Smith JD, Harrell TM, McMillin MJ, Wiszniewski W, Gambin T *et al* (2015) The genetic basis of mendelian phenotypes: discoveries, challenges, and opportunities. *Am J Hum Genet* 6;97(2):199-215 97: 199 – 215

Cummings BB, Marshall JL, Tukiainen T, Lek M, Donkervoort S, Foley AR, Bolduc V, Waddell LB, Sandaradura SA, O'Grady GL *et al* (2017) Improving genetic diagnosis in mendelian disease with transcriptome sequencing. *Sci Transl Med* 9: eaal5209

Davis CA, Hitz BC, Sloan CA, Chan ET, Davidson JM, Gabdank I, Hilton JA, Jain K, Baymuradov UK, Narayanan AK *et al* (2018) The encyclopedia of DNA elements (ENCODE): data portal update. *Nucleic Acids Res* 46: D794 – D801

Deng J, Li D, Mei H, Tang L, Wang HF, Hu Y (2020) Novel deep intronic mutation in the coagulation factor XIII a chain gene leading to unexpected RNA splicing in a patient with factor XIII deficiency. *BMC Med Genet* 21: 9

Djebali S, Davis CA, Merkel A, Dobin A, Lassmann T, Mortazavi A, Tanzer A, Lagarde J, Lin W, Schlesinger F *et al* (2012) Landscape of transcription in human cells. *Nature* 489: 101 – 108

Dobin A, Davis CA, Schlesinger F, Drenkow J, Zaleski C, Jha S, Batut P, Chaisson M, Gingeras TR (2013) STAR: ultrafast universal RNA-seq aligner. *Bioinformatics* 29: 15 – 21

Evrony GD, Cordero DR, Shen J, Partlow JN, Yu TW, Rodin RE, Hill RS, Coulter ME, Lam AN, Jayaraman D *et al* (2017) Integrated genome and transcriptome sequencing identifies a noncoding mutation in the genome replication factor DONSON as the cause of microcephaly-micromelia syndrome. *Genome Res* 27: 1323 – 1335

Faghihi MA, Wahlestedt C (2009) Regulatory roles of natural antisense transcripts. *Nat Rev Mol Cell Biol* 10: 637 – 643

Faghihi MA, Modarresi F, Khalil AM, Wood DE, Sahagan BG, Morgan TE, Finch CE, St Laurent G 3rd, Kenny PJ, Wahlestedt C (2008) Expression of a noncoding RNA is elevated in Alzheimer's disease and drives rapid feed-forward regulation of beta-secretase. *Nat Med* 14: 723 – 730

Forlino A, Marini JC (2016) Osteogenesis imperfecta. *Lancet* 387: 1657 – 1671

Frankish A, Diekhans M, Ferreira AM, Johnson R, Jungreis I, Loveland J, Mudge JM, Sisu C, Wright J, Armstrong J *et al* (2019) GENCODE reference annotation for the human and mouse genomes. *Nucleic Acids Res* 47: D766 – D773

Frantz C, Stewart KM, Weaver VM (2010) The extracellular matrix at a glance. *J Cell Sci* 15: 4195 – 4200

Friederichs JM, Gardner JM, Smoyer CJ, Whetstine CR, Gogol M, Slaughter BD, Jaspersen SL (2012) Genetic analysis of Mps3 SUN domain mutants in Saccharomyces cerevisiae reveals an interaction with the SUN-like protein Slp1. *G3* 2: 1703 – 1718

Fusco C, Morlino S, Micale L, Ferraris A, Grammatico P, Castori M (2019) Characterization of two novel intronic variants affecting splicing in FBN1-related disorders. *Genes* 10: 442

Gajos M, Jasnovidova O, van Bömmel A, Freier S, Vingron M, Mayer A (2021) Conserved DNA sequence features underlie pervasive RNA polymerase pausing. *Nucleic Acids Res* 49: 4402 – 4420

Guillemyn B, Nampoothiri S, Syx D, Malfait F, Symoens S (2021) Loss of TANGO1 leads to absence of bone mineralization. *JBMR Plus* 5: e10451

Hein MY, Hubner NC, Poser I, Cox J, Nagaraj N, Toyoda Y, Gak IA, Weisswange I, Mansfeld J, Buchholz F *et al* (2015) A human interactome in three quantitative dimensions organized by stoichiometries and abundances. *Cell* 163: 712 – 723

Howell GR, Shindo M, Murray S, Gridley T, Wilson LA, Schimenti JC (2007) Mutation of a ubiquitously expressed mouse transmembrane protein (Tapt1) causes specific skeletal homeotic transformations. *Genetics* 175: 699 – 707

Iturrate A, Rivera-Barahona A, Flores CL, Otaify GA, Elhossini R, Perez-Sanz ML, Nevado J, Tenorio-Castano J, Triviño JC, Garcia-Gonzalo FR *et al* (2022) Mutations in SCNM1 cause orofaciodigital syndrome due to minor intron splicing defects affecting primary cilia. *Am J Hum Genet* 109: 1828 – 1849

Jamshidi F, Place EM, Mehrotra S, Navarro-Gomez D, Maher M, Branham KE, Valkanas E, Cherry TJ, Lek M, MacArthur D *et al* (2019) Contribution of noncoding pathogenic variants to RPGRIP1-mediated inherited retinal degeneration. *Genet Med* 21: 694 – 704

Jassal B, Matthews L, Viteri G, Gong C, Lorente P, Fabregat A, Sidiropoulos K, Cook J, Gillespie M, Haw R *et al* (2020) The reactome pathway knowledgebase. *Nucleic Acids Res* 48: D498 – D503

Jonikas MC, Collins SR, Denic V, Oh E, Quan EM, Schmid V, Weibezahn J, Schwappach B, Walter P, Weissman JS *et al* (2009) Comprehensive characterization of genes required for protein folding in the endoplasmic reticulum. *Science* 323: 1693 – 1697

Jurka J, Kapitonov VV, Pavlicek A, Klonowski P, Kohany O, Walichiewicz J (2005) Repbase update, a database of eukaryotic repetitive elements. *Cytogenet Genome Res* 110: 462 – 467

Khan AO, Becirovic E, Betz C, Neuhaus C, Altmüller J, Maria Riedmayr L, Motameny S, Nürnberg G, Nürnberg P, Bolz HJ (2017) A deep intronic CLRN1 (USH3A) founder mutation generates an aberrant exon and underlies severe usher syndrome on the Arabian peninsula. *Sci Rep* 7: 1411

Kozomara A, Birgaoanu M, Griffiths-Jones S (2019) miRBase: from microRNA sequences to function. *Nucleic Acids Res* 47: D155 – D162

Kremer LS, Bader DM, Mertes C, Kopajtich R, Pichler G, Iuso A, Haack TB, Graf E, Schwarzmayr T, Terrile C *et al* (2017) Genetic diagnosis of mendelian disorders via RNA sequencing. *Nat Commun* 12: 15824

Kuhen KL, Chatterjee AK, Rottmann M, Gagaring K, Borboa R, Buenviaje J, Chen Z, Francek C, Wu T, Nagle A *et al* (2014) KAF156 is an antimalarial clinical candidate with potential for use in prophylaxis, treatment, and prevention of disease transmission. *Antimicrob Agents Chemother* 58: 5060 – 5067

Kukkola L, Hieta R, Kivirikko KI, Myllyharju J (2003) Identification and characterization of a third human, rat, and mouse collagen prolyl 4-hydroxylase isoenzyme. *J Biol Chem* 278: 47685 – 47693

Lai WS, Arvola RM, Goldstrohm AC, Blackshear PJ (2019) Inhibiting transcription in cultured metazoan cells with actinomycin D to monitor mRNA turnover. *Methods* 15: 77–87

LaMonte G, Lim MY, Wree M, Reimer C, Nachon M, Corey V, Gedeck P, Plouffe D, Du A, Figueroa N *et al* (2016) Mutations in the plasmodium falciparum cyclic amine resistance locus (PfCARL) confer multidrug resistance. *mBio* 7: e00696-16

LaMonte GM, Rocamora F, Marapana DS, Gnädig NF, Ottilie S, Luth MR, Worgall TS, Goldgof GM, Mohunlal R, Santha Kumar TR *et al* (2020) Pan-active imidazolopiperazine antimalarials target the plasmodium falciparum intracellular secretory pathway. *Nat Commun* 11: 1780–1711

Lekszas C, Foresti O, Raote I, Liedtke D, König EM, Nanda I, Vona B, De Coster P, Cauwels R, Malhotra V *et al* (2020) Biallelic TANGO1 mutations cause a novel syndromal disease due to hampered cellular collagen secretion. *eLife* 26: e51319

Li B, Dewey CN (2011) RSEM: accurate transcript quantification from RNA-seq data with or without a reference genome. *BMC Bioinformatics* 4: 323

Li HJ, Xue Y, Jia DJ, Wang T, Hi DQ, Liu J, Cui F, Xie Q, Ye D, Yang WC (2011) POD1 regulates pollen tube guidance in response to micropylar female signaling and acts in early embryo patterning in *Arabidopsis*. *Plant Cell* 23: 3288–3302

Li L, Zhao D, Zheng W, Wang O, Jiang Y, Xia W, Xing X, Li M (2019) A novel missense mutation in P4HB causes mild osteogenesis imperfecta. *Biosci Rep* 39: BSR20182118

Lloret-Llinares M, Mapendano CK, Martlev LH, Lykke-Andersen S, Jensen TH (2016) Relationships between PROMPT and gene expression. *RNA Biol* 13: 6–14

Love MI, Huber W, Anders S (2014) Moderated estimation of fold change and dispersion for RNA-seq data with DESeq2. *Genome Biol* 15: 550

Maddirevula S, Alsahli S, Alhabeeb L, Patel N, Alzahrani F, Shamseldin HE, Anazi S, Ewida N, Alsaif HS, Mohamed JY *et al* (2018) Expanding the phenome and variome of skeletal dysplasia. *Genet Med* 20: 1609–1616

Maeda I, Kohara Y, Yamamoto M, Sugimoto A (2001) Large-scale analysis of gene function in *Caenorhabditis elegans* by high-throughput RNAi. *Curr Biol* 11: 171–176

Malekkou A, Sevastou I, Mavrikiou G, Georgiou T, Vilageliu L, Moraitou M, Michelakakis H, Prokopiou C, Drousiotou A (2020) A novel mutation deep within intron 7 of the GBA gene causes Gaucher disease. *Mol Genet Genomic Med* 8: e1090

Martin M (2011) Cutadapt removes adapter sequences from high-throughput sequencing reads. *EMBnetjournal* 17: 10–12

Mayer A, di Iulio J, Maleri S, Eser U, Vierstra J, Reynolds A, Sandstrom R, Stamatoyannopoulos JA, Churchman LS (2015) Native elongating transcript sequencing reveals human transcriptional activity at nucleotide resolution. *Cell* 161: 541–554

Meister S, Plouffe DM, Kuhen KL, Bonamy GM, Wu T, Barnes SW, Bopp SE, Borboa R, Bright AT, Che J *et al* (2011) Imaging of plasmodium liver stages to drive next-generation antimalarial drug discovery. *Science* 334: 1372–1377

Naruto T, Okamoto N, Masuda K, Endo T, Hatsukawa Y, Kohmoto T, Imoto I (2015) Deep intronic GPR143 mutation in a Japanese family with ocular albinism. *Sci Rep* 10: 11334

Nazari I, Tayara H, Chong KT (2018) Branch point selection in RNA splicing using deep learning. *IEEE Access* 13: 1800–1807

O'Leary NA, Wright MW, Brister JR, Ciufo S, Haddad D, McVeigh R, Rajput B, Robbertse B, Smith-White B, Ako-Adjei D *et al* (2016) Reference sequence (RefSeq) database at NCBI: current status, taxonomic expansion, and functional annotation. *Nucleic Acids Res* 44: D733–D745

Ouyang L, Yang F (2017) Cole-carpenter syndrome-1 with a de novo heterozygous deletion in the P4HB gene in a Chinese girl: a case report. *Medicine* 96: e9504

Paggi JM, Bejerano G (2018) A sequence-based, deep learning model accurately predicts RNA splicing branchpoints. *RNA* 24: 1647–1658

Parvez MSA, Rahman MM, Morshed MN, Rahman D, Anwar S, Coucke P, Hosen M (2020) Novel insights into the structure and transport mechanisms of TAPT1. *bioRxiv* https://doi.org/10.1101/2020.05.18.099887 [PREPRINT]

Patel N, Anand D, Monies D, Maddirevula S, Khan AO, Algoufi T, Alowain M, Faqeih E, Alshammari M, Qudair A *et al* (2017) Novel phenotypes and loci identified through clinical genomics approaches to pediatric cataract. *Hum Genet* 136: 205–225

Pelechano V, Steinmetz LM (2013) Gene regulation by antisense transcription. *Nat Rev Genet* 14: 880–893

Porntaveetus T, Theerapanon T, Srichomthong C, Shotelersuk V (2018) Cole-carpenter syndrome in a patient from Thailand. *Am J Med Genet A* 176: 1706–1710

Rauch F, Fahiminiya S, Majewski J, Carrot-Zhang J, Boudko S, Glorieux F, Mort JS, Bächinger HP, Moffatt P (2015) Cole-carpenter syndrome is caused by a heterozygous missense mutation in P4HB. *Am J Hum Genet* 96: 425–431

Rio Frio T, Wade NM, Ransijn A, Berson EL, Beckmann JS, Rivolta C (2008) Premature termination codons in PRPF31 cause retinitis pigmentosa via haploinsufficiency due to nonsense-mediated mRNA decay. *J Clin Invest* 118: 1519–1531

Rodriguez-Muñoz A, Liquori A, García-Bohorquez B, Jaijo T, Aller E, Millán JM, García-García G (2022) Functional assays of non-canonical splice-site variants in inherited retinal dystrophies genes. *Sci Rep* 12: 68

Rozario T, DeSimone DW (2010) The extracellular matrix in development and morphogenesis: a dynamic view. *Dev Biol* 341: 126–140

Seila AC, Core LJ, Lis JT, Sharp PA (2009) Divergent transcription: a new feature of active promoters. *Cell Cycle* 8: 2557–2564

Shen S, Park JW, Lu ZX, Lin L, Henry MD, Wu YN, Zhou Q, Xing Y (2014) rMATS: robust and flexible detection of differential alternative splicing from replicate RNA-seq data. *Proc Natl Acad Sci USA* 111: E5593–E5601

Sigova AA, Mullen AC, Molinie B, Gupta S, Orlando DA, Guenther MG, Almada AE, Lin C, Sharp PA, Giallourakis CC *et al* (2013) Divergent transcription of long noncoding RNA/mRNA gene pairs in embryonic stem cells. *Proc Natl Acad Sci USA* 110: 2876–2881

Sohaskey ML, Jiang Y, Zhao JJ, Mohr A, Roemer F, Harland RM (2010) Osteopotentia regulates osteoblast maturation, bone formation, and skeletal integrity in mice. *J Cell Biol* 189: 511–525

Spaete RR, Mocarski ES (1987) Insertion and deletion mutagenesis of the human cytomegalovirus genome. *Proc Natl Acad Sci USA* 84: 7213–7217

Symoens S, Barnes AM, Gistelinck C, Malfait F, Guillemyn B, Steyaert W, Syx D, D'hondt S, Biervliet M, De Backer J *et al* (2015) Genetic defects in TAPT1 disrupt Ciliogenesis and cause a complex lethal Osteochondrodysplasia. *Am J Hum Genet* 97: 521–534

Vangipuram M, Ting D, Kim S, Diaz R, Schüle B (2013) Skin punch biopsy explant culture for derivation of primary human fibroblasts. *J Vis Exp* 7: e3779

Westin IM, Jonsson F, Österman L, Holmberg M, Burstedt M, Golovleva I (2021) EYS mutations and implementation of minigene assay for variant

 © 2023 The Authors

classification in EYS-associated retinitis pigmentosa in northern Sweden. *Sci Rep* 11: 7696

Worman HJ (2012) Nuclear lamins and laminopathies. *J Pathol* 226: 316 – 325

Yu W, Gius D, Onyango P, Muldoon-Jacobs K, Karp J, Feinberg AP, Cui H (2008) Epigenetic silencing of tumour suppressor gene p15 by its antisense RNA. *Nature* 451: 202 – 206

Zhang Q, Fan X, Wang Y, Sun MA, Shao J, Guo D (2017a) BPP: a sequence-based algorithm for branch point prediction. *Bioinformatics* 33: 3166 – 3172

Zhang S, Xu C, Larrimore KE, Ng DTW (2017b) Slp1-Emp65: a Guardian factor that protects folding polypeptides from promiscuous degradation. *Cell* 171: 346 – 357.e12

License: This is an open access article under the terms of the Creative Commons Attribution License, which permits use, distribution and reproduction in any medium, provided the original work is properly cited.

Expanded View Figures

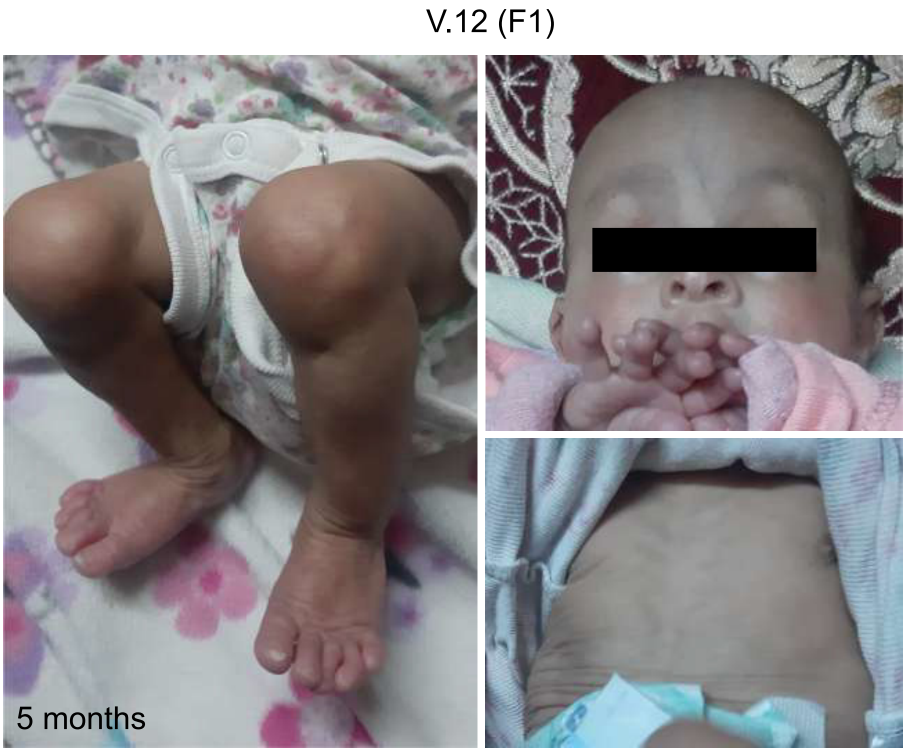

Figure EV1. Clinical pictures of the affected V.12 (F1) individual.

The patient presented with multiple abnormalities including bone and joint deformities, pectus excavatum, plagiocephaly microphthalmia and bilateral hypotropia. Moreover, she had apparent dysmorphic facial features such as a depressed nasal bridge and low set of ears.

Figure EV2. Overlap analysis from homozygosity mapping and RNA-seq data revealed TAPT1 as the only candidate gene.

A List of the 39 candidate genes located in the mapped Chr. 4 IBD locus.

B Lists of the top 10 significantly downregulated (left, blue) and upregulated (right, red) genes obtained from our RNA-seq differential expression analysis.

C Expression changes (x-axis, log2FC) for genes with at least one alternative splicing event (skipped exon (SE), retained exon (RE), mutually exclusive exon (MXE), alternative 3′ or 5′ splice site (A3SS and A5SS) and retained intron (RI)).

D (Top) Venn diagram displaying overlapping genes between the Chr. 4 IBD candidate locus, and the top 10 upregulated and downregulated genes from our RNA-seq data analysis. (Bottom) Venn diagram showing the overlapping genes between the differentially expressed set and the alternative spliced set from our RNA-seq data analysis. *TAPT1* appears as the only overlapping gene in both diagrams.

© 2023 The Authors

A

List of 39 genes in mapped Chr. 4 IBD candidate locus			
MIR572	*MIR5091*	*FAM200B*	*SNORA75B*
HS3ST1	*LINC01182*	*BST1*	*QDPR*
LINC02360	*LINC01085*	*CD38*	*CLRN2*
MIR12113	*LINC00504*	*FGFBP1*	*LAP3*
LINC02270	*CPEB2-DT*	*FGFBP2*	*DCAF16*
RAB28	*CPEB2*	*PROM1*	*NCAPG*
LINC01097	*C1QTNF7-AS1*	*TAPT1-AS1*	*MED28*
NKX3-2	*C1QTNF7*	*TAPT1*	*FAM184B*
LINC01096	*CC2D2A*	*LDB2*	*LCORL*
BOD1L1	*FBXL5*	*LINC02493*	

B

List of top significant downregulated genes			List of top significant upregulated genes		
**Gene name**	**-log10 (padj)**	**Log2 fold change**	**Gene name**	**-log10 (padj)**	**Log2 fold change**
RARRES2	23.2	-3.57	*CTC-575D19.1*	55.31	5.65
DHRS3	16.25	-1.59	*ZIC1*	52.13	4.47
SULF1	14.65	-3.15	*DES*	25.15	3.83
HLA-H	12.86	-4.09	*RP11-565J7.3*	21.25	3.42
TAPT1	10.1	-2.47	*NTNG1*	12.88	2.36
IKZF2	8.54	-1.95	*POSTN*	9.84	2.51
BEX1	8.39	-2.86	*ZIC4*	8.2	3.77
BAMBI	8.28	-3.17	*BCHE*	7.07	4.34
SRGN	7.62	-3.88	*DNM1*	6.09	2.29
LMOD1	6.51	-1.45	*PLXNC1*	5.48	3.86

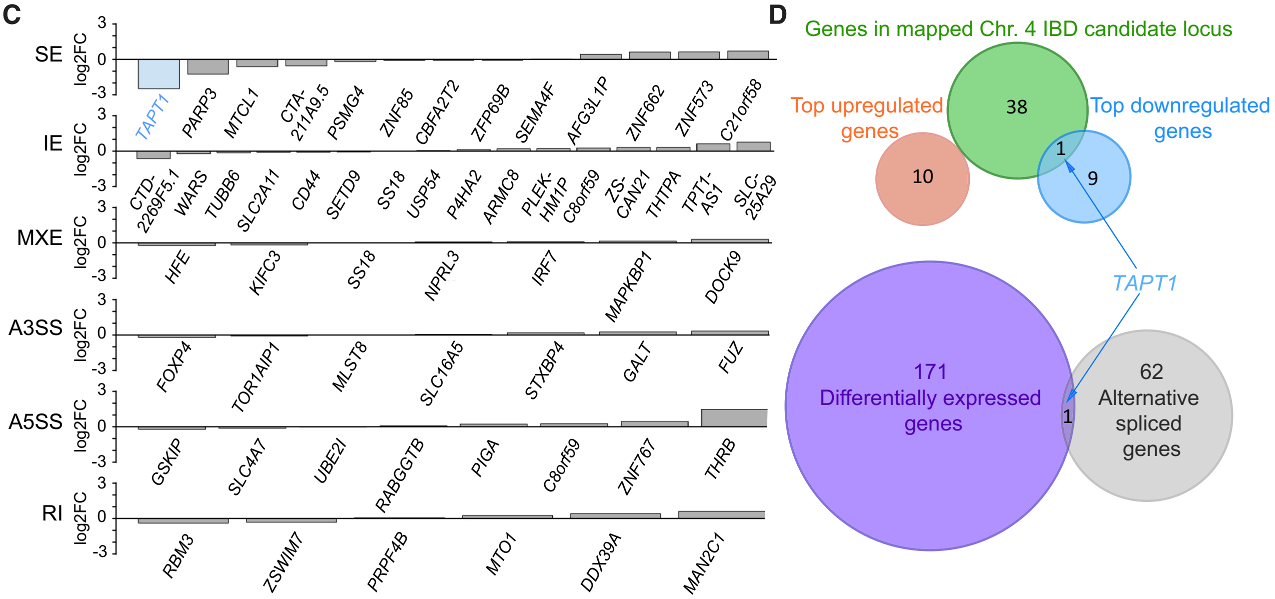

Figure EV2.

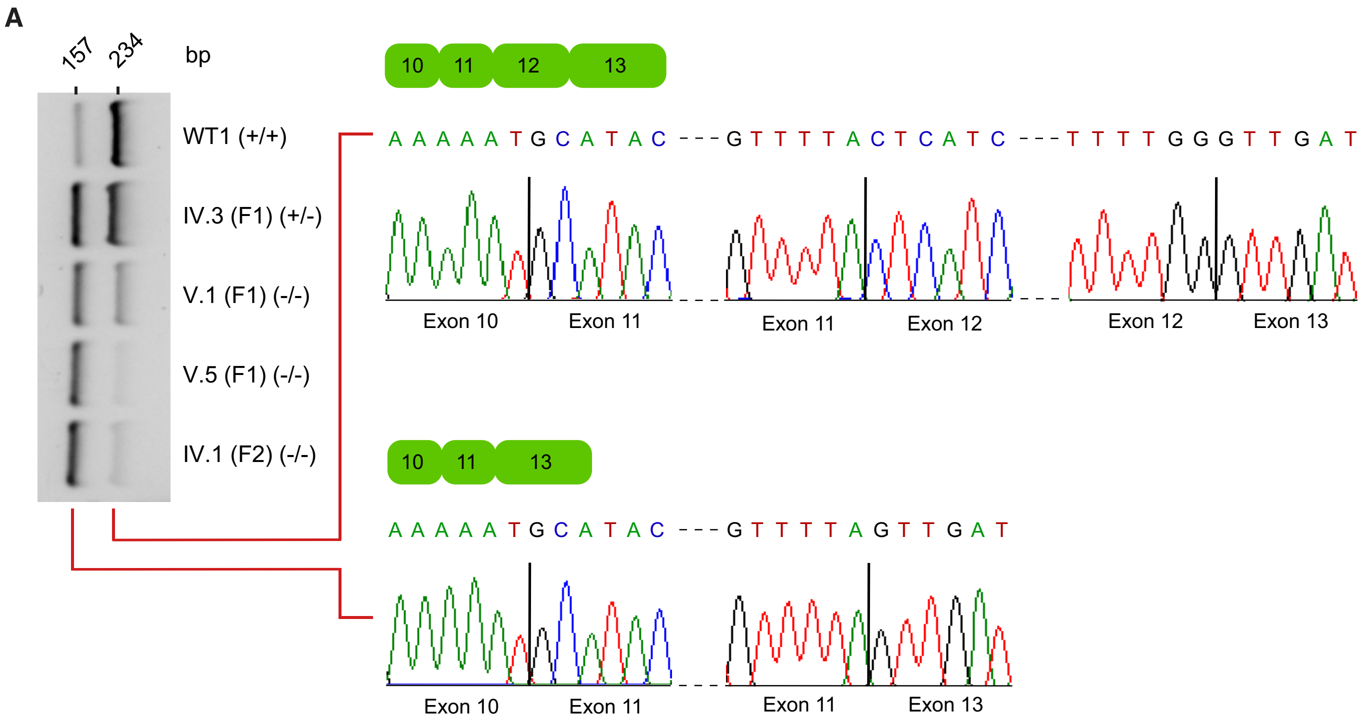

Figure EV3. Elevated numbers of exon-12 skipped transcripts in the patient cells.

A RT–PCR analysis of endogenous *TAPT1* splicing products. To check exon 12 skipping, RT–PCR was performed using primers targeting exon 10 and exon 13 in one WT (WT1), one heterozygous carrier (IV.3 (F1)) and 3 patients (V.1 (F1), V.5 (F1), IV.1 (F2)). The data showed the presence of normal (234 bps) and exon 12-skipped (157 bps) products in all tested samples. However, the truncated transcripts constitute the majority of products in the patient cells. Interestingly, the intensity of 2 bands is rather same in the heterozygous (IV.3 (F1)) sample. Sanger sequencing confirmed the accuracy of RT–PCR products.

Source data are available online for this figure.

Figure EV4. *TAPT1* c.1237-52 G>A mutation and *TAPT1-AS1* show no observable regulatory activity on *TAPT1* mRNA stability and gene expression, respectively.

A qPCR analysis of *c-MYC* and *TAPT1* expression in WT1 and V.1 (F1) primary fibroblasts treated with actinomycin D (ActD) in different time points. ActD was used to check mRNA stability by inhibiting transcription. *c-MYC* was considered as positive control with a short half-life. The results showed that *c-MYC* mRNA level dramatically decreased after 1.5 h treatment (~ x2), whereas the *TAPT1* transcript level is unchanged. qPCR assays involved three technical replicates per sample per time point. For each graph, fold change relative to non-treated condition is plotted as mean ± SD. Asterisks indicate conventional statistical significance (Student's *t*-test; n.s. *P*-value > 0.05, *****P*-value < 0.0001).

B Knockdown of *TAPT1-AS1* transcript using two different GapmeRs (1 and 2) in WT (WT1) and patient (IV.1 (F2)) primary dermal fibroblasts. A non-targeted (NT) GapmeR was used as control. qPCR analysis of *TAPT1-AS1* (top) and *TAPT1* (bottom) transcript levels in the GapmeR-transfected cells. Results show the successful knockdown of *TAPT1-AS1* by both GapmeRs 1 and 2 compared with the control NT GapmeR. However, *TAPT1* mRNA levels are unaltered in both WT and patient cells. Fold change relative to WT1-Control NT GapmeR is plotted as mean ± SD of three technical replicates. Asterisks indicate conventional statistical significance (Student's *t*-test; n.s. *P*-value > 0.05, ***P*-value < 0.01, ****P*-value < 0.001).

C Western blotting of protein extracts from the GapmeR-transfected cells, probing for TAPT1 (Sigma, HPA042567 antibody). Data shows that TAPT1 protein levels are unaffected by the knockdown of *TAPT1-AS1*. GAPDH was used as loading control.

D, E Immunofluorescence staining using two different TAPT1 commercial antibodies (A: Sigma, HPA042567; B: Sigma, HPA048658) in WT1 and IV.1 (F2) primary dermal fibroblasts. Similar fluorescent signal was detected in WT and TAPT1-null cells in both cases. TAPT1 commercial antibodies are unsuitable for immunofluorescence experiments. Scale bar represents 10 µm.

Source data are available online for this figure.

 © 2023 The Authors

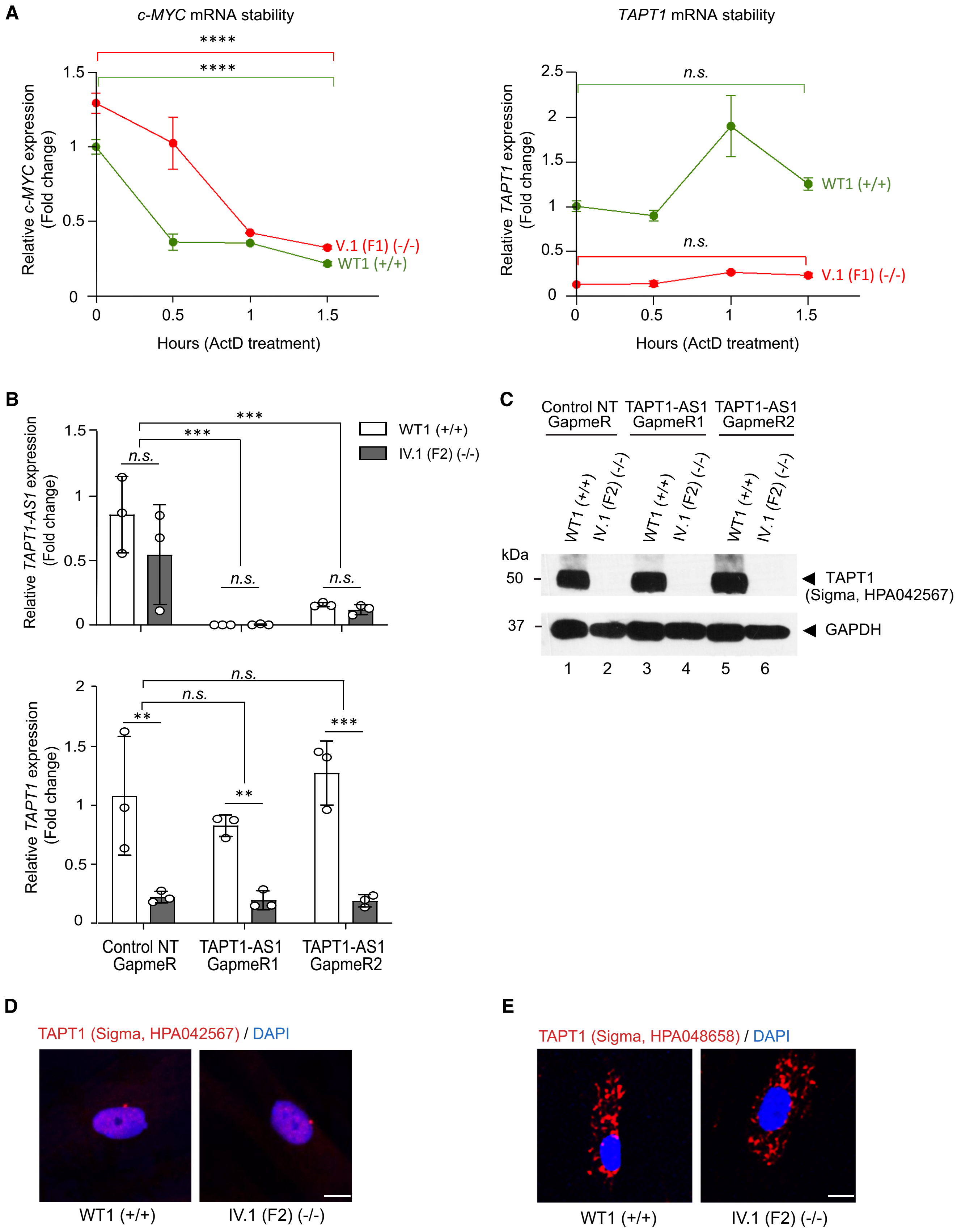
A
c-MYC mRNA stability
****
****
Relative c-MYC expression (Fold change)
V.1 (F1) (-/-)
WT1 (+/+)
Hours (ActD treatment)
TAPT1 mRNA stability
n.s.
n.s.
Relative TAPT1 expression (Fold change)
WT1 (+/+)
V.1 (F1) (-/-)
Hours (ActD treatment)
B
WT1 (+/+)
IV.1 (F2) (-/-)
Relative TAPT1-AS1 expression (Fold change)
Relative TAPT1 expression (Fold change)
Control NT GapmeR
TAPT1-AS1 GapmeR1
TAPT1-AS1 GapmeR2
C
Control NT GapmeR
TAPT1-AS1 GapmeR1
TAPT1-AS1 GapmeR2
WT1 (+/+)
IV.1 (F2) (-/-)
kDa
50
37
TAPT1 (Sigma, HPA042567)
GAPDH
1 2 3 4 5 6
D
TAPT1 (Sigma, HPA042567) / DAPI
WT1 (+/+)
IV.1 (F2) (-/-)
E
TAPT1 (Sigma, HPA048658) / DAPI
WT1 (+/+)
IV.1 (F2) (-/-)

Figure EV4.

Figure EV5. SI-NET-seq analysis data.

A Lists of the top 10 genes with significantly decreased (left, blue) or increased (right, red) RNA Pol II occupancy from our SI-NET-seq analysis.

B High Pearson's correlation coefficients ($r \geq 0.96$) between replicates of Pol II gene occupancy indicate the reproducibility of SI-NET-seq measurements. Asterisks indicate conventional statistical significance (Student's *t*-test; ****P*-value < 0.001).

C Volcano plot showing genes with an altered occupancy of transcriptionally engaged Pol II in the heterozygous parent (IV.3 (F1)) compared with WT (WT1 and WT2) primary fibroblast cells. The y-axis shows the $-\log10$ *P*-value, whereas the x-axis indicates the log2 fold change value for the Pol II occupancy. The Pol II density is increased in 149 genes (red dots) and decreased in 21 genes (blue dots). The yellow dot represents *TAPT1*.

D Bubble plot showing enrichment of collagen and extracellular matrix (ECM) pathways from the integrated Reactome pathway analysis from patients (red circles) and heterozygous parent (green circles) SI-NET-seq data. Enriched pathways are indicated on the y-axis, and the corresponding *P*-values are shown on the x-axis. The size of the circles represents the number of altered genes from each pathway.

E Pol II occupancy changes (log) of genes associated with enriched collagen and extracellular matrix (ECM) pathways measured from patients (−/−) and heterozygous parent (+/−) SI-NET-seq data. Significant changes are highlighted (orange). Bold and underlined genes are shared between patients and heterozygous parent.

Source data are available online for this figure.

 © 2023 The Authors

A

List of top genes with decreased RNA Pol II occupancy		
**Gene name**	**-log10 (padj)**	**Log2 fold change**
ENOPH1	41.9	-4.96
COMP	27.55	-4.29
CRLF1	16.86	-5.11
PTPRE	13.98	-4
ANGPTL4	12.1	-3.15
COL15A1	12.04	-2.36
CLEC2A	11.94	-5.46
MMP1	11.55	-4.92
PTGS1	11.15	-3.38
SHISAL1	11.12	-2.72

List of top genes with increased RNA Pol II occupancy		
**Gene name**	**-log10 (padj)**	**Log2 fold change**
INHBA	12.69	2.74
CEMIP	12.4	2.64
NR2F1-AS1	12.04	2.91
ADGRD1	10.51	2.91
NTNG1	10.25	3.18
LNX1	9.86	2.1
PTGS2	9.3	2.69
SSC5D	9.04	2.53
KCND3	8.93	3.03
TRPC4	8.44	3.29

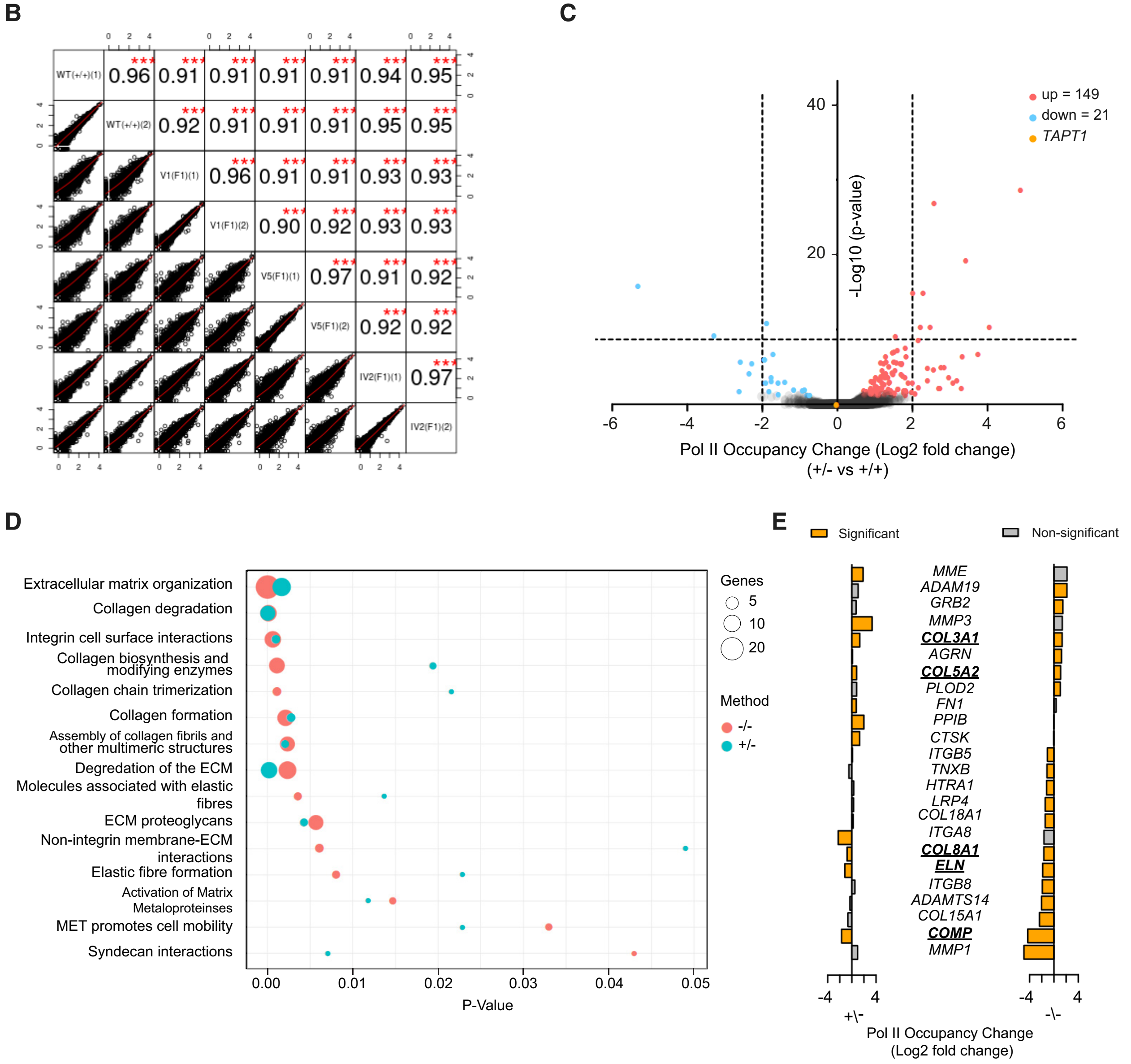

Figure EV5.
