# Supplementary material for: A progeroid syndrome caused by a deep intronic variant in TAPT1 is revealed by RNA/SI‐NET sequencing
Source: EMBO Mol Med. 2023 Jan 18;15(2):e16478. doi: 10.15252/emmm.202216478 (PMC9906387; doi:10.15252/emmm.202216478)
Supplement: Supplementary file 11 [file EMMM-15-e16478-s004.pdf]

EMBO Molecular Medicine

A progeroid syndrome caused by a deep intronic variant in TAPT1 is revealed by RNA/SI-NET sequencing

Nasrinsadat Nabavizadeh, Annkatrin Bressin, Mohammad Shboul, Ricardo Moreno Traspas, Poh Hui Chia, Carine Bonnard, Emmanuelle Szenker-Ravi, Burak Sarıbaş, Emmanuel Beillard, Umut Altunoglu, Zohreh Hojati, Scott Drutman, Susanne Freier, Mohammad El-Khateeb, Rajaa Fathallah, Jean-Laurent Casanova, Wesam Soror, Alaa Arafat, Nathalie Escande-Beillard, Andreas Mayer, and Bruno REVERSADE
DOI: 10.15252/emmm.202216478

Corresponding authors: Bruno REVERSADE (bruno@reversade.com) , Andreas Mayer (mayer@molgen.mpg.de)

Review Timeline:

Submission Date:	21st Jun 22
Editorial Decision:	15th Jul 22
Revision Received:	25th Nov 22
Editorial Decision:	1st Dec 22
Revision Received:	13th Dec 22
Accepted:	14th Dec 22

Editor: Zeljko Durdevic

Transaction Report:

(Note: With the exception of the correction of typographical or spelling errors that could be a source of ambiguity, letters and reports are not edited. Depending on transfer agreements, referee reports obtained elsewhere may or may not be included in this compilation. Referee reports are anonymous unless the Referee chooses to sign their reports.)

15th Jul 2022

Dear Prof. Reversade,

Thank you for the submission of your manuscript to EMBO Molecular Medicine. We have now received feedback from the two reviewers who agreed to evaluate your manuscript. As you will see from the reports below, while the referee #1 is overall supportive of the study, referee #2 recognizes potential interest of the study but also raises important concerns that should be addressed in a major revision.

Further consideration of a revision that addresses reviewers' concerns in full will entail a second round of review. EMBO Molecular Medicine encourages a single round of revision only and therefore, acceptance or rejection of the manuscript will depend on the completeness of your responses included in the next, final version of the manuscript. For this reason, and to save you from any frustrations in the end, I would strongly advise against returning an incomplete revision.

We would welcome the submission of a revised version within three months for further consideration. Please let us know if you require longer to complete the revision.

Please use this link to login to the manuscript system and submit your revision: https://embomolmed.msubmit.net/cgi-bin/main.plex

I look forward to receiving your revised manuscript.

Yours sincerely,

Zeljko Durdevic

Zeljko Durdevic
Editor
EMBO Molecular Medicine

***** Reviewer's comments *****

Referee #1 (Remarks for Author):

** Manuscript No.: EMM-2022-16478 **

Title: A progeroid syndrome caused by a TAPT1 mutation is revealed by combined RNA/SI-NET sequencing

Authors: Nasrinsadat Nabavizadeh, Annkatrin Bressin, Poh Hui Chia, Ricardo Moreno Traspas, Nathalie Escande-Beillard, Carine Bonnard, Zohreh Hojati, Scott Drutman, Susanne Freier, Mohammad El Khateeb, Rajaa Fathallah, Jean-Laurent Casanova, Wesam Soror, Alaa Arafat, Mohammad Shboul, Andreas Mayer, and Bruno REVERSADE

report:
Nabavizeadeh et al. report on two distantly related consanguineous families with in total six affected family members with severe bone defects, developmental delay and premature aging. The authors identified a deep intronic homozygous variant in the known disease gene TAPT1 as the causal mutation.
This is not a new-gene identification study, as similar phenotypes have been described before. I would like to point to two significant achievements:

1. An advanced genomic work-up that included WES, homozygosity mapping, RNAseq and Sanger sequencing identified an extremely rare homozygeous deep intronic variant, that segregated within the families. Prediction analysis suggested alternative splicing, and exon 12 skipping leading to NMD was further validated by qPCR, Western blots, and immunohistochemistry. Si-Net-seq proved that nascent transcription was not affected. This is a nice success story of alternative genetic diagnostics covering apparently functional relevant loci not accessible with common gene panel and WES analysis.
2. After validation, patient fibroblasts have been used for functional analysis including amongst others pathway analysis by RNAseq and Si-Net-seq pointing at Collagen- and ECM-related pathways and Western blot for cellular localization both revealing insights in TAPT1 biological function.
The claims/conclusions are convincing and appropriately discussed, the project well designed and covers all necessary experiments with adequate techniques and design (e.g. replicates, statistics). The study is therefore of importance and relevant to clinical geneticists and colleagues involved in molecular diagnostics. Actually, I enjoyed reading.

• major comments
no major concerns,
• minor comments
1 references: some are not assigned to correctly, e.g. page 6 (MIM616897) should be 15 and not 14. In the reference list, information is sometimes missing like journal name, formatting is not consistent.
The manuscript is both in style and content professionally done, there are no additional comments.

Referee #2 (Comments on Novelty/Model System for Author):

Adequate

Referee #2 (Remarks for Author):

In the manuscript, Nabavizadeh and colleagues by integrating homozygosity mapping and RNA-seq approaches, identified an intronic mutation (upstream of exon 12) in the TAPT1 gene of six patients with a recessive osteogenesis imperfecta and neonatal progeria syndrome. These authors suggested that this mutation promotes skipping of exon 12 in the TAPT1 mRNA.

This altered alternative splicing event could introduce a premature stop codon thus generating an instable transcript that is degraded through non sense-mediated mRNA decay (NMD) pathway. In accord with this hypothesis, they found reduced expression of the TAPT1 gene (at both mRNA and proteins levels) in patients' fibroblasts compared to normal fibroblast cells. In addition, they identified a number of changes in mRNA steady-state levels in patients' fibroblasts that implicate alteration of the extracellular matrix and collagen-related pathways. This is an interesting work, but additional experiments must be done by the authors for supporting their conclusions. In particular, there are some aspects related to alternative splicing coupled to nonsense-mediated decay (AS-NMD) as well as the lack of a molecular mechanism that must be addressed by the authors in order to set the story on firmer ground.

Specific Comment:

1) These authors must demonstrate that the reduced expression of the TAPT1 gene (at both mRNA and proteins levels) is due to the activation of an AS-NMD program. In order to do this, they need to block NMD for example by using cycloheximide (a strong NMD inhibitor) and analyzed total TAPT1 expression by qPCR and wester blotting in patients' fibroblasts; since cycloheximide could cause pleiotropic effects, better is to perform siRNA-mediated knockdown of a key NMD player (for example Upf1).
2) Splicing analysis data (Fig 2D) must be confirm through RT-PCR with primers in exon 11 and 13 in order to detect exon 12 skipping in patients' fibroblasts compared to normal fibroblasts.
3) In vitro splicing assay with a TAPT1 minigene (wt and mutated) should be perform to demonstrated that the c.1237-52G>A is the determinant that sustains skipping of TAPT1 exon 12.
4) By interrogating public datasets (for example https://www.proteinatlas.org/) these author could analyze TAPT1 mRNA expression levels in different normal human tissues and determine if this gene is more expressed in the most affected tissues of the patients.
5) By comparing through RNA-seq the transcriptome of normal vs patients' fibroblasts, these authors identified a number of changes in mRNA steady-state levels. Interestingly, GO analysis showed a significant enrichment of collagen and extracellular matrix and collagen-related pathways. Are these changes also observed in normal fibroblasts upon TAPT1 knockdown?
6) However, it remains unclear the molecular mechanism through which the absence of TAPT1 causes changes in the expression levels of genes, some of them (for example genes encoding for collagen and extracellular matrix) are important to explain phenotypical features of the patients. This is an interesting study that will benefit from addressing this important point.

Point-by-point Response to Reviewers' Comments

Reviewer #1:

Nabavizeadeh et al. report on two distantly related consanguineous families with in total six affected family members with severe bone defects, developmental delay and premature aging. The authors identified a deep intronic homozygous variant in the known disease gene TAPT1 as the causal mutation.
This is not a new-gene identification study, as similar phenotypes have been described before. I would like to point to two significant achievements:
1. An advanced genomic work-up that included WES, homozygosity mapping, RNAseq and Sanger sequencing identified an extremely rare homozygous deep intronic variant, that segregated within the families. Prediction analysis suggested alternative splicing, and exon 12 skipping leading to NMD was further validated by qPCR, Western blots, and immunohistochemistry. Si-Net-seq proved that nascent transcription was not affected. This is a nice success story of alternative genetic diagnostics covering apparently functional relevant loci not accessible with common gene panel and WES analysis.
2. After validation, patient fibroblasts have been used for functional analysis including amongst others pathway analysis by RNAseq and Si-Net-seq pointing at Collagen- and ECM-related pathways and Western blot for cellular localization both revealing insights in TAPT1 biological function.
The claims/conclusions are convincing and appropriately discussed, the project well designed and covers all necessary experiments with adequate techniques and design (e.g. replicates, statistics). The study is therefore of importance and relevant to clinical geneticists and colleagues involved in molecular diagnostics. Actually, I enjoyed reading.
• major comments
no major concerns,
• minor comments
1 references: some are not assigned to correctly, e.g. page 6 (MIM616897) should be 15 and not 14. In the reference list, information is sometimes missing like journal name, formatting is not consistent.
The manuscript is both in style and content professionally done, there are no additional comments.

We thank the reviewer for his/her interest in our study and the positive feedback. We have checked and corrected all the reference numbers. All the references are now formatted according to the journal style.

Reviewer #2:

1) These authors must demonstrate that the reduced expression of the TAPT1 gene (at both mRNA and protein levels) is due to the activation of an AS-NMD program. In order to do this, they need to block NMD for example by using cycloheximide (a strong NMD inhibitor) and analyzed total TAPT1 expression by qPCR and western blotting in patients' fibroblasts; since cycloheximide could cause pleiotropic effects, better is to perform siRNA-mediated knockdown of a key NMD player (for example Upf1).

Thank you for pointing this out. To check whether a reduced amount of *TAPT1* in the patient cells is due to nonsense mediated decay (NMD), we treated patient and WT fibroblast cells with the NMD inhibitor cycloheximide (CHX) for 4 and 8 hours. DMSO was used as a negative vehicle control. As expected, *TAPT1* transcript levels significantly increased (approximately 4 fold) over time in the mutant cells whereas it remained unchanged in the WT cells. This data was confirmed in 3 biological and 3 technical replicates (see **new Fig. 4D**).

Conversely, we could not observe TAPT1 protein rescue in the CHX-treated patient cells by western blot (see reviewer Figure 1 below). The discrepancy observed betweenRNA and protein TAPT1 rescue might be explained either by: 1) The impossibility of the mis-spliced *TAPT1* transcripts to be translated into a proper protein. 2) The inability of the TAPT1 antibodies to detect the truncated protein or 3) the implication of additional regulatory mechanisms for degradation of misfolded proteins. We note that exon12 encodes part of the 4th and 5th predicted transmembrane helices, without which TAPT1 might not be properly inserted into the ER/Golgi and undergo targeted degradation.

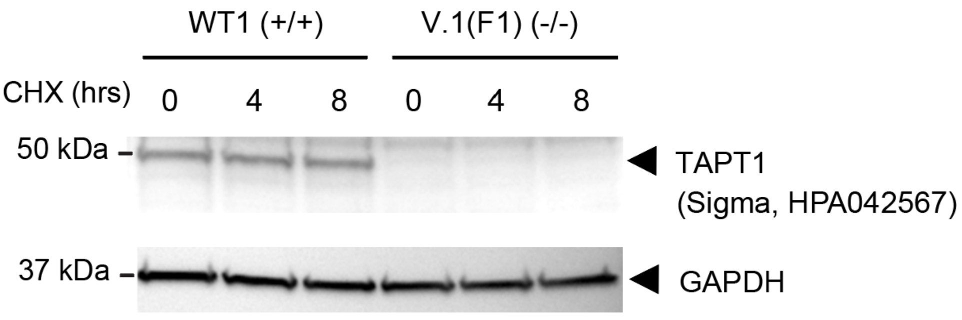

Reviewer Figure 1. Western blot analysis showing endogenous TAPT1 levels in WT1 (+/+) and V.1 (F1) (-/-) fibroblasts treated with CHX for 4 and 8 hours. DMSO was used as a negative vehicle control.

Moreover, we examined the effect of c.1237-52 G>A mutation on *TAPT1* RNA stability. Patient and WT cells were treated with Actinomycin D (ActD), an established transcription inhibitor which intercalates into DNA. The level of *TAPT1* transcripts along with those of *c-MYC* were assessed by qPCR. The results showed that the *c-MYC* RNA level significantly decreased (more than 2 fold) after 1.5 hour ActD treatment in patient and WT cells whereas *TAPT1* transcripts remained stable during the treatment in both patient and WT cells (see **new Fig. EV4A**).

Overall, our results suggest that this private *TAPT1* mutation (c.1237-52 G>A) triggers *TAPT1* mRNA degradation through the classical NMD pathway but does not significantly affect the intrinsic stability of *TAPT1* transcripts.

2) Splicing analysis data (Fig 2D) must be confirmed through RT-PCR with primers in exon 11 and 13 in order to detect exon 12 skipping in patients' fibroblasts compared to normal fibroblasts.

To check the occurrence of exon 12 skipping, we performed RT-PCR using primers targeting exon 10 and 13 of *TAPT1* for WT, heterozygous carrier and 3 independent patients. The position of primers are shown for your reference in reviewer Figure 2 below. We provide evidence that exon 12 skipping occurs in all tested samples including WT, heterozygous carrier and patients. However, the private mutation aggravates the aberrant splicing event in all 3 patients compared to WT. Interestingly, for the heterozygous mother we detected bands corresponding to normal (157 bp) and exon 12 skipped (234 bp) splicing products with equal intensities reflecting her genotype (see **new Fig. EV3A**). Moreover, direct Sanger sequencing which was performed on each RT-PCR product, confirmed the sequences of WT and exon 12 skipped transcripts (see **new Fig. EV3A**).

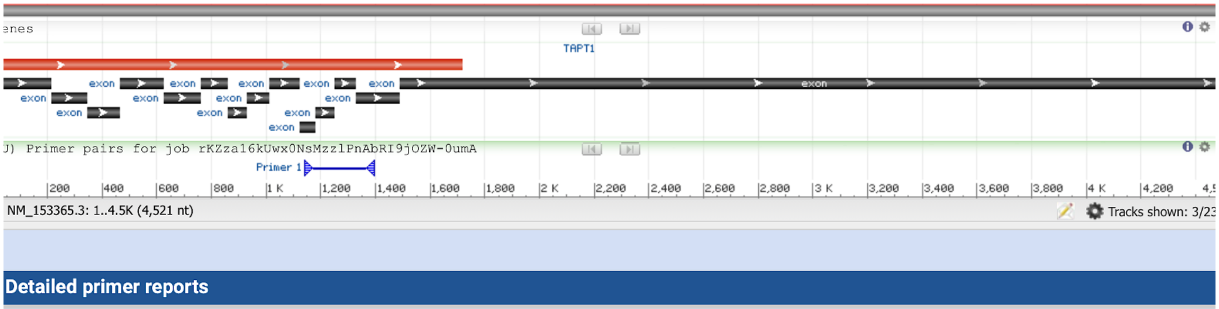

	Sequence (5'->3')	Template strand	Length	Start	Stop	Tm	GC%	Self complementarity	Self 3' complementarity
Forward primer	TTTGACCTTGTTAGCAGCCG	Plus	20	1152	1171	58.77	50.00	4.00	2.00
Reverse primer	ATACTGGCACGATTTCCCCA	Minus	20	1385	1366	59.08	50.00	3.00	0.00
Product length	234								

Products on intended targets

>NM_153365.3 Homo sapiens transmembrane anterior posterior transformation 1 (TAPT1), mRNA

Reviewer Figure 2. Schematic representation of the genomic location and sequence of RT-PCR forward and reverse primers targeting *TAPT1* exon 10 and exon 13.

3) In vitro splicing assay with a TAPT1 minigene (wt and mutated) should be performed to demonstrate that the c.1237-52G>A is the determinant that sustains skipping of TAPT1 exon 12.

Your suggestion has greatly improved the strength of claims allowing to confirm the causality of our deep intronic mutation. Following your advice, we adapted and performed a minigene splicing assay using the pSPL3 trapping vector (Westin *et al,* 2021, Scientific Report). *TAPT1* exon 12 and its adjacent intronic sequences covering 200 bp upstream (encompassing the c.1237-52 G>A mutation) and 500 bps downstream of the exon 12 regions were cloned from the patient's gDNA into the pSPL3. We also generated a rescue construct by directly reversing the mutant allele to WT (c.1237-52 A>G) using site directed mutagenesis. A schematic view of the constructs' design is included in the **new Fig. 3C**.

HEK293T cells were transfected with both constructs along with empty pSPL3 as a control. Following RNA extraction and cDNA synthesis, the splicing products were monitored by RT-PCR using SD6 and SA2 primers. These primers target the 2 exons in the backbone of the vector (expected size = 263 bp). Our data showed that the c.1237-52 A>G variant resulted mainly in a truncated PCR product (263 bp), due to exon 12 skipping whereas for the rescue construct a major RT-PCR product containing exon 12 (340 bps) was detected instead (see **new Fig. 3C**). Additionally, targeted Sanger sequencing of the RT-PCR products confirmed the validity of the normal and truncated transcripts.

Importantly, the findings of this *in vitro* splicing assay are consistent with our previous results obtained by RT-PCR on endogenous *TAPT1* transcripts (see **new Fig. EV3A**).

4) By interrogating public datasets (for example https://www.proteinatlas.org/) these authors could analyze TAPT1 mRNA expression levels in different normal human tissues and determine if this gene is more expressed in the most affected tissues of the patients.

Thank you for pointing this out. We have examined *TAPT1* expression using the Human Protein Atlas and GTEx and show the data in **Reviewer Figure 3**. *TAPT1* being ubiquitously expressed, we could not find meaningful correlation between transcript levels and patient's affected tissues such as skin, bone or adipose tissue. Moreover, *TAPT1* is lowly expressed (nTPM <40) as compared to other ubiquitously expressed genes such as *GAPDH* and *b-ACTIN* (nTPM >400).

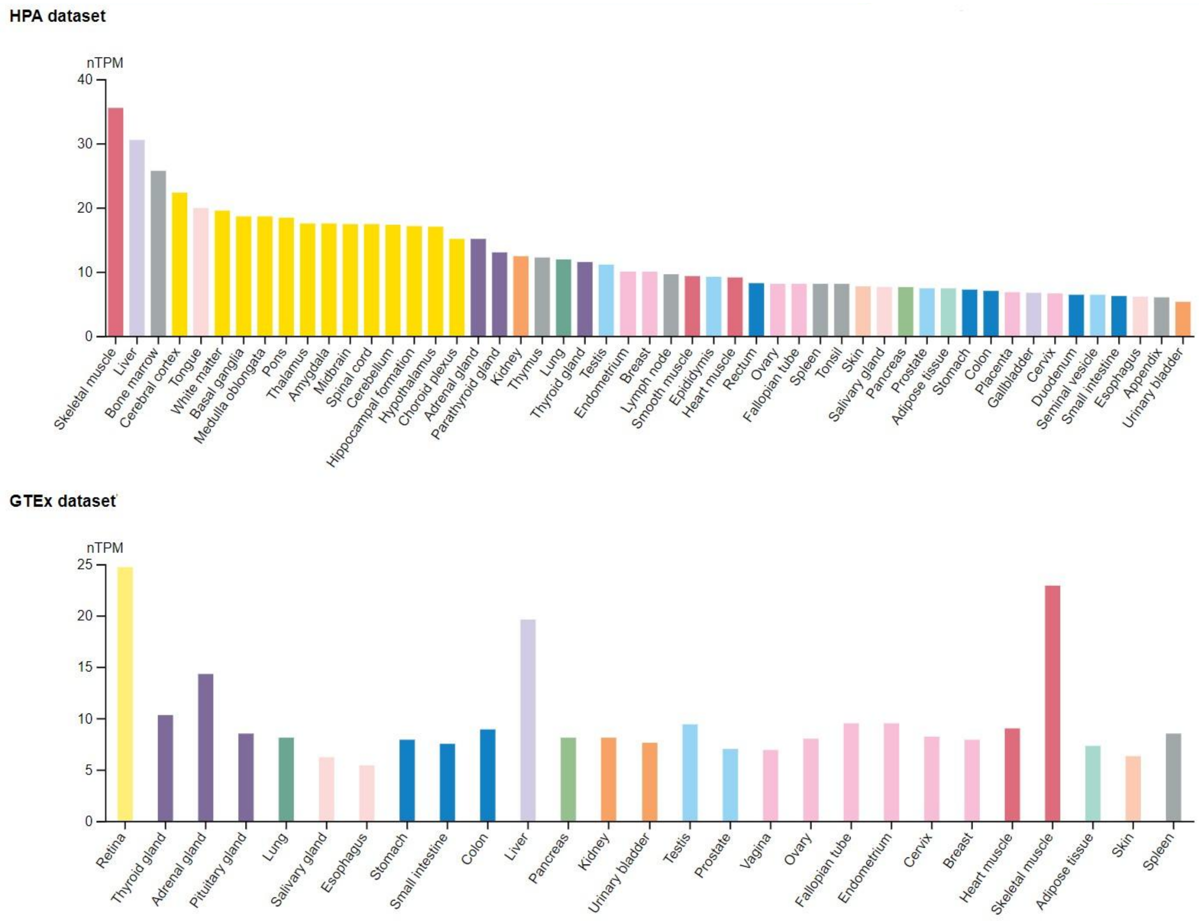

Reviewer Figure 3: Overview of *TAPT1* expression in different tissues as retrieved from the human protein atlas (top panel) (HPA) and GTEX (lower panel). nTPM = number of transcripts per million.

It is not uncommon to witness in other Mendelian disorders that the most severely affected tissues are not those that express the highest levels of expression of the culprit gene. For example, we previously showed that loss of function mutations in *PYCR1* can cause cutis laxa, a disease characterized by loose and wrinkled skin, while this gene is very lowly expressed in skin tissue (Reversade *et al*, 2009, Nature Genetics). Recently, we revealed that *FOCAD* mutations can cause severe inherited liver cirrhosis (Moreno Traspas *et al,* 2022, Nature Genetics) while the liver showed the lowest *FOCAD* expression among all tested tissues.

5) By comparing through RNA-seq the transcriptome of normal vs patients' fibroblasts, these authors identified a number of changes in mRNA steady-state levels. Interestingly, GO analysis showed a significant enrichment of collagen and extracellular matrix and collagen-related pathways. Are these changes also observed in normal fibroblasts upon TAPT1 knockdown?

We thank the reviewer for bringing this up. Indeed the affected pathways and their association with collagen and the ECM were compelling in the patients' fibroblast cells (Fig. 6D).

This study identified a homozygous (HMZ) splicing mutation in *TAPT1* as the likely cause of the disease associated with a complete TAPT1 protein loss (Fig. 2D, 4C). Consistent with this finding, TAPT1 protein levels were reduced by nearly 50% in fibroblast cells from a heterozygous mother (+/-) without clinical manifestation (IV.3 (F1)(+/-) (Fig. 4C). Here, we also showed this robust decrease at the RNA and protein level (**Reviewer Figure 4** below), suggesting that HTZ (+/-) fibroblast cells represent a natural TAPT1 knockdown as compared to the complete TAPT1 loss observed in patient cells (-/-).

Heeding your advice, we now applied SI-NET-seq to the HTZ (+/-) fibroblast cells. SI-NET-seq quantitatively measures nascent transcriptional changes and identifies affected pathways with higher sensitivity than standard RNA-seq analyses (Figure 6D). As expected, the new data shows transcriptional changes at fewer genes in HTZ (TAPT1 knockdown) cells compared to the complete TAPT1 loss in patient cells (HTZ (+/-): 170 genes vs. HMZ (-/-): 317 genes (**new Fig. EV6C**, Fig. 6C and **Table S3)**. Surprisingly, an integrated *Reactome pathway* analysis of HTZ (+/-) and HMZ (-/-) fibroblast cells revealed a high overlap of affected pathways and a clear link to the disease phenotype (see **new Fig. EV6D**). However, considering the 24 deregulated genes associated with the pathogenic pathways shown in **new Fig. EV6E**, only transcription of five genes (*COMP*, *ELN*, *COL8A1*, *COL5A2*, *COL3A1*) were significantly changed in both HMZ (-/-) and HTZ (+/-) cells. The remaining deregulated genes identified in HMZ cells were not altered in HTZ (+/-) cells, as depicted in the **new Fig. EV6E**. Instead, we observed the activation of a distinct group of genes (*CTSK*, *PPIB*, *FN1*, *MMP3*, *MME*) associated with collagen-and ECM-related pathways.

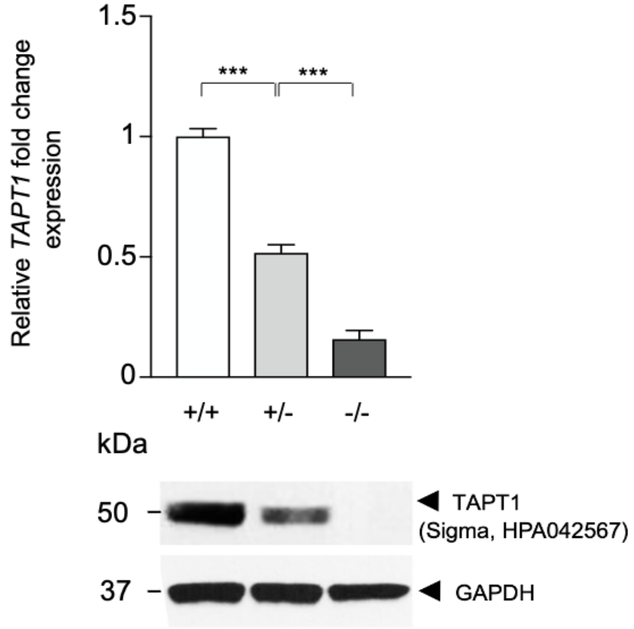

Reviewer Figure 4: qPCR (top panel) and western blot (lower panel) analyses showing endogenous RNA and protein TAPT1 levels in WT (+/+), heterozygous carrier (+/-) and patient (-/-). The results indicate intermediate RNA and protein levels of TAPT1 in the heterozygous carrier (+/-) compared to WT (+/+) and patient (-/-). Fold change relative to +/+ is plotted as mean ± SD. Asterisks indicate statistical significance (Student t-test; *** p-value < 0.001)

This new data indicate a partial molecular phenotype of HTZ (+/-) compared to HMZ (-/-) fibroblast cells. This finding supports the view that the level of deregulation in HTZ (+/-) cells did not exceed the critical level, as no clinical phenotype manifested in HTZ (+/-) parents. Additionally, the activation of genes involved in collagen- and ECM-related pathways argues for putative compensation effects in HTZ (+/-) cells. Raw sequencing data and processed files have been deposited in the Gene Expression Omnibus (GEO) database under the following accession number: GSE197120.

6) However, it remains unclear the molecular mechanism through which the absence of TAPT1 causes changes in the expression levels of genes, some of them (for example genes encoding for collagen and extracellular matrix) are important to explain phenotypical features of the patients. This is an interesting study that will benefit from addressing this important point.

Deciphering the molecular function of TAPT1 is beyond the scope of this article. Here we are aiming to showcase the use of RNA-seq/SI-NET-seq as a powerful means to identify the cause of orphan diseases with non-coding mutations.

We have speculated at length in the discussion what may be the pathogenesis of TAPT1-deficiency. While it appears as a collagenopathy in humans, it cannot be the case since the homologous gene in plants, fungi and plasmodium which do not produce collagen, is also essential for basic organismal function.

We can confidently say that it does not cause ER stress, is unlikely to play a role in ciliogenesis, is not a receptor for CMV, and that it must interact directly with SUCO to carry out its cellular function. Having 5 transmembrane helices, we can only speculate that this protein probably acts as a transporter or a receptor mediating fundamental cellular processes that are common to all eukaryotic cells.

Be assured that we will be working hard in the coming years to unravel this lingering mystery.

1st Dec 2022

Dear Prof. Reversade,

Thank you for the submission of your revised manuscript to EMBO Molecular Medicine. I am pleased to inform you that we will be able to accept your manuscript pending the following final amendments:

1) Figures:
- Please reduce number of EV Figure to 5 e,g, by merging EV Figure 4 and 5. Please check our Author Guidelines: https://www.embopress.org/page/journal/17574684/authorguide#expandedview
- Figure 6B shows qPCR validation of dysregulated genes on n=2. Please show raw values from both measurements and remove error bars. When n=2 statistical analysis is not recommended and a justification for the use of the statistical test employed has to be provided. Please check our Author Guidelines: https://www.embopress.org/page/journal/17574684/authorguide#statisticalanalysis
2) In the main manuscript file, please do the following:
- In M&M, add statistical paragraph that should reflect all information that you have filled in the Authors Checklist, especially regarding randomization, blinding, replication.
- Data availability: In addition to the accession number please provide URL for deposited datasets. Please be aware that all datasets should be made freely available upon acceptance, without restriction. Use the following format to report the accession number of your data:

The datasets produced in this study are available in the following databases:
[data type]: [full name of the resource] [accession number/identifier] ([doi or URL or identifiers.org/DATABASE:ACCESSION])

Please check "Author Guidelines" for more information.
https://www.embopress.org/page/journal/17574684/authorguide#availabilityofpublishedmaterial
3) Synopsis:
- Synopsis image: Please resize the visual abstract to 550 px-wide x (250-400)-px high and submit as a high-resolution jpeg file.
- Please check your synopsis text and image before submission with your revised manuscript. Please be aware that in the proof stage minor corrections only are allowed (e.g., typos).
4) For more information: This space should be used to list relevant web links for further consultation by our readers. Could you identify some relevant ones and provide such information as well? Some examples are patient associations, relevant databases, OMIM/proteins/genes links, author's websites, etc...
5) As part of the EMBO Publications transparent editorial process initiative (see our Editorial at http://embomolmed.embopress.org/content/2/9/329), EMBO Molecular Medicine will publish online a Review Process File (RPF) to accompany accepted manuscripts. This file will be published in conjunction with your paper and will include the anonymous referee reports, your point-by-point response and all pertinent correspondence relating to the manuscript. Let us know whether you agree with the publication of the RPF and as here, if you want to remove or not any figures from it prior to publication. Please note that the Authors checklist will be published at the end of the RPF.
6) Please provide a point-by-point letter INCLUDING my comments as well as the reviewer's reports and your detailed responses (as Word file).

I look forward to reading a new revised version of your manuscript as soon as possible.

Yours sincerely,

Zeljko Durdevic

Zeljko Durdevic
Editor
EMBO Molecular Medicine

***** Reviewer's comments *****

Referee #2 (Remarks for Author):

Dear Editor,

The authors performed most of the work that I requested and the manuscript text has been modified according to the reviewers' suggestions. Therefore, I have no additional reservation.

The authors addressed the minor editorial issues.

We are pleased to inform you that your manuscript is accepted for publication and is now being sent to our publisher to be included in the next available issue of EMBO Molecular Medicine.

EMBO Press Author Checklist

Corresponding Author Name: Bruno Reversade, Andreas Mayer	
Journal Submitted to: EMBO Molecular Medicine	
Manuscript Number: EMM-2022-16478	

USEFUL LINKS FOR COMPLETING THIS FORM
The EMBO Journal - Author Guidelines
EMBO Reports - Author Guidelines
Molecular Systems Biology - Author Guidelines
EMBO Molecular Medicine - Author Guidelines

Reporting Checklist for Life Science Articles (updated January 2022)
This checklist is adapted from Materials Design Analysis Reporting (MDAR) Checklist for Authors. MDAR establishes a minimum set of requirements in transparent reporting in the life sciences (see Statement of Task: 10.31222/osf.io/9sm4x). Please follow the journal's guidelines in preparing your manuscript.
Please note that a copy of this checklist will be published alongside your article.

Abridged guidelines for figures
1. Data
The data shown in figures should satisfy the following conditions:
- the data were obtained and processed according to the field's best practice and are presented to reflect the results of the experiments in an accurate and unbiased manner.
- ideally, figure panels should include only measurements that are directly comparable to each other and obtained with the same assay.
- plots include clearly labeled error bars for independent experiments and sample sizes. Unless justified, error bars should not be shown for technical replicates.
- if n<5, the individual data points from each experiment should be plotted. Any statistical test employed should be justified.
- Source Data should be included to report the data underlying figures according to the guidelines set out in the authorship guidelines on Data Presentation.

2. Captions
Each figure caption should contain the following information, for each panel where they are relevant:
- a specification of the experimental system investigated (eg cell line, species name).
- the assay(s) and method(s) used to carry out the reported observations and measurements.
- an explicit mention of the biological and chemical entity(ies) that are being measured.
- an explicit mention of the biological and chemical entity(ies) that are altered/varied/perturbed in a controlled manner.
- the exact sample size (n) for each experimental group/condition, given as a number, not a range;
- a description of the sample collection allowing the reader to understand whether the samples represent technical or biological replicates (including how many animals, litters, cultures, etc.).
- a statement of how many times the experiment shown was independently replicated in the laboratory.
- definitions of statistical methods and measures:
  - common tests, such as t-test (please specify whether paired vs. unpaired), simple χ2 tests, Wilcoxon and Mann-Whitney tests, can be unambiguously identified by name only, but more complex techniques should be described in the methods section;
  - are tests one-sided or two-sided?
  - are there adjustments for multiple comparisons?
  - exact statistical test results, e.g., P values = x but not P values < x;
  - definition of 'center values' as median or average;
  - definition of error bars as s.d. or s.e.m.

Please complete ALL of the questions below.
Select "Not Applicable" only when the requested information is not relevant for your study.

Materials

Newly Created Materials	Information included in the manuscript?	In which section is the information available? (Reagents and Tools Table, Materials and Methods, Figures, Data Availability Section)
New materials and reagents need to be available; do any restrictions apply?	Not Applicable	

Antibodies	Information included in the manuscript?	In which section is the information available? (Reagents and Tools Table, Materials and Methods, Figures, Data Availability Section)
For **antibodies** provide the following information:<br>- Commercial antibodies: RRID (if possible) or supplier name, catalogue number and or/clone number<br>- Non-commercial: RRID or citation	Yes	Materials and Methods

DNA and RNA sequences	Information included in the manuscript?	In which section is the information available? (Reagents and Tools Table, Materials and Methods, Figures, Data Availability Section)
**Short novel DNA or RNA including primers, probes**: provide the sequences.	Yes	Appendix table S4

Cell materials	Information included in the manuscript?	In which section is the information available? (Reagents and Tools Table, Materials and Methods, Figures, Data Availability Section)
**Cell lines:** Provide species information, strain. Provide accession number in repository **OR** supplier name, catalog number, clone number, and/**OR** RRID.	Yes	Material and Methods
**Primary cultures:** Provide species, strain, sex of origin, genetic modification status.	Yes	Material and Methods
Report if the cell lines were recently **authenticated** (e.g., by STR profiling) and tested for mycoplasma contamination.	Yes	Material and Methods

Experimental animals	Information included in the manuscript?	In which section is the information available? (Reagents and Tools Table, Materials and Methods, Figures, Data Availability Section)
**Laboratory animals or Model organisms:** Provide species, strain, sex, age, genetic modification status. Provide accession number in repository **OR** supplier name, catalog number, clone number, **OR** RRID.	Not Applicable	
**Animal observed in or captured from the field:** Provide species, sex, and age where possible.	Not Applicable	
Please detail **housing and husbandry conditions**.	Not Applicable	

Plants and microbes	Information included in the manuscript?	In which section is the information available? (Reagents and Tools Table, Materials and Methods, Figures, Data Availability Section)
**Plants:** provide species and strain, ecotype and cultivar where relevant, unique accession number if available, and source (including location for collected wild specimens).	Not Applicable	
**Microbes:** provide species and strain, unique accession number if available, and source.	Not Applicable	

Human research participants	Information included in the manuscript?	In which section is the information available? (Reagents and Tools Table, Materials and Methods, Figures, Data Availability Section)
If collected and within the bounds of privacy constraints report on age, sex and gender or ethnicity for all study participants.	Yes	Materials and Methods

Core facilities	Information included in the manuscript?	In which section is the information available? (Reagents and Tools Table, Materials and Methods, Figures, Data Availability Section)
If your work benefited from core facilities, was their service mentioned in the acknowledgments section?	Yes	Acknowledgments

Design

Study protocol	Information included in the manuscript?	In which section is the information available? (Reagents and Tools Table, Materials and Methods, Figures, Data Availability Section)
If study protocol has been **pre-registered, provide DOI in the manuscript**. For clinical trials, provide the trial registration number **OR** cite DOI.	Not Applicable	
Report the **clinical trial registration number** (at ClinicalTrials.gov or equivalent), where applicable.	Not Applicable	

Laboratory protocol	Information included in the manuscript?	In which section is the information available? (Reagents and Tools Table, Materials and Methods, Figures, Data Availability Section)
Provide DOI OR other citation details if **external detailed step-by-step protocols** are available.	Not Applicable	

Experimental study design and statistics	Information included in the manuscript?	In which section is the information available? (Reagents and Tools Table, Materials and Methods, Figures, Data Availability Section)
Include a statement about **sample size** estimate even if no statistical methods were used.	Yes	Materials and Methods
Were any steps taken to minimize the effects of subjective bias when allocating animals/samples to treatment (e.g. **randomization procedure**)? If yes, have they been described?	Not Applicable	
Include a statement about **blinding** even if no blinding was done.	Not Applicable	
Describe **inclusion/exclusion criteria** if samples or animals were excluded from the analysis. Were the criteria pre-established?<br><br>If sample or data points were omitted from analysis, report if this was due to attrition or intentional exclusion and provide justification.	Not Applicable	
For every figure, are **statistical tests** justified as appropriate? Do the data meet the assumptions of the tests (e.g., normal distribution)? Describe any methods used to assess it. Is there an estimate of variation within each group of data? Is the variance similar between the groups that are being statistically compared?	Yes	Figures

Sample definition and in-laboratory replication	Information included in the manuscript?	In which section is the information available? (Reagents and Tools Table, Materials and Methods, Figures, Data Availability Section)
In the figure legends: state number of times the experiment was **replicated** in laboratory.	Yes	Figures
In the figure legends: define whether data describe **technical or biological replicates**.	Yes	Figures

Ethics

Ethics	Information included in the manuscript?	In which section is the information available? (Reagents and Tools Table, Materials and Methods, Figures, Data Availability Section)
Studies involving **human participants**: State details of **authority granting ethics approval** (IRB or equivalent committee(s), provide reference number for approval.	Yes	Materials and Methods
Studies involving **human participants**: Include a statement confirming that **informed consent** was obtained from all subjects and that the experiments conformed to the principles set out in the WMA Declaration of Helsinki and the Department of Health and Human Services Belmont Report.	Yes	Materials and Methods
Studies involving **human participants:** For publication of **patient photos**, include a statement confirming that consent to publish was obtained.	Yes	Materials and Methods
Studies involving experimental **animals**: State details of **authority granting ethics approva**l (IRB or equivalent committee(s), provide reference number for approval. Include a statement of compliance with ethical regulations.	Not Applicable	
Studies involving **specimen and field samples:** State if relevant **permits** obtained, provide details of authority approving study; if none were required, explain why.	Not Applicable	

Dual Use Research of Concern (DURC)	Information included in the manuscript?	In which section is the information available? (Reagents and Tools Table, Materials and Methods, Figures, Data Availability Section)
Could your study fall under dual use research restrictions? Please check biosecurity documents and list of **select agents and toxins** (CDC): https://www.selectagents.gov/sat/list.htm	Not Applicable	
If you used a select agent, is the security level of the lab appropriate and reported in the manuscript?	Not Applicable	
If a study is subject to dual use research of concern regulations, is the name of the **authority granting approval and reference number** for the regulatory approval provided in the manuscript?	Not Applicable	

Reporting

The MDAR framework recommends adoption of discipline-specific guidelines, established and endorsed through community initiatives. Journals have their own policy about requiring specific guidelines and recommendations to complement MDAR.

Adherence to community standards	Information included in the manuscript?	In which section is the information available? (Reagents and Tools Table, Materials and Methods, Figures, Data Availability Section)
State if relevant guidelines or checklists (e.g., **ICMJE, MIBBI, ARRIVE, PRISMA**) have been followed or provided.	Not Applicable	
For **tumor marker prognostic studies,** we recommend that you follow the **REMARK** reporting guidelines (see link list at top right). See author guidelines, under 'Reporting Guidelines'. Please confirm you have followed these guidelines.	Not Applicable	
For **phase II and III randomized controlled trials**, please refer to the **CONSORT** flow diagram (see link list at top right) and submit the CONSORT checklist (see link list at top right) with your submission. See author guidelines, under 'Reporting Guidelines'. Please confirm you have submitted this list.	Not Applicable	

Data Availability

Data availability	Information included in the manuscript?	In which section is the information available? (Reagents and Tools Table, Materials and Methods, Figures, Data Availability Section)
Have **primary datasets** been deposited according to the journal's guidelines (see 'Data Deposition' section) and the respective accession numbers provided in the Data Availability Section?	Yes	Data Availability
Were **human clinical and genomic datasets** deposited in a public access-controlled repository in accordance to ethical obligations to the patients and to the applicable consent agreement?	Not Applicable	
Are **computational models** that are central and integral to a study available without restrictions in a machine-readable form? Were the relevant accession numbers or links provided?	Not Applicable	
If publicly available data were reused, provide the respective **data citations in the reference list**.	Not Applicable	
